# Unveiling the ecological processes driving soil and lichen microbiome assembly along an urbanization gradient

**DOI:** 10.1038/s41522-025-00736-4

**Published:** 2025-06-10

**Authors:** Panji Cahya Mawarda, Rens van der Kaaij, Francisco Dini-Andreote, Deniz Duijker, Michael Stech, Adrianus GCL Speksnijder

**Affiliations:** 1 Naturalis Biodiversity Center, Darwinweg 2, 2333 CR Leiden, The Netherlands; 2 Research Center for Applied Microbiology, National Research and Innovation Agency Republic of Indonesia (BRIN), KST Samaun Sadikun, Bandung, Indonesia; 3 Leiden Centre for Applied Bioscience, Hogeschool Leiden, Darwinweg 24, 2333 CR Leiden, The Netherlands; 4 Department of Plant Science & Huck Institutes of the Life Sciences, The Pennsylvania State University, University Park, PA 16802 USA; 5 The One Health Microbiome Center, Huck Institutes of the Life Sciences, The Pennsylvania State University, University Park, PA 16802 USA; 6 Leiden University, Leiden, The Netherlands

**Keywords:** Microbial communities, Environmental microbiology

## Abstract

Global biodiversity loss is accelerating due to the transformation of natural landscapes into agricultural and urban areas. Yet, research on the urbanization impact on environmental and host-associated microbiomes, particularly on the ecological processes that mediate their assembly and function, remains scarce. This study investigated the effects of an urbanization gradient on the diversity and assembly processes of the soil microbiome and the microbiomes of three epiphytic lichen species (*Candelaria concolor*, *Physcia adscendens*, and *Xanthoria parietina*). Our findings revealed that the urbanization gradient shaped the soil microbiome, while the lichen microbiomes exhibited strong host specificity and showed no significant changes in diversity along the urbanization gradient. Heterogeneous selection and dispersal limitation primarily governed the soil community assembly and higher community turnover in medium- and highly urbanized zones compared to low-urbanized zones, indicating an increased influence of environmental pressures, altered resources, and habitat fragmentation in more urbanized areas. The lichen microbiome assembly in each species was primarily governed by undominated processes regardless of urbanization level, indicating that both selection and stochasticity contributed to, but neither dominantly influenced, their assembly. The lichen microbiomes further revealed species-specific co-occurrence networks, with microbial compositional signatures and potential functions being essential for lichen fitness and urban ecosystem health. Taken together, our study contributes to understanding how microbial communities are assembled in urban environments, bridging the gap between conceptual theories and empirical findings in the urban ecology of soil and lichen-associated microbiomes.

## Introduction

Global biodiversity loss is accelerating at an unprecedented rate, mostly driven by the increasing urbanization of previous natural areas and ecosystem-scale landscape conversion for agricultural purposes^1,2^. Most importantly, landscape conversion is largely occurring in regions characterized as global biodiversity hotspots, including South America, Mesoamerica, and Southeast Asia^3,4^. The land use alteration directly affects ecosystem productivity and contributes to the release of xenobiotics, amplifying the impacts of global change^5–7^. Specifically in urban areas, the conservation and management of urban biodiversity is fundamental to protect species coexistence across fragments of their habitats. However, such effort requires a comprehensive understanding of species adaptation, assembly, and interactions across urban landscapes^8^.

Most studies that focused on ecological aspects of urban systems have primarily investigated animal and plant communities^9–11^. Relatively less attention has been given to studying the impact of urbanization on environmental and host-associated microbiomes^12–14^. In particular, we still lack information on how urban ecosystems affect the interplay of ecological processes structuring bacterial and fungal communities in association with macro-organisms^14^. Host-associated microbiomes play major roles in macro-organismal responses to environmental stresses. As such, gathering information on host-associated biodiversity responses to urbanization^15,16^ can provide information on the dynamics of ecological processes that enable urban microbiomes to persist, establish, and adapt to constant fluctuation in environmental abiotic conditions^14^.

Understanding how distinct processes modulate microbial community assembly is important to predict microbiome responses to biotic and abiotic perturbations—both in terms of changes in community structure and functioning^17–19^. The assembly of ecological communities can be summarized by the interplay of four high-level processes, namely selection, dispersal, diversification, and ecological drift^20^. Recently, studies have applied quantitative frameworks to estimate the relative influence of each of these processes across distinct microbial systems^17,21^. These frameworks allow determining the extent to which community assembly is predominantly modulated by deterministic (i.e., selection) or stochastic processes (i.e., random dispersal, diversification, and drift)^22–24^. Within deterministic processes, selection can operate via environmental filtering (i.e., as imposed by abiotic factors) or via species interactions that selectively structure microbial diversity and composition^25,26^. Within stochastic processes, however, random organismal dispersal and random shifts in species birth and death events (also known as ‘ecological drift’) can lead to unpredictable fluctuations in species abundance, influencing the microbiome assembly^26,27^. In the context of urban ecology, it is plausible to conceive that exploring the interplay of these processes, can provide critical information on the extent to which environmental stresses, landscape fragmentation, and infrastructure shape the ecology of urban biological communities^14^. Yet, despite its importance, very few studies explore this avenue^19^.

Here, we studied how an urbanization gradient affects the diversity and assembly of soil and lichen-associated microbiomes. Soil systems have long been used to study the impacts of anthropogenic activities on microbiota diversity across multiple natural- and managed-ecosystems. Although previous studies showed that urbanization exerts an effect on the soil microbiome^28,29^, we still lack information on the extent to which distinct ecological processes play roles in determining these changes, especially in lichen-associated microbiomes. In fact, lichens and their microbiomes, are important bioindicators in urban ecosystems, directly associated with environmental health. These include their use in monitoring soil and air quality and pollution^30,31^, (e.g., heavy metal contamination, sulfur and nitrogen deposition)^32,33^, and climate and environmental changes (e.g., urban heat island effect, chemical, and UV light concentration^34^). The bioindicator properties of lichens are attributed to their morphological characteristics, including the absence of roots and a cuticle, which allows them to absorb nutrients, contaminants, and gases directly from the atmosphere^33^. Lichens also lack protective tissues that would otherwise filter environmental substances, enabling the accumulation of these substances in their biomass^35,36^. Additionally, as lichens represent a bi- or tripartite symbiosis between fungi, green algae, and/or cyanobacteria, their associated microbiomes likely play a role in enhancing functional responses to changing environments^37,38^. For this study, we selected the lichen species *Candelaria concolor*, *Physcia adscendens*, and *Xanthoria parietina* due to their use in air quality biomonitoring and their adaptation in urban conditions^39,40^.

We hypothesized that the urbanization gradient would influence the structure, predicted functions, and co-occurrence of bacterial and fungal taxa in soil and in the lichen-associated microbiomes. In addition, we expect deterministic processes to exert a stronger influence in highly urbanized areas compared to the low ones. To test these hypotheses, we analyzed both bacterial and fungal communities in soils and the three lichen species collected across a gradient of low, medium, and high urbanization zones.

## Results

### Soil microbiome variation across the urbanization gradient

**Fig. 1:**
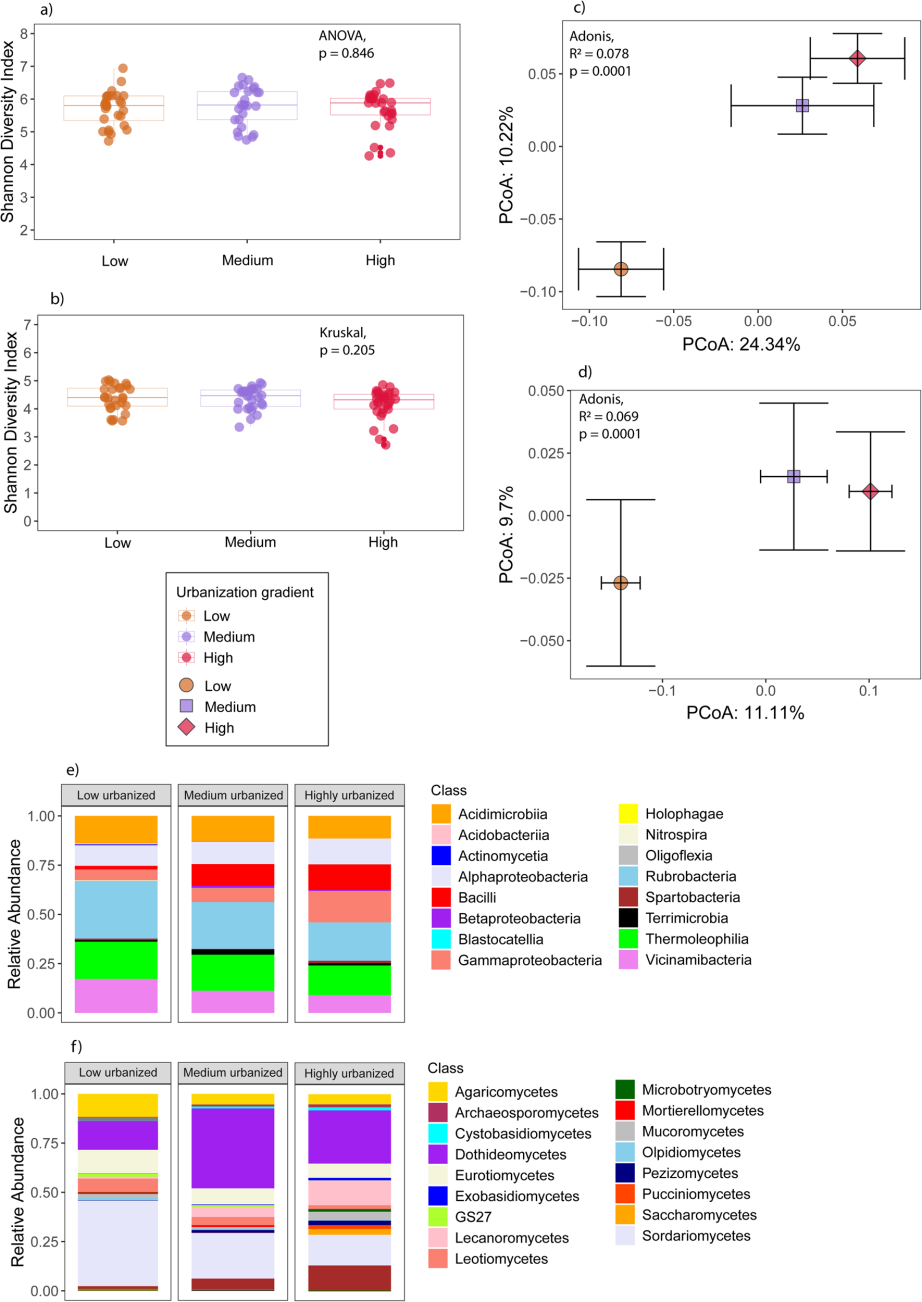
Overview of soil bacterial and fungal community diversity across the urbanization gradient. Alpha diversity is represented by the Shannon diversity index for **a** the soil bacterial community and **b** the soil fungal community. Community structure is depicted using PCoA plots based on Bray–Curtis distances for **c** the soil bacterial community and **d** the soil fungal community. The PCoA plots (**c**, **d**) demonstrate the effect of the urbanization gradient on community structure, with significant differences determined by PERMANOVA (*p* < 0.001). Centroids for each urbanization level are displayed with their standard errors (error bars). Meanwhile, taxonomic composition is shown as class-level bar plots for **e** the soil bacterial community and **f** the soil fungal community.

The urbanization gradient (low-, medium-, and highly urbanized zone) did not result in differences in soil bacterial and fungal species richness (Supplementary Fig. 1a, b) and the Shannon diversity index (Fig. 1a, b) (ANOVA, *p* > 0.05). However, the gradient significantly changed the structure of these soil communities (Fig. 1c, bacteria: Adonis, *R*² = 0.078, *p* = 0.0001; Fig. 1d, fungi: Adonis, *R*² = 0.069, *p* = 0.0001). Both bacterial and fungal community structures formed two separated clusters (pairwise-Adonis, *p* < 0.05) based on urbanization level: (1) communities in the low zone, and (2) communities in the medium and high zones (Fig. 1c, d). The community turnover in the medium and high zones was significantly higher than in the low zone (Supplementary Fig. 1c, d, ANOVA, *p* < 0.05). Taxonomic analysis at the class level (Fig. 1e, f) also revealed that the medium and high zones exhibited higher community similarities. For instance, they shared similar relative abundances of Acidimicrobiia (~10%), Alphaproteobacteria (~7%), and Bacilli (~4%) (Fig. 1e), and contained fungal classes that were absent in the low zone, such as Pucciniomycetes, Saccharomycetes, and Pezizomycetes (Fig. 1f).

We also examined which soil microbial taxa significantly changed over the urbanization gradient. Our results revealed a total of 180 out of 10,649 soil bacterial taxa and 151 out of 9419 soil fungal taxa to account for the dynamics of soil microbiome across this gradient (Supplementary Table 1a, b). These subsets showed response similarity with the original dataset, based on significant correlation in symmetric Procrustes rotation (Bacteria: 9999 permutations, *R*^2^ = 0.8292, *p* = 0.0001 and Fungi: 9999 permutations, *R*^2^ = 0.8338, *p* = 0.0001), thereby establishing a statistically valid representation of the original community.

**Fig. 2:**
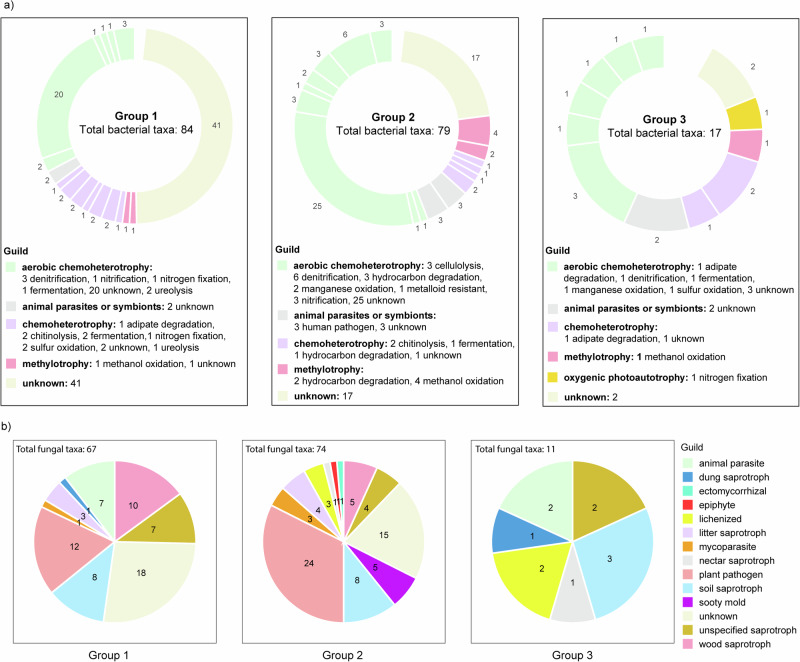
Functional guilds of soil bacterial and fungal communities with significant changes in abundance across the urbanization gradient, as determined by ANOVA (*p* < 0.01). Functional guilds of the soil bacterial community (**a**) and fungal community (**b**), categorized into those significantly more abundant in low-urbanized areas (Group 1), in medium and highly urbanized areas (Group 2), and exclusively in highly urbanized areas (Group 3).

To uncover possible spatial-driven trajectories across the gradient, we clustered these microbiomes based on their peak abundance along the gradient. The results revealed three major bacterial and fungal groups that represented taxa displaying higher relative abundance (1) only in the low zone (group 1); (2) in the medium and high zones (group 2), and (3) only in the high zone (group 3). Detailed information about the bacterial and fungal taxa associated with these groups and their predicted guild is available in Table S2a, b. A higher number of bacterial and fungal taxa were present in groups 1 and 2 compared to group 3, with totals of 84, 79, and 17 bacterial taxa and 67, 74, and 11 fungal taxa, respectively (Fig. 2a, b). Groups 1 and 2 consisted of bacterial and fungal taxa with more diverse predicted guilds than group 3. The microbiome composition within each group may highlight spatially distinct urban ecological niches. For example, fungal communities predicted as lichenized fungi (e.g., *Acarospora* spp.*, Lepraria santosii*), sooty mold (*Aureobasidium* spp.), ectomycorrhizal (*Otidea cantharella*), and nectar saprotroph (*Dedbaryomyces hansenii*) significantly peaked in relative abundance in the medium and high zones (only present in groups 2 and 3). Bacterial taxa predicted as hydrocarbon degraders (e.g., *Acinetobacter colistiniresistens, Pseudomonas alcaligenes, Methylocystis rosea, Hydrocarboniphaga daqingensis, Halomonas cerina*, and *Methyloparacoccus murrellii*) were only present in group 2, whose abundance peaked in the medium- and highly urbanized zones. Group 2 also included bacteria with the predicted capability of oxidizing manganese and showed resistance to heavy metals (Fig. 2a).

### Host specificity of lichen microbiomes across the urbanization gradient

**Fig. 3:**
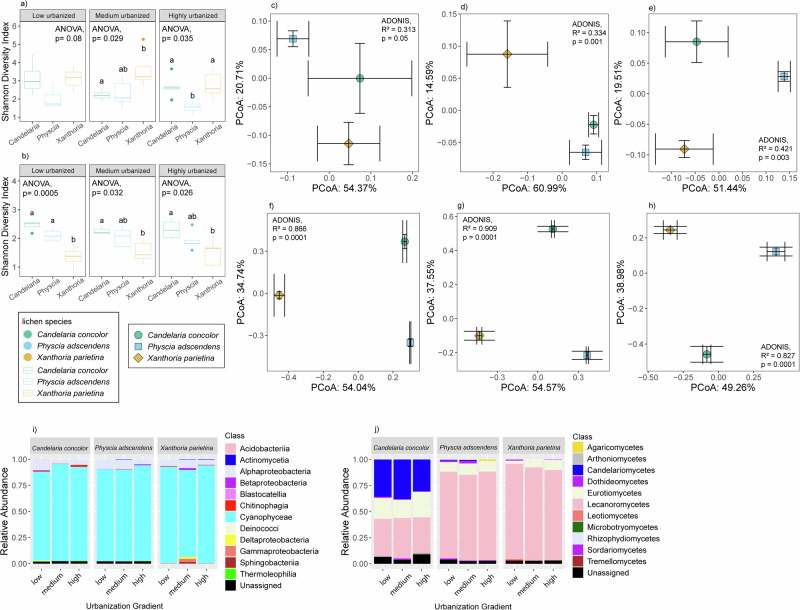
Overview of lichen bacterial and fungal community diversity across the urbanization gradient. Alpha diversity is shown using the Shannon diversity index for **a** the lichen bacterial community and **b** the lichen fungal community. The box plots show that lichen bacterial communities differ by lichen species in medium and highly urbanized areas, while lichen fungal communities differ by lichen species across all urbanization levels (ANOVA, *p* < 0.05). Community structure is depicted using PCoA plots based on Bray–Curtis distances for the lichen bacterial community in (**c**–**e**) and the lichen fungal community in (**f**–**h**) across low, medium, and highly urbanized areas, respectively. The PCoA plots (**d**, **e**) reveal the influence of lichen species on bacterial community structure, with significant differences in medium and highly urbanized areas (PERMANOVA, *p* < 0.001). Meanwhile, PCoA plots (**f**–**h**) show the effect of lichen species on fungal community structure, with significant differences across all urbanization levels (PERMANOVA, *p* < 0.001). Centroids for each urbanization level are displayed with their standard errors (error bars). Additionally, taxonomic composition among lichen species is shown as class-level bar plots for **i** the lichen bacterial community and **j** the lichen fungal community.

The urbanization gradient had no impact on the lichen microbiome diversity and structure (Supplementary Fig. 2a‒h, ANOVA, *p* > 0.05). Instead, the lichen microbiome differed between the lichen species, thus showing high levels of host specificity (Fig. 3a–h, ANOVA, *p* < 0.05). For example, the Shannon diversity index of fungal communities in *Candelaria* was higher than in *Xanthoria*, across all zones (Fig. 3b, Tukey’s post hoc, *p* < 0.05). The fungal community structure in these lichens also differed from each other in all three zones (Fig. 3f‒h, pairwise-Adonis, *p* < 0.05). Differences were also observed in the taxonomic composition of the microbiomes of each lichen species (Fig. 3j). In *Candelaria*, taxa belonging to Lecanoromycetes and Candelariomycetes were approximately equally dominant, whereas in *Physcia* and *Xanthoria* only Lecanoromycetes were dominant, accounting for 85‒95% of the community composition.

**Fig. 4:**
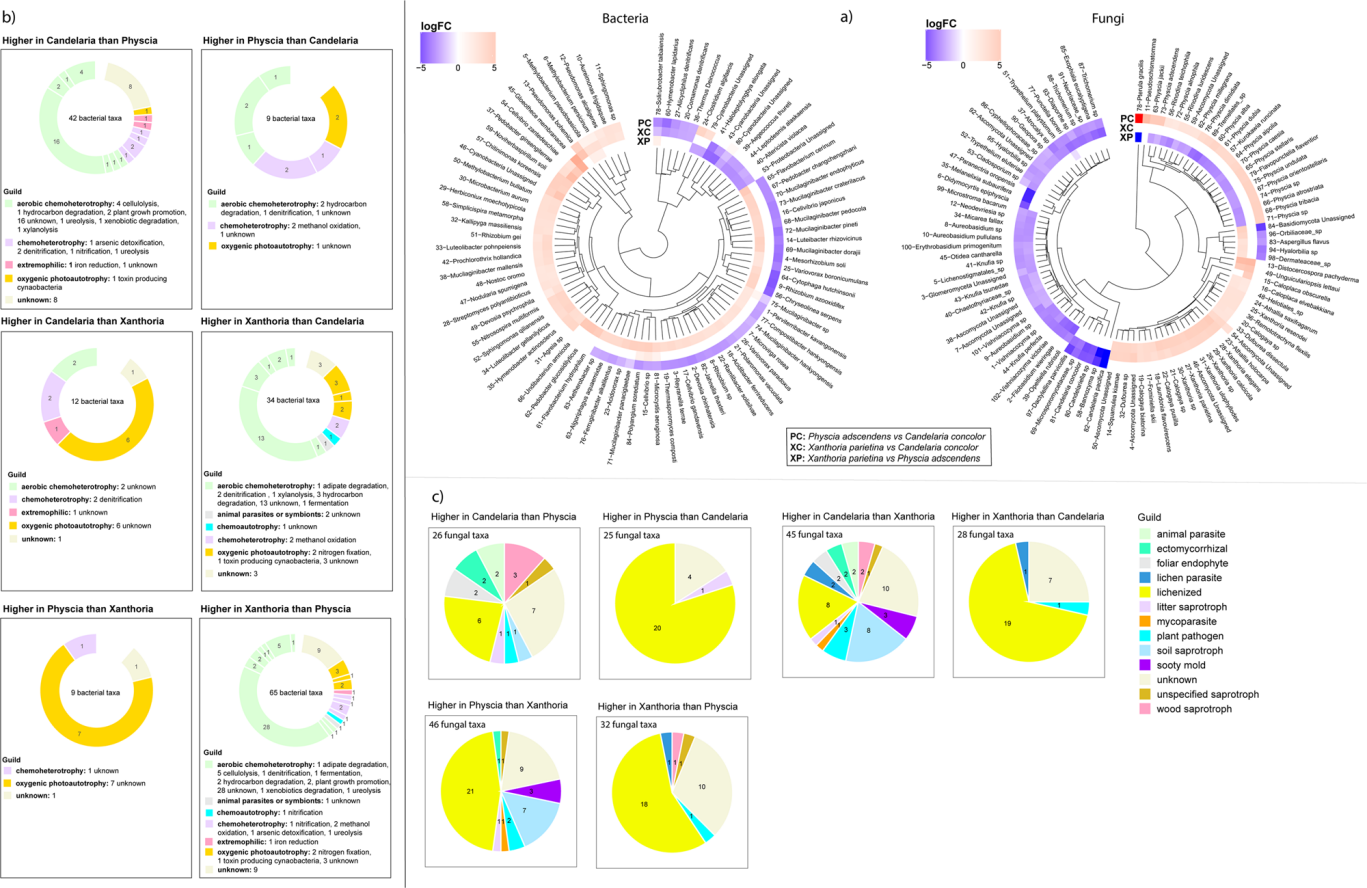
Differential abundance and functional guilds of lichen bacterial and fungal communities across different lichen species. **a** Taxonomic composition of lichen bacterial and fungal communities showing significant differences in abundance between *Candelaria* and *Physcia*, *Candelaria* and *Xanthoria*, and *Xanthoria* and *Physcia*. **b** Overview of functional guilds in lichen bacterial and **c** in lichen fungal communities with significant abundance differences among the same lichen species pairs.

Fungal differential abundance analyses revealed *Candelaria* to have a higher relative abundance of diverse guilds than *Xanthoria* and *Physcia* (Fig. 4a,c, DESeq2, Wald test, *p* < 0.05). In brief, *Candelaria* had higher relative abundance of fungal taxa classified as wood saprotroph, ectomycorrhizal, foliar endophyte, soil saprotroph, plant pathogen, lichen parasite, animal parasite, mycoparasite, and lichenized fungi. On the other hand, *Physcia* and *Xanthoria* had higher relative abundance of lichenized fungi. This analysis revealed that the composition of lichenized fungi was specific to each lichen species, including the main mycobiont as dominant component (Fig. 4a, DESeq2, Wald test, *p* < 0.05). In addition, other lichen genera (e.g. *Calogaya*, *Caloplaca*, *Melanelixia*) were also found (Fig. 4a). Further information about fungal community differences and their associated guilds across lichen species is available in Supplementary Table 2a, b.

As for lichen-associated bacterial communities, this host-specificity diversity pattern was less pronounced, being affected by the urbanization gradient. For instance, the Shannon diversity of bacterial communities in *Xanthoria* was higher than *Candelaria* and *Physcia* in the medium and high zones, respectively (Fig. 3a, Tukey’s post hoc, *p* < 0.05), but not in the low zone (Fig. 3a, ANOVA, *p* > 0.05). We also observed the same pattern in bacterial community structure, where the differences across lichen species were only significant in the medium (Adonis, *R*² = 0.334, *p* = 0.001) and high zones (Adonis, *R*² = 0.421, *p* = 0.003), but not in the low zone (Adonis, *R*² = 0.313, *p* > 0.05) (Fig. 3d, e). In the medium zone, the bacterial community structure in *Xanthoria* clustered separately from those in *Physcia* and *Candelaria* (pairwise-Adonis, *p* < 0.05), while the latter grouped together (pairwise-Adonis, *p* > 0.05). In contrast, the bacterial community structures among all three lichen species differed significantly from each other in the high zone (pairwise-Adonis, *p* < 0.05). In all zones, the bacterial communities of each lichen were dominated by Cyanobacteria (class Cyanophyceae), suggesting a symbiotic relationship of these chlorolichens with this particular bacterial group.

Differential abundance analyses of bacterial communities corroborate previous results by showing the species *Xanthoria* to have the greater abundance of diverse guilds compared to *Candelaria* and *Physcia* (Fig. 4a,b, DESeq2, Wald test, *p* < 0.05). These bacteria were classified in the guild of aerobic chemoheterotrophy, chemoheterotrophy, chemoautotrophy, oxygenic photoautotrophy, extremophiles, and animal parasites or symbionts. They are predicted to play roles in hydrocarbon degradation, arsenic detoxification, plant growth promotion, nitrogen fixation, nitrification, denitrification, xylanolysis, cellulolysis, etc. The bacterial taxa with higher relative abundance in *Candelaria* and *Physcia* were predominantly classified as oxygenic photoautotrophs with fewer known functional capabilities, compared to those in *Xanthoria*. Detailed taxonomic information and the associated guilds of bacterial taxa with significantly higher or lower relative abundances across lichen species are available in Supplementary Table 2a, b.

Spatial trajectory analyses showed that in line with the lack of urbanization effect on the lichen microbiome diversity, the lichen microbiome dynamics across the urbanization gradient confirmed a low number of taxa representation (Supplementary Fig. 3). These representative taxa did not significantly account for the changes in the total community structure (symmetric procrustes rotation, *p* > 0.05). The result thus suggests that the lichen microbiome was primarily driven by host specificity—i.e., the lichen species—rather than the urbanization gradient.

### Ecological processes modulating the soil and lichen microbiome assembly

**Fig. 5:**
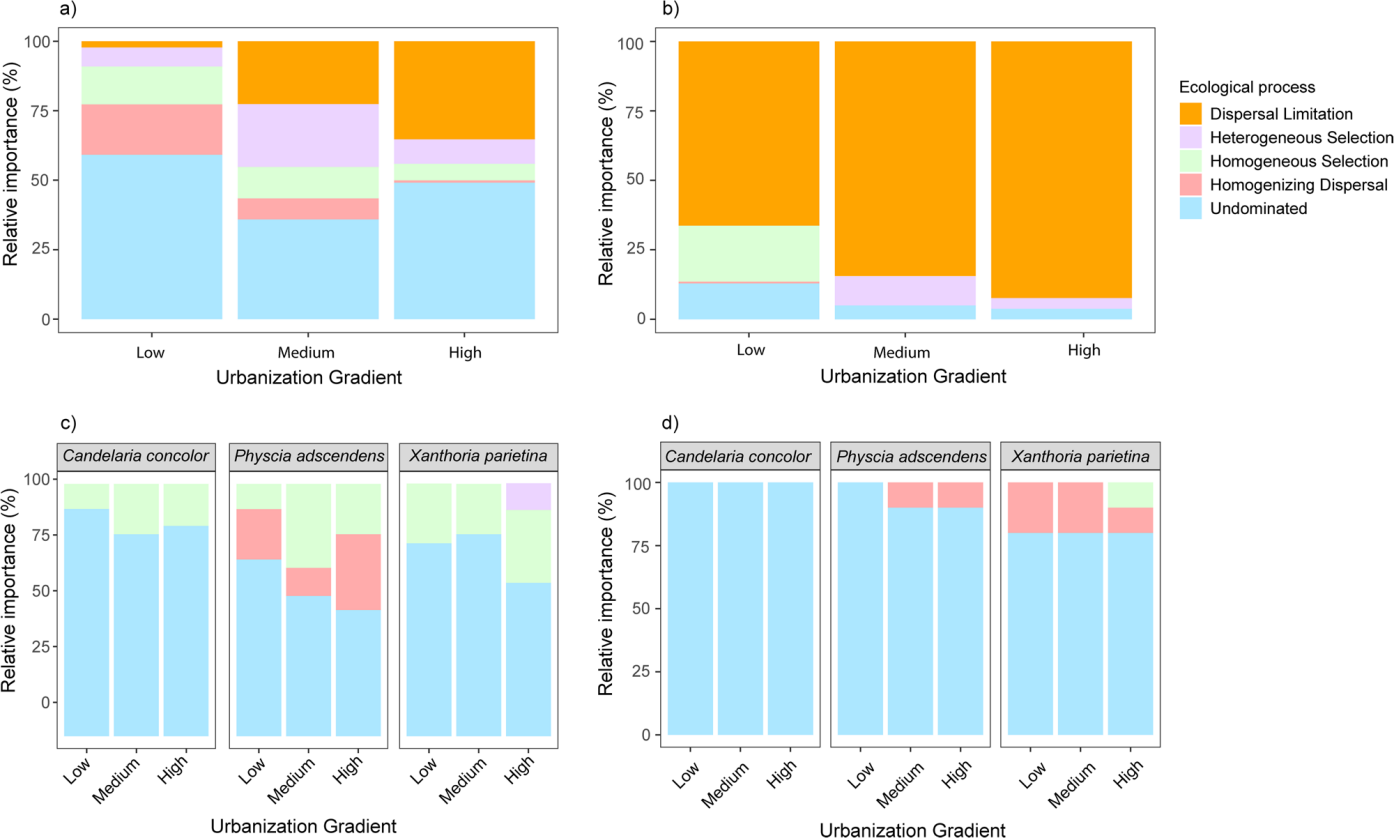
The interplay of ecological processes driving the assembly of soil and lichen microbial communities. Bar plots illustrate the relative contribution of assembly processes to the spatial variation of soil bacterial (**a**) and fungal (**b**) communities across the urbanization gradient (low, medium, high), and of lichen bacterial (**c**) and fungal (**d**) communities across lichen species (i.e., *Candelaria concolor, Physcia adscendens*, and *Xanthoria parietina*) at the three urbanization level. The βNTI and RC_bray_ values were used to quantify the relative importance of each assembly process. Colors represent different assembly processes, i.e., heterogeneous selection, homogeneous selection, dispersal limitation, homogenizing dispersal, and undominated processes.

The results from the phylogenetic null model revealed that distinct ecological processes interplay in determining the assembly of soil and lichen-associated microbiomes across the urbanization gradient. In the soil, bacterial communities were partly structured by dispersal limitation, which increased along the gradient (from 2% in the low zones to 22.64% and 35.32% in medium and high zones, respectively) (Fig. 5a). Yet, dispersal limitation was more important in fungal communities, with a relative influence of >60% in each urbanization zone (Fig. 5b). Similar to soil bacteria, this effect also intensified along the urbanization gradient, increasing from 62.5% in the low zone to 75% in the medium zone, and reaching >80% in the high zone. The increasing contribution of dispersal limitation was accompanied by a reduction in homogenizing dispersal in the bacterial community, which dropped from 18.18% in low zone to 7.5% in the medium zone, and further to 1.08% in high zone. For the soil fungal community, homogenizing dispersal only accounted for 1% of the total community assembly in the low zone.

The contribution of homogeneous and heterogeneous selection varied across the urbanization gradient in both soil bacterial and fungal communities (Fig. 5a,b). Interestingly, selection influenced bacterial community assembly stronger than fungal community assembly. For soil bacterial communities, heterogeneous selection contributed more to the community assembly in the medium zone (22.64%), as compared to 6.82% in the low and 10.43% in the high zone. In contrast, homogeneous selection decreased progressively along the gradient, from 13.63% in the low zone to 11.3% in medium zone, and 5.97% in high zone. As for soil fungal communities, homogeneous selection only contributed to the community assembly in the low zone (25%). Although heterogeneous selection was absent in the low zone, this was an important process in the medium and high zones, contributing 12.5% and 5%, respectively. It is important to note that the undominated process predominantly governed the assembly of soil bacterial communities in the low and high zones, accounting for 59.09% and 52.17%, respectively.

The assembly of lichen-associated bacterial and fungal communities was mostly governed by the undominated process, which accounted for over 60% in all lichen species regardless of the urbanization zone (Fig. 5c,d). Other ecological processes had a species-specific influence rather than being determined by the urbanization gradient. For example, in *Candelaria*, homogeneous selection contributed ca. 20‒25% to the bacterial community assembly In *Physcia*, homogenizing dispersal (11‒30%) and homogeneous selection (10‒33.33%) were found to be more important, whereas in *Xanthoria* heterogeneous and homogenous selection governed 10‒30% of the assembly, respectively. As for lichen fungal communities, undominated process governed the fungal assembly more than 75% in all lichen species, with a small influence of homogenizing dispersal in *Physcia* and *Xanthoria*.

### Taxa co-occurrence in the soil microbiome across the urbanization gradient

**Fig. 6:**
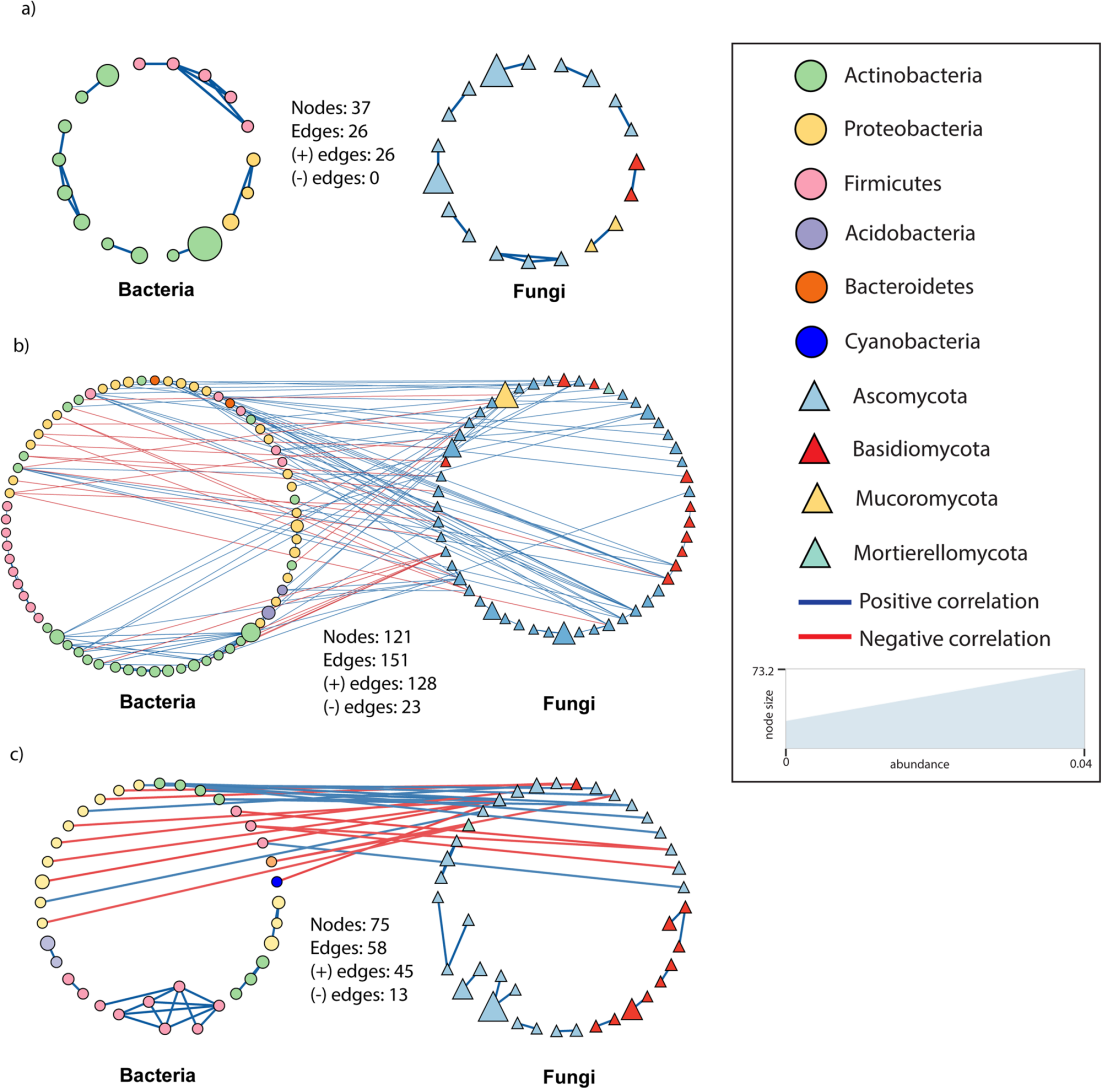
Co-occurrence network between soil bacterial and fungal communities across **a** low, **b** medium, and **c** highly urbanized areas. Blue and red colors of the edges represent positive and negative relationships, respectively, while the color of nodes demonstrates bacterial and fungal phyla. The shape of vertices reflects microbial domains (i.e., circle: bacteria; triangle: fungi), and the size of nodes refers to abundances.

The network analysis revealed differences in species co-occurrences across the urbanization gradient. For example, soil microbial communities from the medium zone exhibited the most complex microbial network connectivity, with the highest number of edges, nodes, diameter, radius, and heterogeneity (Fig. 6a and Supplementary Table 3), followed by those in high and low zones (Fig. 6b, c). The numbers of both positive and negative correlations between microbial taxa were higher in the medium zone, with 128 positive and 23 negative edges, followed by the high zone with 45 positive and 23 negative edges, and the low zone with 26 positive and 0 negative edges (Fig. 6a‒c). This coincides with the result above, where heterogeneous selection influences the community assembly more in medium zone.

Results from the soil bacterial-fungal interactions alone, also exhibited the highest number of soil bacterial-fungal correlations in the medium zone, with 61 positive interactions and 23 negative interactions. This was followed by the high zone displaying 12 positive and 13 negative interactions, while the low zone did not show significant co-occurrence between bacterial-fungal taxa. Detailed information on correlations between bacterial and fungal taxa across the urbanization gradient is available in Supplementary Table 4.

### Taxa co-occurrence and symbiotic signatures in lichen species

Co-occurrence networks based on the lichen-associated microbiomes revealed species-specific patterns, with *Candelaria* having the most complex network (Supplementary Fig. 4a and Supplementary Table 5), followed by *Xanthoria* and *Physcia* (Supplementary Fig. 4b, c). *Candelaria* had the highest number of correlations with 113 positive and 62 negative interactions, compared to 63/11 in *Xanthoria* and 59/14 in *Physcia* (Supplementary Fig. 4a‒c). The identity of these bacterial and fungal taxa on each lichen species is available in Supplementary Table 5.

**Fig. 7:**
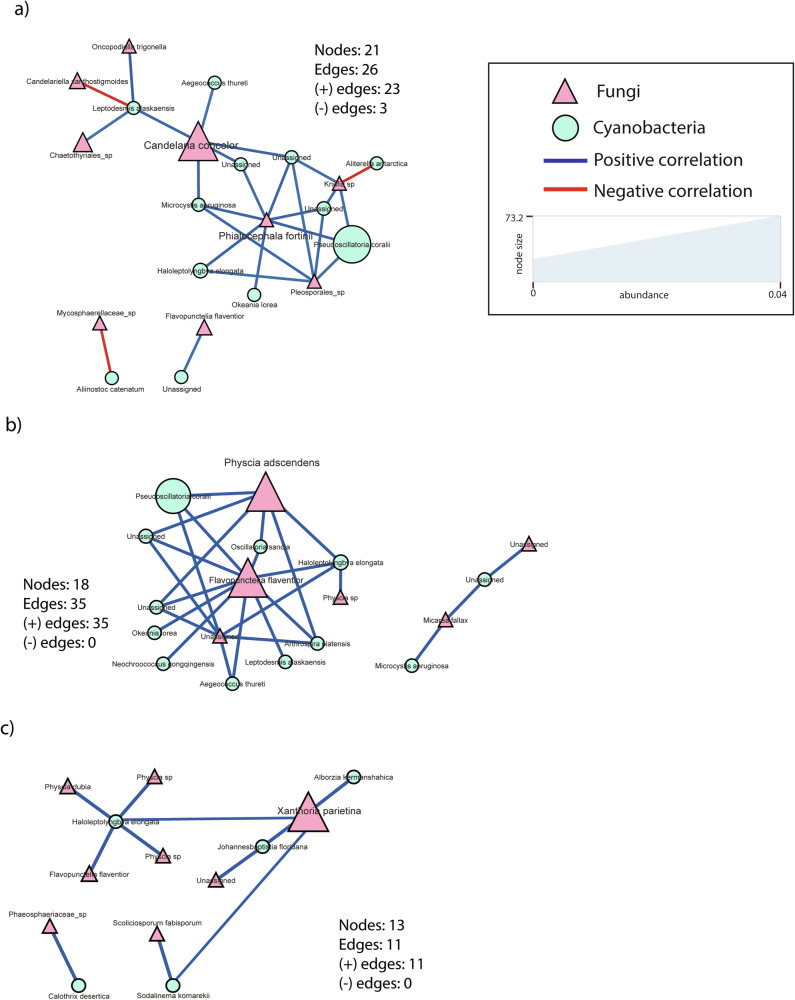
Co-occurrence network between mycobiont and cyanobiont communities in **a** *Candelaria concolor*, **b** *Physcia adscendens*, and **c** *Xanthoria parietina*. Blue and red colors of the edges represent positive and negative relationships, while pink and green colors of nodes represent the mycobiont and cyanobiont communities respectively. The shape of vertices reflects microbial domains (i.e., circle: bacteria; triangle: fungi), and the size of nodes refers to abundances. In addition, the nodes with higher font size were assigned as hub taxa.

Although all three studied lichen species are chlorolichens, the analysis of co-occurrence between bacterial and fungal taxa showed correlations between the mycobionts and cyanobacteria, which were specific to each lichen species (Fig. 7a–c). In *Candelaria concolor*, there were two hubs of fungal taxa, the mycobiont and *Philacheohala fortini*. The former positively correlated with cyanobacterial taxa such as *Leptodesmis alaskaensis*, *Aegeococcus thureti*, *Microcystis aeruginosa*, while the latter had positive correlations with *Haloleptolyngbya elongata*, *Okeania lorea*, *Pseudoscillatoria coralii*, and three other unidentified cyanobacteria. Regarding *Physcia adscendens*, the mycobiont hub positively correlated with *Pseudoscillatoria coralii*, *Haloleptolyngbara elongata, Oscillatoria sancta, Anthrospira platensis*, and another lichenized fungal hub, *Flavopunctelia flaventior*, positively correlated with *Okeania lorea, Neochroococcus gongqingensis, Aegeococcus thuretti, Leptodesmis alaskaensis, Anthrospira platensis, Pseudoscillatoria coralii*, *Haloleptolyngbara elongata*, and *Oscillatoria sancta*. Last, in *Xanthoria parietina*, the mycobiont hub positively correlated with *Johanesbaptistia floridana* and *Alborzia kemanshahica*. Interestingly in *Xanthoria*, we also detected a cyanobacterial hub taxon, i.e., *Haloleptolyngbara elongata* that positively correlated with the additional lichenized fungi *Physcia dubia*, *Physcia* sp., and *Flavopunctelia flaventior*.

## Discussion

The average increase in the global urban population and expansion of urban infrastructure have contributed to drastic environmental changes^1^. Assessing such impact on urban biodiversity and ecosystem functioning—particularly the urban microbiome, which plays a crucial role in maintaining urban environmental health—is essential. In this study, we thus examined the microbiome structure in urban soil and lichen across different urbanization levels, using ecological principles related to community diversity, dynamics, and the ecological processes governing community assembly.

This study corroborates previous findings showing that urbanization has a direct impact on soil microbial communities by dynamically altering the diversity structure and patterns of taxa co-occurrence across distinct levels of urbanization^28,29^. Urbanization levels likely create distinct environmental and edaphic conditions that alter the soil microbiome through varying degrees of microclimatic conditions, resource availability, and above- and belowground plant biomass^41,42^. Moreover, urban soils are formed during the urbanization process and primarily driven by human activities, for which the soil formation and evolution occurs at a much faster rate in urban than rural soils^43^.

In addition, urban soils often accumulate multiple chemical pollutants from industrial and traffic sources, leading to further changes in soil properties and resources^19,44^, and are also subject to compaction from trampling and traffic^45^. This aligns with our findings, which showed an increase in bacterial communities involved in hydrocarbon degradation and heavy metal resistance in the medium and highly urbanized areas, as these environmental pressures may select for taxa with these metabolisms. We also observed some sooty mold, ectomycorrhizal, and nectar saprotroph fungi to increase in relative abundance in urban soils. The unique guilds of these microbial taxa across the urbanization gradient likely associate with distinct microbial niches in urban environment and may indicate microbiome adaptation to various environmental factors^46,47^.

We found higher turnover in soil bacterial and fungal communities in the more urbanized zones, which corroborates previous findings^28,48^. In our study, this can be explained by the relative contribution of distinct ecological processes modulating community assembly. For example, our results revealed a decreasing influence of homogenizing dispersal and an increase of dispersal limitation from the outskirt (low urbanized area) to the city center (highly urbanized area), indicating an overall reduction in species dispersal that cause an increase in community spatial turnover with increasing urbanization^24,49^. This pattern may result from increasing habitat fragmentation, as in our study, soil samples in city centers are more isolated in small tree pits compared to low urbanized zone where soil samples were taken in larger open areas. Habitat fragmentation in urban areas restricts species movement, isolating habitats and reducing species interactions, allowing communities to drift apart^24,50^. Meanwhile, a strong influence of homogenizing dispersal in rural soils reduces species spatial variability, resulting in communities that are more similar to each other^21,51^. The latter has indeed been observed in microbial communities inhibiting relatively stable environments like rural areas^52^.

In our system, dispersal limitation had a greater influence on fungal communities (>75%) compared to bacterial communities, while selection influenced bacterial communities more than fungi. This is in line with the “size-dispersal” and “size-plasticity” hypotheses, which differentiate community assembly mechanisms between organisms^53,54^. According to the size-dispersal hypothesis, fungi typically have larger bodies and propagule sizes compared to bacteria, which reduces their dispersal potential. Consequently, fungal community assembly is more strongly influenced by dispersal limitations. In contrast, the size-plasticity hypothesis suggests that bacteria, with higher metabolic flexibility, are more likely to be influenced by selection pressures, leading to bacterial communities being more prone to assemble via deterministic processes^53–55^.

We also detected the relative influence of homogenous selection to decrease across the urbanization gradient. Homogenous selection drives community similarity due to the presence of one or a few stringent factors imposing selection^17^. Moreover, when both biotic and abiotic environmental conditions are spatially similar, the selective environment will also be spatially homogenous^20^. This is the case for the outskirt/rural soils where heterogeneity due to habitat fragmentation is lower, whereas urban regions experience greater variations in edaphic environmental factors—such as pollution, xenobiotics, soil degradation, and changes in temperature and moisture regimes^56,57^. Variable selection increases community turnover as environmental conditions change spatially^20,21^. Our findings supported this notion as community turnover and bacterial taxa involved in xenobiotics degradation, heavy metal oxidation, and hydrocarbon degradation were found to be enriched in urban zones as compared to the outskirts area.

Yet interestingly, heterogeneous selection appeared to have a greater influence in medium-urbanized areas for both soil bacterial and fungal communities. We surmised that the stronger impact of dispersal limitation in the highly urbanized zone may override the selection process, as dispersal limitation can enhance stochasticity and mask the relationships between environmental pressures and microbial community composition, as demonstrated by these studies^58,59^. Moreover, the relative importance of selection in comparison to other assembly processes may vary depending on spatial heterogeneity and dispersal rates within metacommunities^60^. In our study, variable environmental pressures could also be prominent in suburban areas, which are densely populated and experience significant anthropogenic activities as well. This suggests that various environmental pressures, particularly those related to domestic households, may be high and substantial in these areas. Future studies must examine both biotic and abiotic factors in these sampling areas to further confirm this notion.

Our findings further revealed that heterogeneous selection was linked with higher number of taxa co-occurrence, particularly in the medium zone, followed by the high and low zones. This occurs because both positive and negative correlations between taxa in the networks tend to increase in systems displaying high community turnover, driven by greater fluctuations in species relative abundances across space or over time^61^. Moreover, higher community turnover leads to greater spatial or temporal niche partitioning, which directly influences species diversity and networks within the system^62,63^. These networks may reflect the similar responses of co-occurring species to environmental fluctuations, as well as co-metabolism among interacting taxa^64^.

Regarding the lichen microbiome, the diversity, dynamics, and predicted guild varied between the studied lichen species rather than along the urbanization gradient. This result aligns with previous studies showing high host specificity in lichen-associated microbiomes^65–68^. This suggests that lichen species may have the ability to recruit specific microbes from their surroundings and have evolved traits that shape their microbiomes^69,70^. For instance, some studies have shown that lichen’s secondary metabolites and thallus morphology can affect the lichen microbiome structure and diversity^71–73^. Indeed, during lichenization—the formation of a lichen from fungal spores through the growth of fungal hyphae, capturing algal cells and differentiation of the lichen thallus—symbiotic partners in lichens release a diverse array of metabolites. These include fungal lectins, algal cyclic peptides, phytohormones, antioxidants, sugars, and sugar alcohols, which dynamically influence the structure of the lichen microbiome^71,74^. For instance, glucose, ribitol, and mannitol produced by lichen-forming *Trebouxia* alga were shown to trigger the growth of Alphaproteobacteria taxa associated with lichen photobionts^75^. Additionally, lichen metabolites can induce bacterial biosynthesis of exometabolites that may not directly benefit the bacteria but contribute to the overall function of the lichen. For example, cyanobacterial photobionts in lichen produce distinct metabolites from those of free-living cyanobacteria, including specific types of microcystin and nosperin, which promote lichen protection against predators^76,77^. In terms of lichen taxonomy, the fungal diversity observed in our study reflects the revised taxonomic classification by Voglmayr et al.^78^ in which *Candelaria* related taxa were separated at class level from the Lecanoromycetes. However, a signature of Lecanoromycetes fungi is still present in *Candelaria* according to our data.

Specific lichen-associated microbiomes likely play a role in enhancing stress tolerance by supporting adaptive traits in response to changing environmental conditions. For instance, previous studies observed compositional shifts of lichen-associated microbiomes due to climate warming, variations in annual precipitation, and temperature seasonality^79,80^. In terms of adaptation to urban environments, although in our study the urbanization—using the urban heat island effect as a proxy—did not affect the lichen microbiome diversity, previous research showed that these communities were shaped by typical urban environmental stressors, including heavy metal pollution, hydrocarbon contamination, and desiccation^81,82^. Our results found an increase of lichen bacteria capable of detoxifying arsenic in *Xanthoria parietina* and *Candelaria concolor*. In fact, *X. parietina* has been shown to tolerate high concentrations of heavy metals by sequestering them extracellularly and binding metal cations to the cell walls of the associated bacterial and fungal taxa^83,84^. Other lichens detoxify heavy metals by producing metal oxalates, which chelate and neutralize the excess metals^85^. Additionally, we found an increase of bacterial communities capable of performing xenobiotics and hydrocarbon degradation in *Candelaria* and *Xanthoria*. Indeed, some of the lichen-associated bacteria has been demonstrated to degrade naphthalene and anthracene^86^.

In line with host specificity of the lichen microbiome, the assembly process also showed species-specific patterns. This result corroborates the notion that the relative importance of assembly process differs across host organisms and their habitats^87–89^. Interestingly, we observed that undominated process, mostly contribute to both fungal and bacterial community assembly in each lichen species. This implies that multiple ecological processes, i.e., selection, dispersal, drift, and diversification, contribute to shape the community assembly^21,51^. In other words, the community structure results from a balance or interaction of these processes. However, though minimal, we still observed a significant influence of selection and dispersal across these three lichen species. This finding corroborates with previous studies showing that microbiome assembly in lichens is a result of multiple stochastic and deterministic processes including lichen propagule dispersal, bacterial ‘rains’, nutrients, substrate, surrounding microbiome, lichen metabolites, etc.^37,90–92^.

Since the diversity, dynamics, and assembly of the lichen-associated microbiomes differed across species, we further examined the taxa co-occurrence network to detect possible symbiosis signatures in each lichen species. In line with previous studies, our finding showed that each lichen microbial network consists of diverse, taxon-specific, and some overlapping taxa^68,93^. The main mycobiont i.e., *Candelaria concolor*, *Xanthoria parietina*, and *Physcia adscendens*, appeared as a fungal hub species in each respective lichen. This may contribute to the morphological and physiological differences between these lichens, as the mycobiont is responsible for regulating and shaping the thallus morphology and controls the photobiont’s (green algae in the studied lichens) exposure to sunlight during physiologically active stages^94,95^. Yet, it is important to mention that we did not target algal communities in our study. These photobiont communities produce energy-rich carbohydrates for the mycobiont and microbiome of lichens to metabolize^96–98^.

The network and abundance analyses suggest that bipartite chlorolichens may have structural associations with cyanobacteria as well. Tagirdzhanova et al.^99^ found high cyanobacterial signatures not only in cyanolichens of the order Peltigerales, but also in the Teloschistales, to which *Physcia* and *Xanthoria* belong. However, these signals were consistently present only in raw bacterial rRNA gene sequences, not in the assembled metagenome reads. Our finding of dominant cyanobacterial occurrence in *Candelaria* (order Candelariales, not included in Tagirdzhanova et al.^99^) may indicate that cyanobacteria are even more widely associated with chlorolichens as known so far^32,89,90^. Similar to the phycobiont, the cyanobiont of (cyano)lichens can also provide carbon sources through photosynthesis. However, in the lichen system, they are primarily responsible for nitrogen fixation^98,100^. As shown in our study, the main mycobiont in each lichen species positively interacted with cyanobacteria taxa capable of fixing nitrogen, such as *Neochroococcus gongqingensis, Aegeococcus thuretti, Leptodesmis alaskaensis, Anthrospira platensis, Pseudoscillatoria coralii*, and *Haloleptolyngbara elongata*. It is also interesting that, besides the main mycobiont, other fungal taxa (e.g., the saprotroph *Phialocephala fortinii* in *Candelaria*, and a lichenized fungus *Flavopunctelia flaventior* in *Physcia* and *Xanthoria*) are also positively correlated with these nitrogen-fixing cyanobacterial taxa. Previous studies have shown that *Phialocephala fortinii* significantly enhances plant biomass, phosphorus, and nitrogen uptake^101–103^. In addition, the abundance of *Flavopunctelia flaventior* has been shown to positively correlate with excessive nitrogen deposition, making it an indicator species for ammonia pollution, commonly known as a nitrophyte^104^, further supporting its symbiotic relationship with nitrogen fixing bacteria. Consequently, even without being integrated into the lichen thallus, cyanobacteria associated with chlorolichens may be considered cyanobionts as well, i.e. being an integral part of the lichen symbiosis.

Besides the mycobiont, photobiont, and cyanobiont, there are other bacterial and fungal taxa that can impact lichens’ health, growth, and fitness^37^. For example, our functional prediction results showed some bacteria with multiple functions, essential to the lichen symbiotic system, such as those able to produce growth hormones, amino acids, or vitamins (e.g., *Luteibacter rhizovicinus*, *Variovorax boronicumulans*), displaying resistance to abiotic stressors (e.g., *Variovorax paradoxus, Hymenobacter actinosclerus, Ferruginibacter alkalilentus*), and playing role in nitrogen fixation and assimilation (e.g., *Prochlorothrix hollandica, Nitrosospira multiformis*). Other studies also corroborate the presence of lichen-associated bacteria providing these essential functions, thus contributing to lichen fitness^105–107^. As for the lichen mycobiome, the lichen species in this study were associated with a variety of fungal taxa with diverse functional attributes, including saprotrophy (e.g., *Vishniacozyma victoriae*, *Pterula gracilis*, *Filobasidium wieringae*), foliar endophytes (e.g., *Trichomerium* sp), pathogens and parasites (e.g., *Distocercospora pachyderma*, *Unguiculariopsis lettaui*, *Exophiala eucalyptigena*). This is consistent with previous studies showing multiple functions of lichen mycobiomes across the symbiotic spectrum, from commensalism and mutualism to parasitism and pathogenicity^90,108^.

In summary, our findings demonstrated that the urbanization gradient significantly impacts soil bacterial and fungal communities, with community structure, dynamics, and predicted functions differing between medium- to highly urbanized areas from those in less urbanized areas. The increasing influence of dispersal limitation and variable selection along this gradient resulted in higher community turnover and increased microbial interactions in the medium and high zones, especially in medium zone. In contrast, the urbanization gradient did not affect the microbiomes of the lichens *Candelaria*, *Xanthoria*, and *Physcia*. Instead, these lichen microbiomes exhibited high host specificity, highlighting species-specific symbiotic relationships, microbiome signatures, and predicted functions. Furthermore, the assembly of the lichen microbiome was governed by undominated processes, indicating that multiple ecological processes simultaneously influenced the assembly, with no single process being dominant. Given that urbanization introduces various environmental pressures across multiple scales, future studies should focus on how these factors interact with urban soil and lichen microbiomes. This approach would better disentangle which urban environmental factors drive microbial community variation and assess the potential of urban microbiomes as ecological indicators for specific environmental stressors.

## Methods

### Sample collection

Soils and lichen samples were collected in September 2023 across an urbanization gradient from the outskirts to the city center of Leiden, the Netherlands. The urbanization level was categorized based on the urban heat island (UHI) level as a proxy reflecting built-up area proportion. We defined three distinct zones, i.e., low urbanized (UHI 0‒0.8 °C temperature difference with the rural environment), medium urbanized (UHI 0.8‒1.6 °C), and highly urbanized (1.6‒2.5 °C). UHI values were derived from the UHI map of RIVM (Rijksinstituut voor Volksgezondheid en Milieu, 2020; www.atlasleefomgeving.nl). These zones are subsequently called low, medium, and high zone, respectively. A total of 93 soil samples were collected, encompassing 31 samples from each zone. The number of soil replicates per zone was chosen to balance logistical feasibility with sufficient statistical power able to capture spatial heterogeneity within each urbanization level. These soil samples were collected from a depth of 0–20 cm close to linden (*Tilia* sp.) trees. Additionally, lichen samples from the species *Candelaria concolor*, *Physcia adscendens*, and *Xanthoria parietina* (hereafter referred to as *Candelaria*, *Physcia*, and *Xanthoria*, respectively) were collected from the bark of the *Tilia* trees. All three lichen species are foliose chlorolichens that comprise a bipartite symbiosis, in which the ascomycete fungus (mycobiont), which defines the lichen’s identity, associates with unicellular green algae (phycobiont) enclosed within the lichen thallus. A total of 45 lichen samples were obtained, with 15 samples per species evenly distributed across the three zones (3 lichen species × 3 zones × 5 replicates). This sampling design ensured that each species was consistently represented along the urbanization gradient, allowing for comparative analyses while accounting for potential spatial variability. Both soil and lichen samples were stored at −20 °C for further analysis. A map of the sampling sites, including geographic coordinates and associated metadata, is provided in Supplementary Fig. 5a, b and Supplementary Table 6a, b.

### Total soil and lichen DNA extraction, nanopore sequencing, and raw sequence processing

Total DNA was extracted from soil samples using the DNeasy PowerLyzer PowerSoil Kit (Qiagen, Hilden, Germany) and from lichen samples using the Quick-DNA Fecal/Soil Microbe Miniprep Kit (Zymo Research, Freiburg, Germany), both following the manufacturer’s instructions. The lichen DNA extraction protocol was optimized by performing mechanical lysis by bead beating 3 times at 5500 rpm for 45 s. The extracted DNA concentration was measured using a Qubit dsDNA HS kit (Life Technologies Inc., Gaithersburg, MD, USA). To target bacterial communities, the full length of the bacterial 16S rRNA gene was PCR-amplified using the forward primer 16S-27F (5’-AGRGTTYGATYMTGGCTCAG-3’) and the reverse primer 16S-1492R (5’-ACCTTGTTACGACTT-3’)^109^. For fungal communities, the full length of the internal transcribed spacer 2 (ITS2) was amplified using the forward primer ITS3-F (5’-GCATCGATGAAGAACGCAGC-3’) and the reverse primer ITS4-R (5’-TCCTCCGCTTATTGATATGC-3’)^110^. The bacterial 16S rRNA and fungal ITS2 amplicons from each sample were purified using a NucleoMag NGS Clean-up and Size Selection Kit (Macherey-Nagel, Düren, Germany), following the manufacturer’s instructions. The fragment length of these amplicons was checked using QIAxcel (Qiagen, Hilden, Germany).

Prior to sequencing, these amplicons were indexed following the Nanopore Ligation Sequencing Amplicons -Native Barcoding Kit 96 V14 (SQK-NBD114.96) protocol. The amplicon concentration was determined using a Qubit dsDNA HS kit and diluted to 400 fmol (equivalent to 260 ng for ~2 kb amplicons), to be barcoded. The final ligation of Nanopore sequencing adapters was performed according to the SQK-NBD114.96 with EXP-PBC096 protocol. The concentration of the sequencing library was adjusted to 10–20 fmol in a volume of 12 µL, as recommended for loading onto R10.4.1 flow cells. These libraries were sequenced on two separate R10.4.1 GridION flow cells. The flow cells were loaded onto a GridION Mk-1 and sequenced for approximately 48 h until no additional sequencing reads could be obtained with a quality score above Q10. Fast5 files were basecalled and demultiplexed using the Guppy 6.4.2 high-accuracy model, and DNA sequence reads were generated as fastq files. Nanopore technology was used in this study to obtain long-read sequences, as we aimed for the full-length sequences of the bacterial 16S rRNA gene and fungal ITS2 region (the latter displaying high length heterogeneity in fungi). Although Nanopore has a relatively higher error rate compared to other short-read sequencing platforms^111^, the newest Oxford Nanopore R10.4.1 technology we used has been proven reliable in enabling accurate species-level assignments in microbial community profiling^112^.

The length and quality distribution of bacterial 16S rRNA and fungal ITS2 reads were visualized using NanoPlot and filtered with NanoFilt^113^ to discard reads with a quality score below Q10. The primer sequences from the filtered reads were removed using DADA2^114^. Sequences outside the 1.4–1.6 kb range for 16S rRNA and the 0.8–1.0 kb range for ITS2 were removed using the PRINSEQ sequence trimmer^115^. The taxonomic analyses were performed using Kraken2^116^ against the UNITE fungal database^117^ for ITS2 sequences and the NCBI bacterial database for 16S rRNA sequences. The feature table and taxonomic table were exported using kraken-biom^118^.

### Bacterial and fungal community analyses

The bacterial sequence data from soil and lichen samples were rarefied to the depths of 27,194 and 50,263 sequences per sample, respectively. For fungal communities, the data were rarefied to 68,236 and 111,227 sequences for soil and lichen samples, respectively. These different rarefaction depths were chosen to maximize data retention within each sample type while accounting for differences in reads quality, sequencing depth, and community complexity between soil and lichen samples. In the bacterial dataset, taxa identified as archaea, chloroplasts, mitochondria, and unassigned taxa were removed. In the fungal dataset, non-fungal and unassigned taxa were discarded. The remaining reads were used to calculate alpha diversity metrics, including species richness and the Shannon diversity index. Differences in bacterial and fungal community structure (beta diversity) were determined based on Bray Curtis dissimilarity and visualized via Principal Coordinates Analysis (PCoA). The assignment of bacterial and fungal functional guilds was determined using Faprotax^119^ and FungalTraits^120^, respectively. Previous studies have demonstrated that Faprotax and FungalTraits can successfully predict microbial guilds in urban and peri-urban environments^121,122^. However, it is important to acknowledge that (1) these databases have inherent limitations in trait and taxonomic information; and (2) variation in taxonomic resolution or taxonomic annotation can lead to inconsistencies or variations in functional guild annotations^123^. These analyses were carried out in R v.4.3.2 using the Vegan^124^ and Phyloseq^125^ packages.

Differences in alpha diversity parameters across the urbanization gradient and lichen species were analyzed using Kruskal–Wallis and Wilcoxon post hoc tests for non-parametric data, and ANOVA followed by post hoc Tukey tests for parametric data. The classification of data as parametric or non-parametric was determined using the Shapiro–Wilk test and Bartlett’s test, which evaluate data normality and homogeneity of variance, respectively. Differences in community beta diversity were analyzed using the Adonis permutation test with 10,000 permutations, followed by a post hoc pairwise Adonis test^126^.

### Differential abundance analysis

Taxa differential abundance analysis across the urbanization gradient was performed following a previously published pipeline (see refs. ^23,127^). In brief, important taxa were identified based on the following criteria: abundance >1%, presence in >10 samples, and significant (ANOVA, *p* value < 0.01) variation in relative abundance across different urbanization levels. The validity of the selected taxa was verified by comparing them to the original dataset using Procrustes analysis on Bray-Curtis dissimilarity matrices with the ‘protest‘ package^124^. Each data subset was then clustered based on taxa relative abundance across urbanization levels and visualized in a heatmap. Conversely, differentially abundant bacterial and fungal taxa across lichen species were identified using the DESeq2 package^128^. The obtained results were visualized using a circular heatmap and clustered based on log2 fold change values of each taxon.

### Microbial co-occurrence network analyses

Taxa co-occurrence including data from bacterial and fungal communities in soil and lichen samples were constructed using the Sparse Correlations for Compositional data (SparCC) algorithm^129^ in FastSpar v.1.0.0^130^. To reduce the influence of rare taxa, only those present in at least 3 samples with read counts higher than 20 were retained for the analysis. The degree and centrality of nodes in each network were quantified by a bootstrapping method with 10,000 iterations and were subsequently compared with the two-sample Kolmogorov–Smirnov test using the “ks.test” function. Only correlations with |r| > 0.9 for bacterial-bacterial and fungal-fungal interactions, and |r| > 0.8 for bacterial-fungal interactions with significant *p* values (*p* < 0.01) after Benjamini–Hochberg (BH) correction, were maintained for the final network. These threshold differences were chosen based on empirical observations in our dataset, where no bacterial-fungal correlations exceeded |r| > 0.9. This likely suggests that within-kingdom interactions tend to be stronger than cross-kingdom interactions, potentially reflecting ecological differences in association strength and functional dependencies. By setting these thresholds, we aimed to balance the retention of meaningful correlations (while minimizing spurious correlations) to ensure reliable network interpretations. The generated microbial network was visualized using Cytoscape v.3.10.2^131^ and analyzed using the Network Analyzer v.4.4.8 plugin^132^. Nodes with connections (node degree) > 4 were considered hubs in the final network.

### Bacterial and fungal community assembly processes

To quantify the relative contribution of distinct assembly processes structuring the soil and lichen microbiomes across the urbanization gradient and lichen species, we used a pre-established phylogenetic null modeling approach^21,22^. To improve the accuracy, the fungal phylogenetic tree used to calculate the assembly process from ITS2 sequences relied on a fungal phylogeny backbone based on 18S + 28S rRNA gene sequences (taxonomy_to_tree.pl script of Tedersoo et al.^133^. Meanwhile, phylogenetic tree for bacterial communities were built using FastTree (ver. 2.1.11)^134^.

Phylogenetic turnover was quantified using the β-nearest taxon index (βNTI), a metric that measures the deviation of observed phylogenetic turnover between pairs of communities from a null distribution. A detailed description of this metric is provided by Stegen et al.^22^. In brief, βNTI values significantly lower than −2 or greater than +2 indicates that selection is the dominant process structuring community assembly, as the phylogenetic turnover deviates from the null expectation. In addition, βNTI < −2 indicates homogeneous selection, a process that occurs when uniform abiotic and biotic conditions drive communities toward more similar structures. Whereas, a βNTI > +2 indicates heterogeneous/variable selection, a process that occurs when diverse abiotic and biotic conditions drive communities toward more dissimilar structures (see Dini-Andreote et al.^17^; Mawarda et al.^23^ for details). Last, βNTI values between −2 and +2 indicate no significant deviation from the null expectation and are thus interpreted as stochastic processes exerting the major role in community assembly.

Additionally, we used taxonomic turnover to further examine the low-level processes mediating stochasticity. This was done by quantifying the Raup-Crick metric (RCbray). Similarly to the phylogenetic turnover, we measured the deviation of taxonomic turnover between a pair of communities from the null distribution^22,135^. A detailed description of this metric is provided by Stegen et al.^22^. When combined with βNTI values, the RCbray metric helps identify other low-level processes influencing community assembly. That is, |βNTI| < 2 and RCbray >0.95 indicates dispersal limitation, a process that occurs when the movement or establishment/colonization of individuals in a new location is restricted, resulting in more dissimilar community structures;|βNTI| < 2 and RCbray < −0.95 indicates homogenizing dispersal, a process that occurs when a very high dispersal rate among communities reduces differences, leading to homogeneous community structures; and |βNTI| < 2 and |RCbray | < 0.95 accounts for ‘undominated processes,’ where selection, dispersal, diversification, and or drift jointly influence community assembly, but none of these processes is dominant. The relative influence of each assembly process was quantified by calculating its proportion within each soil and lichen sample across the urbanization gradient.

## Supplementary information

Supplementary Information

## Data Availability

Raw sequences were submitted to the NCBI Sequence Read Archive (SRA) and are available under the BioProject ID PRJNA1231394.

## Code availability

The code used in this study are provided in a public repository: 10.5281/zenodo.14965980.
