# Supplementary material for: Unveiling the ecological processes driving soil and lichen microbiome assembly along an urbanization gradient
Source: NPJ Biofilms Microbiomes. 2025 Jun 10;11:99. doi: 10.1038/s41522-025-00736-4 (PMC12152141; doi:10.1038/s41522-025-00736-4)
Supplement: Supplementary file 1 — Supplementary Information [file 41522_2025_736_MOESM1_ESM.pdf]

Figure captions

Supplementary Figure 1. Soil bacterial (a) and fungal (b) community richness across the urbanization gradient. Community turnover is depicted using bar plots based on Bray-Curtis distances for (c) soil bacterial community and (d) soil fungal community.

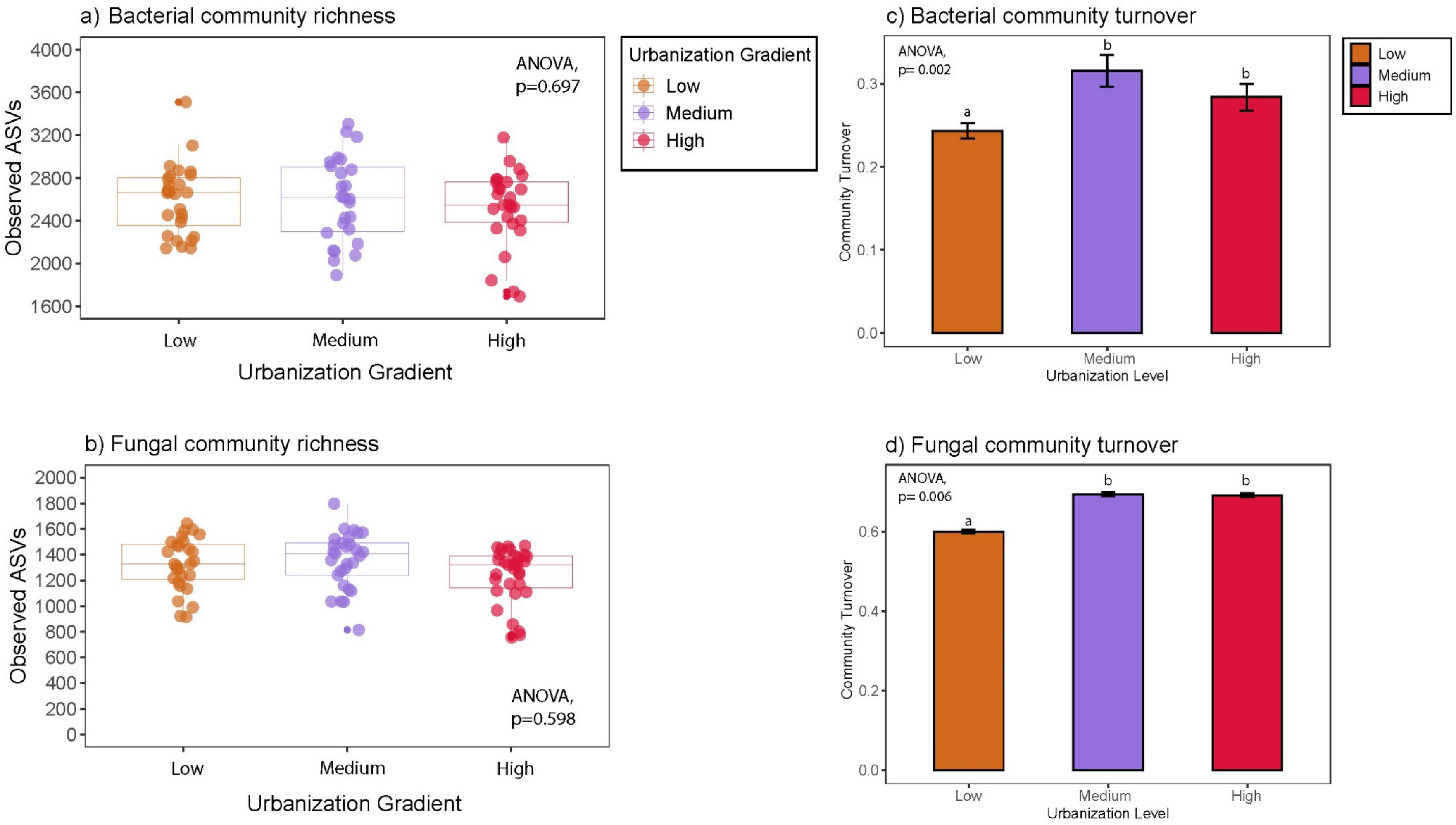

Supplementary Figure 2. Overview of lichen bacterial and fungal community diversity across the urbanization gradient in each lichen species. Alpha diversity is shown using the Shannon diversity index for (a) the lichen bacterial community and (b) the lichen fungal community. Community structure is depicted using PCoA plots based on Bray-Curtis distances for the lichen bacterial community in (c, d, e) and the lichen fungal community in (f, g, h) in each lichen species across low, medium, and highly urbanized areas, respectively.

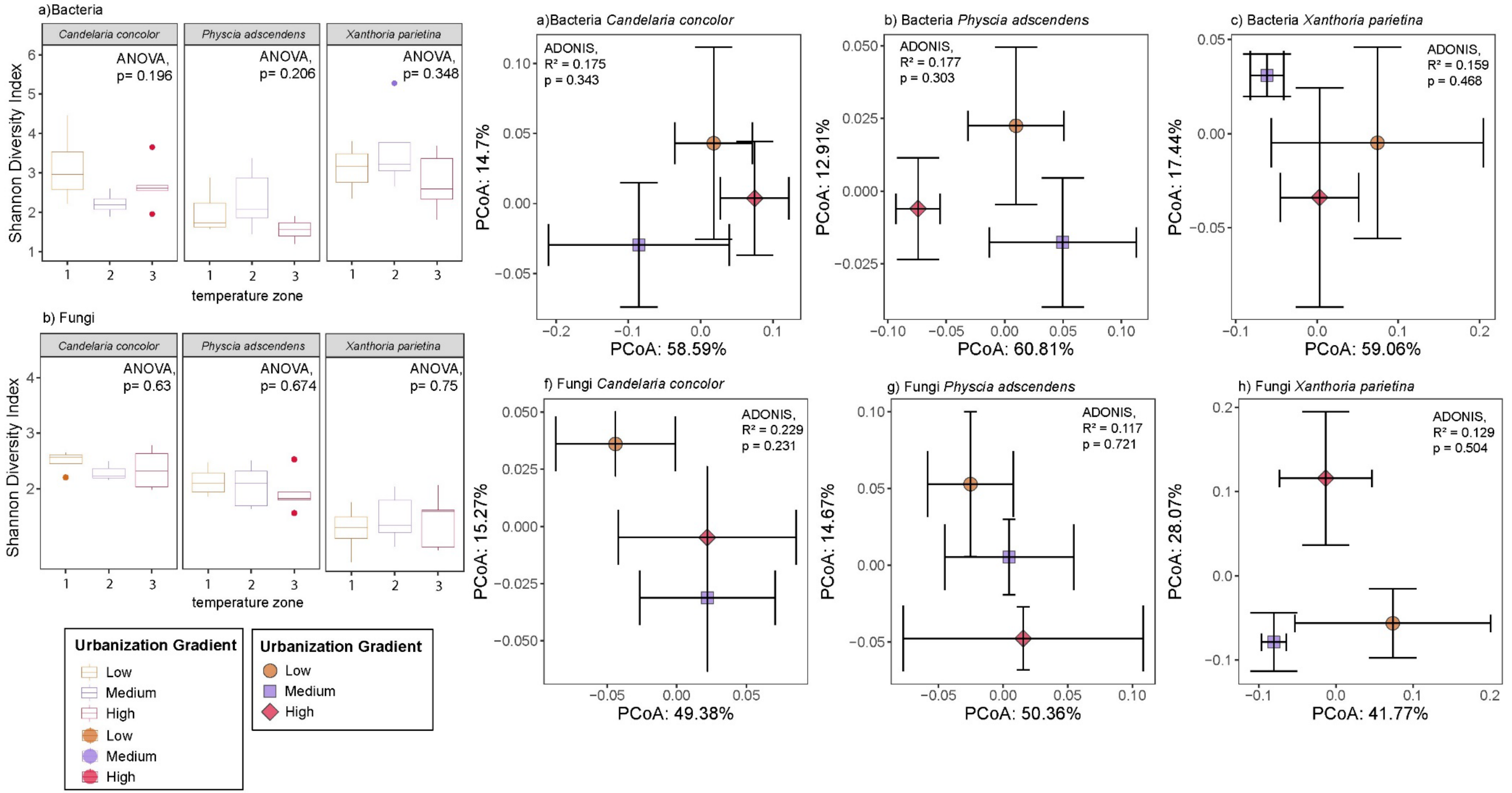

Supplementary Figure 3. Differential abundance lichen bacterial (a,b,c) and fungal communities (d,e,f) in Candelaria concolor, Physcia adscendens, and Xanthoria paritina respectively across urbanization gradient.

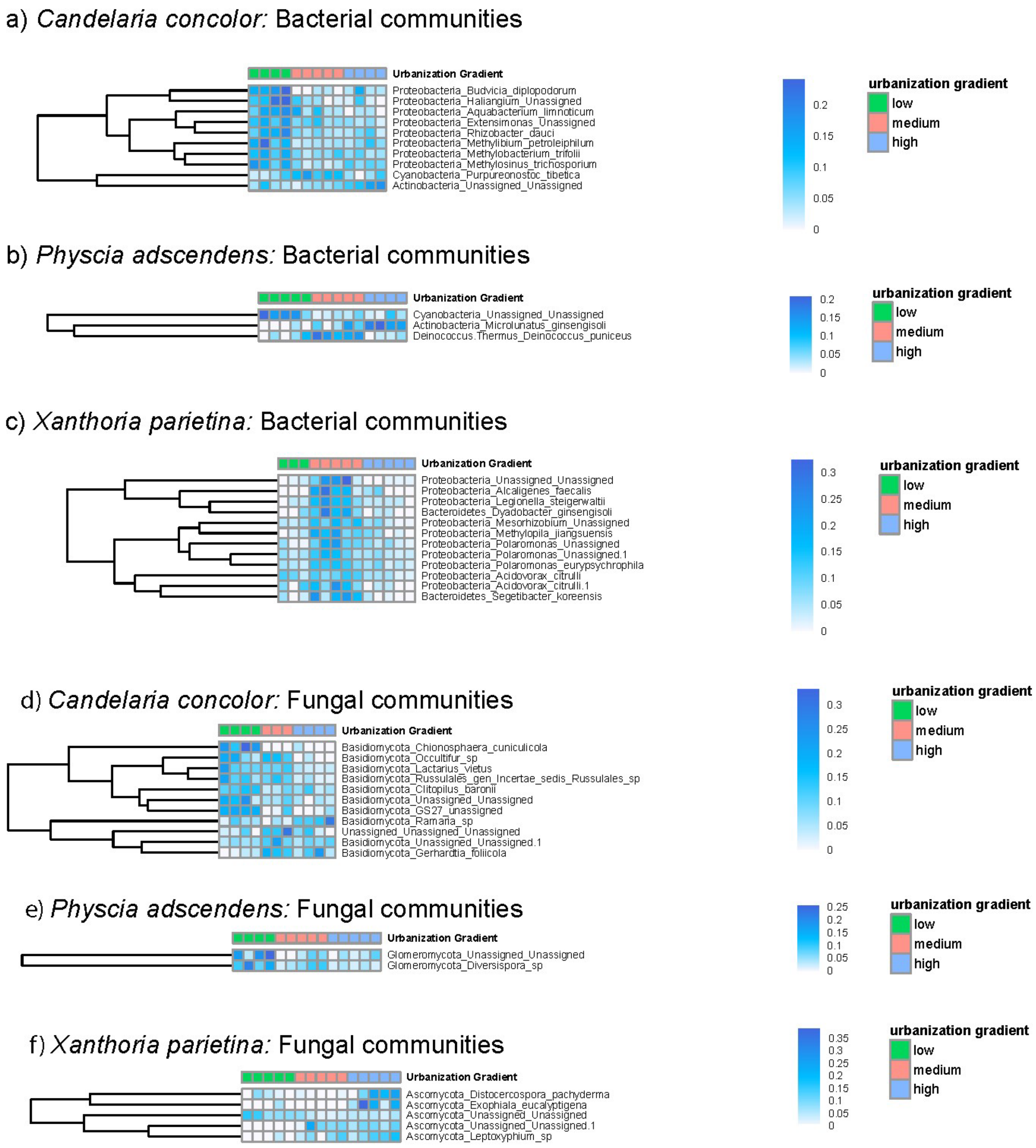

Supplementary Figure 4. Co-occurrence network between bacterial and fungal communities in (a) Candelaria concolor, (b) Physcia adscendens, and (c) Xanthoria parietina. Blue and red colors of the edges represent positive and negative relationships respectively, while node colors represent bacterial and fungal taxa. The shape of vertices reflects microbial domains (i.e., circle: bacteria; triangle: fungi), and the size of nodes refers to abundances.

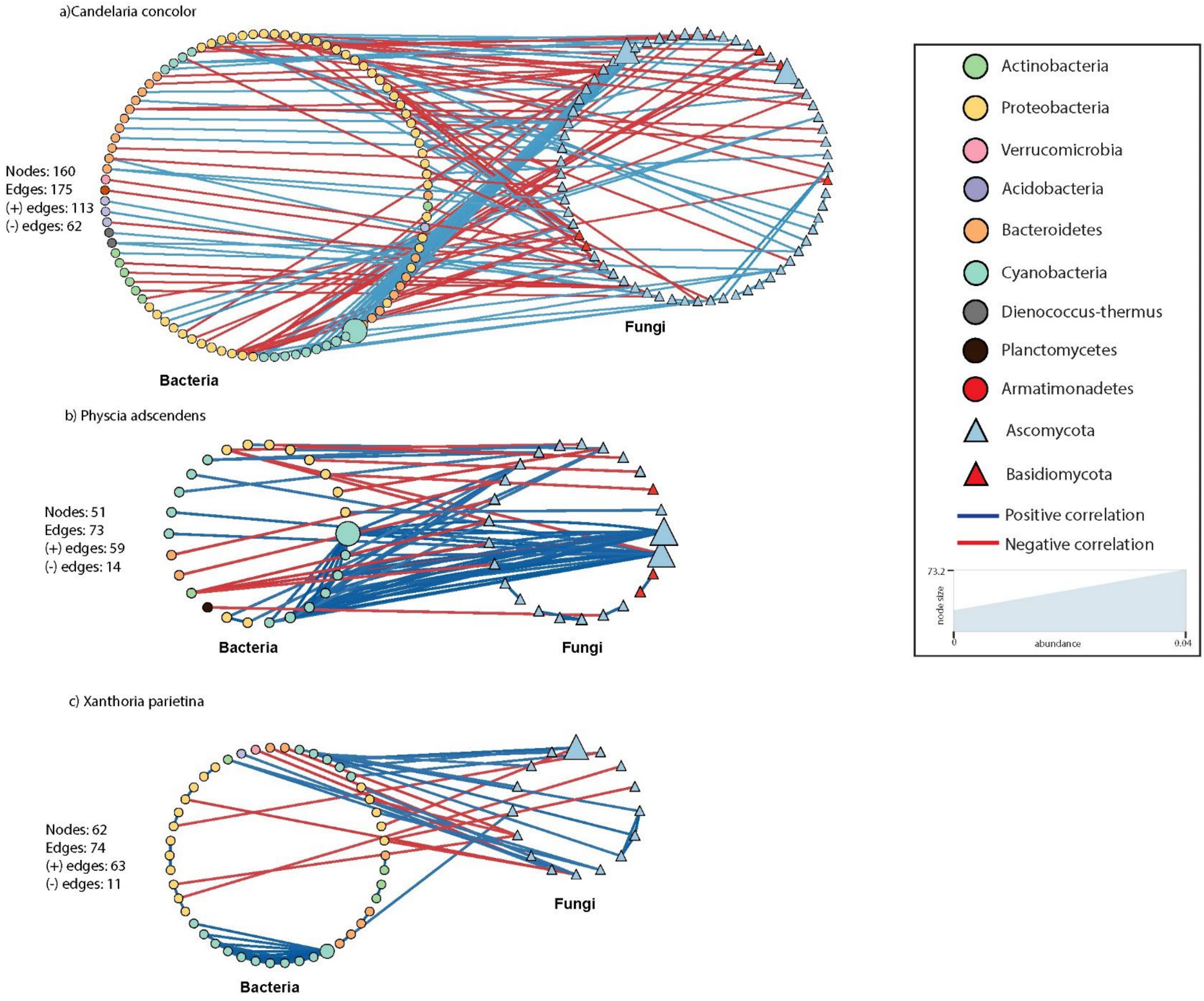

Supplementary Figure 5. Overview of the map where soil samples (a) and lichen samples were taken (b) across low, medium, and high urbanized area.

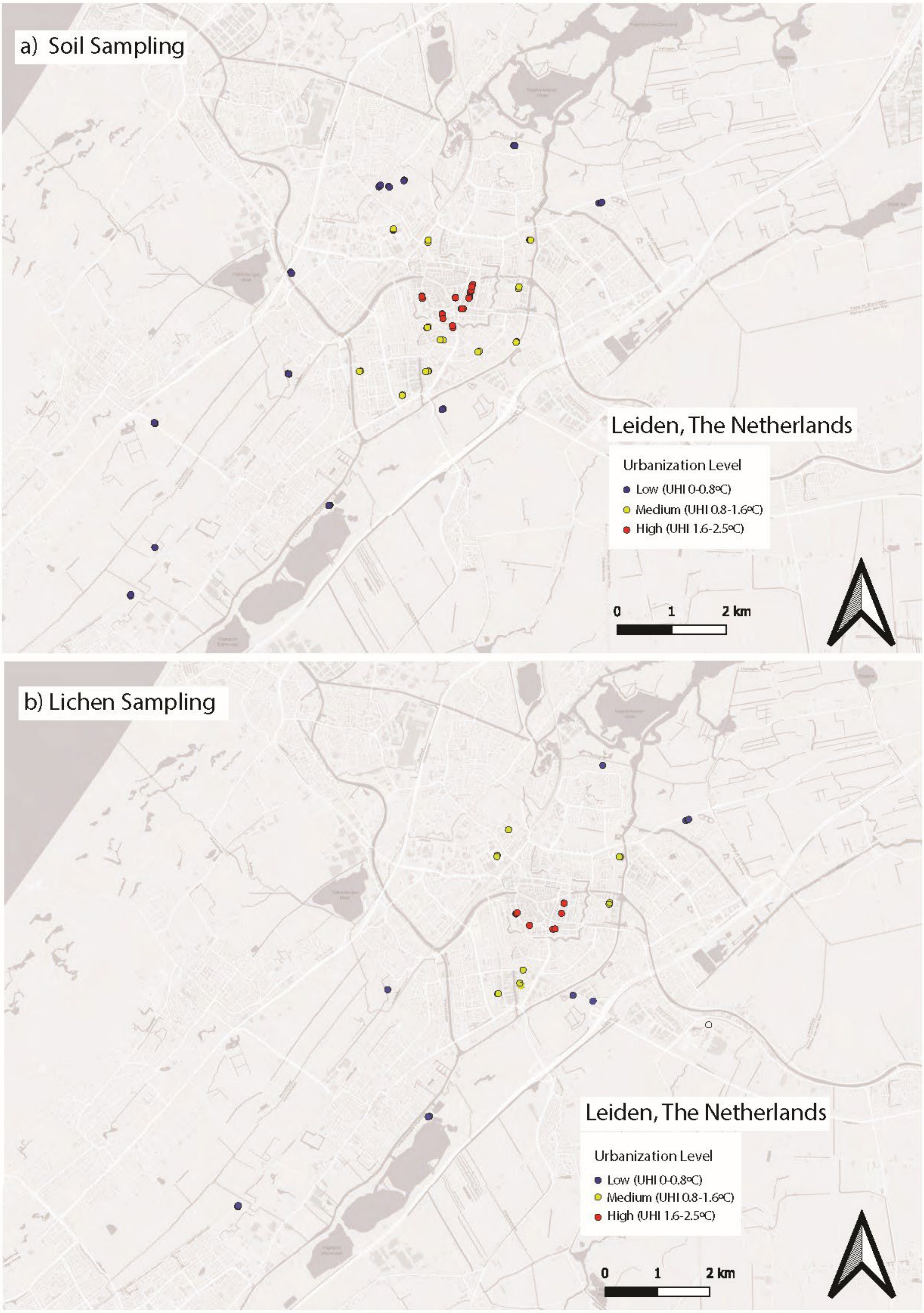

Supplementary Table 1a

No	Bacteria	Guild	Treatment	Remark
1	Acidovorax caeni	aerobic chemoheterotrophy_unknown	Group 1	Peaked its abundance in low urbanized area
2	Methylocystis rosea	methylotrophy_hydrocarbon degradation	Group 1	Peaked its abundance in low urbanized area
3	Georgfuchsia toluolica	aerobic chemoheterotrophy_denitrification	Group 1	Peaked its abundance in low urbanized area
4	Unassigned Unassigned	unknown_unknown	Group 1	Peaked its abundance in low urbanized area
5	Chelatococcus Unassigned	unknown_unknown	Group 1	Peaked its abundance in low urbanized area
6	Emticicia soli	aerobic chemoheterotrophy_denitrification	Group 1	Peaked its abundance in low urbanized area
7	Hyphomicrobium nitrativorans	aerobic chemoheterotrophy_denitrification	Group 1	Peaked its abundance in low urbanized area
8	Ferrovibrio xuzhouensis	aerobic chemoheterotrophy_xenobiotic degradation	Group 1	Peaked its abundance in low urbanized area
9	Methyloglobulus morosus	methanotrophy_methanol oxidation	Group 1	Peaked its abundance in low urbanized area
10	Dermabacter Unassigned	aerobic chemoheterotrophy_fermentation	Group 1	Peaked its abundance in low urbanized area
11	Propionivibrio limicola	chemoheterotrophy_fermentation	Group 1	Peaked its abundance in low urbanized area
12	Paludibacterium paludis	chemoheterotrophy_unknown	Group 1	Peaked its abundance in low urbanized area
13	Viridibacillus Unassigned	unknown_unknown	Group 1	Peaked its abundance in low urbanized area
14	Schlegelella Unassigned	unknown_unknown	Group 1	Peaked its abundance in low urbanized area
15	Hydrocarboniphaga daqingensis	chemoheterotrophy_hydrocarbon degradation	Group 1	Peaked its abundance in low urbanized area
16	Sutterella faecalis	chemoheterotrophy_unknown	Group 1	Peaked its abundance in low urbanized area
17	Pseudohaliea rubra	chemoheterotrophy_adipate degradation	Group 1	Peaked its abundance in low urbanized area
18	Nonomuraea Unassigned	aerobic chemoheterotrophy_unknown	Group 1	Peaked its abundance in low urbanized area
19	Turicimonas muris	chemoheterotrophy_ureolysis	Group 1	Peaked its abundance in low urbanized area
20	Uliginosibacterium sediminicola	aerobic chemoheterotrophy_unknown	Group 1	Peaked its abundance in low urbanized area
21	Bacillus cecembensis	aerobic chemoheterotrophy_unknown	Group 1	Peaked its abundance in low urbanized area
22	Mycobacterium aquaticum	aerobic chemoheterotrophy_unknown	Group 1	Peaked its abundance in low urbanized area
23	Thiobacillus Unassigned	unknown_unknown	Group 1	Peaked its abundance in low urbanized area
24	Paenibacillus lupini	aerobic chemoheterotrophy_unknown	Group 1	Peaked its abundance in low urbanized area
25	Marinosulfonomonas methylotropha	methylotrophy_unknown	Group 1	Peaked its abundance in low urbanized area
26	Paraburkholderia susongensis	aerobic chemoheterotrophy_unknown	Group 1	Peaked its abundance in low urbanized area
27	Pseudospirillum japonicum	aerobic chemoheterotrophy_unknown	Group 1	Peaked its abundance in low urbanized area
28	Thalassotalea Unassigned	aerobic chemoheterotrophy_unknown	Group 1	Peaked its abundance in low urbanized area
29	Telmatospirillum siberiense	chemoheterotrophy_nitrogen fixation	Group 1	Peaked its abundance in low urbanized area
30	Dongia rigui	unknown_unknown	Group 1	Peaked its abundance in low urbanized area
31	Dongia mobilis	unknown_unknown	Group 1	Peaked its abundance in low urbanized area
32	Lysobacter daejeonensis	chemoheterotrophy_chitinolysis	Group 1	Peaked its abundance in low urbanized area
33	Sinomonas echigonensis	unknown_unknown	Group 1	Peaked its abundance in low urbanized area
34	Liberibacter crescens	unknown_unknown	Group 1	Peaked its abundance in low urbanized area

35	Geoalkalibacter subterraneus	chemoheterotrophy_sulfur oxidation	Group 1	Peaked its abundance in low urbanized area
36	Unassigned Unassigned	unknown_unknown	Group 1	Peaked its abundance in low urbanized area
37	Acetobacter nitrogenifigens	aerobic chemoheterotrophy_unknown	Group 1	Peaked its abundance in low urbanized area
38	Nitrospirillum Unassigned	unknown_unknown	Group 1	Peaked its abundance in low urbanized area
39	Mycetohabitans Unassigned	unknown_unknown	Group 1	Peaked its abundance in low urbanized area
40	Ferruginivarius sediminum	unknown_unknown	Group 1	Peaked its abundance in low urbanized area
41	Caballeronia Unassigned	unknown_unknown	Group 1	Peaked its abundance in low urbanized area
42	Halomonas cerina	aerobic chemoheterotrophy_hydrocarbon degradation	Group 1	Peaked its abundance in low urbanized area
43	Melaminivora alkalimesophila	unknown_unknown	Group 1	Peaked its abundance in low urbanized area
44	Paenibacillus nebraskensis	aerobic chemoheterotrophy_unknown	Group 1	Peaked its abundance in low urbanized area
45	Paenibacillus aceris	aerobic chemoheterotrophy_unknown	Group 1	Peaked its abundance in low urbanized area
46	Bacillus mycoides	unknown_unknown	Group 1	Peaked its abundance in low urbanized area
47	Paenibacillus sacheonensis	aerobic chemoheterotrophy_unknown	Group 1	Peaked its abundance in low urbanized area
48	Nocardia Unassigned	aerobic chemoheterotrophy_unknown	Group 1	Peaked its abundance in low urbanized area
49	Bradyrhizobium retamae	aerobic chemoheterotrophy_nitrogen fixation	Group 1	Peaked its abundance in low urbanized area
50	Pseudolabrys taiwanensis	aerobic chemoheterotrophy_unknown	Group 1	Peaked its abundance in low urbanized area
51	Marinicauda Unassigned	unknown_unknown	Group 1	Peaked its abundance in low urbanized area
52	Unassigned Unassigned.4	unknown_unknown	Group 1	Peaked its abundance in low urbanized area
53	Paraburkholderia oxyphila	unknown_unknown	Group 1	Peaked its abundance in low urbanized area
54	Inmirania thermothiophila	unknown_unknown	Group 1	Peaked its abundance in low urbanized area
55	Methylacidimicrobium Unassigned	unknown_unknown	Group 1	Peaked its abundance in low urbanized area
56	Nitrosomonas Unassigned	aerobic chemoheterotrophy_nitrification	Group 1	Peaked its abundance in low urbanized area
57	Phycisphaera mikurensis	chemoheterotrophy_fermentation	Group 1	Peaked its abundance in low urbanized area
58	Coxiella Unassigned	animal parasites or symbionts_unknown	Group 1	Peaked its abundance in low urbanized area
59	Chromatocurvus halotolerans	unknown_unknown	Group 1	Peaked its abundance in low urbanized area
60	Methyloparacoccus murrellii	methanotrophy_hydrocarbon degradation	Group 1	Peaked its abundance in low urbanized area
61	Unassigned Unassigned.1	unknown_unknown	Group 1	Peaked its abundance in low urbanized area
62	Panacagrimonas perspica	unknown_unknown	Group 1	Peaked its abundance in low urbanized area
63	Unassigned Unassigned.2	unknown_unknown	Group 1	Peaked its abundance in low urbanized area
64	Reyranella Unassigned	unknown_unknown	Group 1	Peaked its abundance in low urbanized area
65	Unassigned Unassigned.3	unknown_unknown	Group 1	Peaked its abundance in low urbanized area
66	Alkanibacter difficilis	aerobic chemoheterotrophy_unknown	Group 1	Peaked its abundance in low urbanized area
67	Burkholderia australis	aerobic chemoheterotrophy_unknown	Group 1	Peaked its abundance in low urbanized area
68	Paucibacter oligotrophus	unknown_unknown	Group 1	Peaked its abundance in low urbanized area
69	Duodenibacillus massiliensis	unknown_unknown	Group 1	Peaked its abundance in low urbanized area
70	Minicystis rosea	unknown_unknown	Group 1	Peaked its abundance in low urbanized area
71	Unassigned Unassigned	unknown_unknown	Group 1	Peaked its abundance in low urbanized area

72	Methyloligella Unassigned	unknown_unknown	Group 1	Peaked its abundance in low urbanized area
73	Sutterella parvirubra	unknown_unknown	Group 1	Peaked its abundance in low urbanized area
74	Ohtaekwangia Unassigned	unknown_unknown	Group 1	Peaked its abundance in low urbanized area
75	Thermodesulforhabdus norvegica	chemoheterotrophy_sulfur oxidation	Group 1	Peaked its abundance in low urbanized area
76	Larsenimonas salina	aerobic chemoheterotrophy_unknown	Group 1	Peaked its abundance in low urbanized area
77	Marinomonas Unassigned	aerobic chemoheterotrophy_unknown	Group 1	Peaked its abundance in low urbanized area
78	Ornithinicoccus halotolerans	aerobic chemoheterotrophy_unknown	Group 1	Peaked its abundance in low urbanized area
79	Mesorhizobium albiziae	aerobic chemoheterotrophy_ureolysis	Group 1	Peaked its abundance in low urbanized area
80	Geminisphaera colitermitum	animal parasites or symbionts_unknown	Group 1	Peaked its abundance in low urbanized area
81	Euzebya tangerina	aerobic chemoheterotrophy_ureolysis	Group 1	Peaked its abundance in low urbanized area
82	Unassigned Unassigned	unknown_unknown	Group 1	Peaked its abundance in low urbanized area
83	Microbulbifer Unassigned	chemoheterotrophy_chitinolysis	Group 1	Peaked its abundance in low urbanized area
84	Phytomonospora Unassigned	unknown_unknown	Group 1	Peaked its abundance in low urbanized area
85	Acinetobacter colistiniresistens	aerobic chemoheterotrophy_hydrocarbon degradation	Group 2	Peaked its abundance in medium and highly urbanized area
86	Acinetobacter lwoffii	aerobic chemoheterotrophy_manganese oxidation	Group 2	Peaked its abundance in medium and highly urbanized area
87	Arsenicicoccus bolidensis	aerobic chemoheterotrophy_denitrification	Group 2	Peaked its abundance in medium and highly urbanized area
88	Raoultella electrica	unknown_unknown	Group 2	Peaked its abundance in medium and highly urbanized area
89	Mucilaginibacter calamicampi	aerobic chemoheterotrophy_unknown	Group 2	Peaked its abundance in medium and highly urbanized area
90	Paracoccus alkenifer	methylotrophy_methanol oxidation	Group 2	Peaked its abundance in medium and highly urbanized area
91	Blastococcus aggregatus	aerobic chemoheterotrophy_unknown	Group 2	Peaked its abundance in medium and highly urbanized area
92	Arthrobacter crystallopoietes	aerobic chemoheterotrophy_manganese oxidation	Group 2	Peaked its abundance in medium and highly urbanized area
93	Mycetocola manganoxydans	aerobic chemoheterotrophy_unknown	Group 2	Peaked its abundance in medium and highly urbanized area
94	Rubellimicrobium mesophilum	aerobic chemoheterotrophy_cellulolysis	Group 2	Peaked its abundance in medium and highly urbanized area
95	Microvirga soli	aerobic chemoheterotrophy_unknown	Group 2	Peaked its abundance in medium and highly urbanized area
96	Adhaeribacter swui	aerobic chemoheterotrophy_unknown	Group 2	Peaked its abundance in medium and highly urbanized area
97	Rhodocytophaga aerolata	unknown_unknown	Group 2	Peaked its abundance in medium and highly urbanized area
98	Roseateles terrae	unknown_unknown	Group 2	Peaked its abundance in medium and highly urbanized area
99	Flavobacterium notoginsengisoli	aerobic chemoheterotrophy_unknown	Group 2	Peaked its abundance in medium and highly urbanized area
100	Flavobacterium luteum	aerobic chemoheterotrophy_unknown	Group 2	Peaked its abundance in medium and highly urbanized area
101	Pseudorhodoferax Unassigned	unknown_unknown	Group 2	Peaked its abundance in medium and highly urbanized area
102	Pseudorhodoferax soli	aerobic chemoheterotrophy_unknown	Group 2	Peaked its abundance in medium and highly urbanized area
103	Brevundimonas aurantiaca	aerobic chemoheterotrophy_unknown	Group 2	Peaked its abundance in medium and highly urbanized area
104	Stenotrophomonas Unassigned	animal parasites or symbionts_human pathogens	Group 2	Peaked its abundance in medium and highly urbanized area
105	Acidovorax Unassigned	aerobic chemoheterotrophy_unknown	Group 2	Peaked its abundance in medium and highly urbanized area
106	Roseateles Unassigned	unknown_unknown	Group 2	Peaked its abundance in medium and highly urbanized area
107	Methylobacterium cerastii	methylotrophy_methanol oxidation	Group 2	Peaked its abundance in medium and highly urbanized area
108	Massilia brevitalea	unknown_unknown	Group 2	Peaked its abundance in medium and highly urbanized area

109	Massilia dura	unknown_unknown	Group 2	Peaked its abundance in medium and highly urbanized area
110	Inhella inkyongensis	aerobic chemoheterotrophy_denitrification	Group 2	Peaked its abundance in medium and highly urbanized area
111	Lysobacter oligotrophicus	chemoheterotrophy_chitinolysis	Group 2	Peaked its abundance in medium and highly urbanized area
112	Nitrosospira tenuis	aerobic chemoheterotrophy_nitrification	Group 2	Peaked its abundance in medium and highly urbanized area
113	Actimicrobium antarcticum	unknown_unknown	Group 2	Peaked its abundance in medium and highly urbanized area
114	Paenibacillus wynnii	aerobic chemoheterotrophy_denitrification	Group 2	Peaked its abundance in medium and highly urbanized area
115	Roseomonas oryzicola	animal parasites or symbionts_unknown	Group 2	Peaked its abundance in medium and highly urbanized area
116	Pseudomonas stutzeri	aerobic chemoheterotrophy_denitrification	Group 2	Peaked its abundance in medium and highly urbanized area
117	Pseudomonas alcaligenes	aerobic chemoheterotrophy_hydrocarbon degradation	Group 2	Peaked its abundance in medium and highly urbanized area
118	Pseudomonas nitritireducens	aerobic chemoheterotrophy_nitrification	Group 2	Peaked its abundance in medium and highly urbanized area
119	Sediminibacterium goheungense	unknown_unknown	Group 2	Peaked its abundance in medium and highly urbanized area
120	Hymenobacter rutilus	unknown_unknown	Group 2	Peaked its abundance in medium and highly urbanized area
121	Craurococcus roseus	aerobic chemoheterotrophy_nitrification	Group 2	Peaked its abundance in medium and highly urbanized area
122	Spirosoma areae	unknown_unknown	Group 2	Peaked its abundance in medium and highly urbanized area
123	Microlunatus flavus	aerobic chemoheterotrophy_unknown	Group 2	Peaked its abundance in medium and highly urbanized area
124	Roseomonas vinacea	animal parasites or symbionts_human pathogens	Group 2	Peaked its abundance in medium and highly urbanized area
125	Truepera radiovictrix	chemoheterotrophy_fermentation	Group 2	Peaked its abundance in medium and highly urbanized area
126	Massilia varians	unknown_unknown	Group 2	Peaked its abundance in medium and highly urbanized area
127	Deinococcus aquatilis	unknown_unknown	Group 2	Peaked its abundance in medium and highly urbanized area
128	Deinococcus humi	unknown_unknown	Group 2	Peaked its abundance in medium and highly urbanized area
129	Spirosoma arcticum	unknown_unknown	Group 2	Peaked its abundance in medium and highly urbanized area
130	Rubellimicrobium aerolatum	unknown_unknown	Group 2	Peaked its abundance in medium and highly urbanized area
131	Microlunatus aerolatus	aerobic chemoheterotrophy_unknown	Group 2	Peaked its abundance in medium and highly urbanized area
132	Massilia chloroacetimidivorans	unknown_unknown	Group 2	Peaked its abundance in medium and highly urbanized area
133	Methylobacterium goesingense	methylotrophy_methanol oxidation	Group 2	Peaked its abundance in medium and highly urbanized area
134	Aureimonas ferruginea	aerobic chemoheterotrophy_unknown	Group 2	Peaked its abundance in medium and highly urbanized area
135	Dyadobacter alkalitolerans	aerobic chemoheterotrophy_cellulolysis	Group 2	Peaked its abundance in medium and highly urbanized area
136	Massilia suwonensis	aerobic chemoheterotrophy_unknown	Group 2	Peaked its abundance in medium and highly urbanized area
137	Ilyomonas limi	unknown_unknown	Group 2	Peaked its abundance in medium and highly urbanized area
138	Larkinella knui	aerobic chemoheterotrophy_cellulolysis	Group 2	Peaked its abundance in medium and highly urbanized area
139	Devosia submarina	aerobic chemoheterotrophy_unknown	Group 2	Peaked its abundance in medium and highly urbanized area
140	Devosia yakushimensis	aerobic chemoheterotrophy_unknown	Group 2	Peaked its abundance in medium and highly urbanized area
141	Methylobacterium soli	methylotrophy_methanol oxidation	Group 2	Peaked its abundance in medium and highly urbanized area
142	Roseomonas ludipueritiae	animal parasites or symbionts_human pathogens	Group 2	Peaked its abundance in medium and highly urbanized area
143	Methylorubrum Unassigned	unknown_unknown	Group 2	Peaked its abundance in medium and highly urbanized area
144	Microvirga guangxiensis	aerobic chemoheterotrophy_unknown	Group 2	Peaked its abundance in medium and highly urbanized area
145	Enterovirga rhinocerotis	animal parasites or symbionts_unknown	Group 2	Peaked its abundance in medium and highly urbanized area

146	Dankookia rubra	unknown_unknown	Group 2	Peaked its abundance in medium and highly urbanized area
147	Microvirga subterranea	aerobic chemoheterotrophy_denitrification	Group 2	Peaked its abundance in medium and highly urbanized area
148	Skermanella aerolata	aerobic chemoheterotrophy_unknown	Group 2	Peaked its abundance in medium and highly urbanized area
149	Lysobacter spongiae	chemoheterotrophy_chitinolysis	Group 2	Peaked its abundance in medium and highly urbanized area
150	Noviherbaspirillum psychrotolerans	chemoheterotrophy_unknown	Group 2	Peaked its abundance in medium and highly urbanized area
151	Arthrobacter rhombi	aerobic chemoheterotrophy_ureolysis	Group 2	Peaked its abundance in medium and highly urbanized area
152	Noviherbaspirillum canariense	aerobic chemoheterotrophy_unknown	Group 2	Peaked its abundance in medium and highly urbanized area
153	Noviherbaspirillum aurantiacum	aerobic chemoheterotrophy_unknown	Group 2	Peaked its abundance in medium and highly urbanized area
154	Noviherbaspirillum Unassigned	unknown_unknown	Group 2	Peaked its abundance in medium and highly urbanized area
155	Segetibacter koreensis	aerobic chemoheterotrophy_unknown	Group 2	Peaked its abundance in medium and highly urbanized area
156	Flavisolibacter metallilatus	aerobic chemoheterotrophy_metalloid resistant	Group 2	Peaked its abundance in medium and highly urbanized area
157	Flavisolibacter ginsenosidimutans	aerobic chemoheterotrophy_denitrification	Group 2	Peaked its abundance in medium and highly urbanized area
158	Chloroflexus Unassigned	unknown_unknown	Group 2	Peaked its abundance in medium and highly urbanized area
159	Micromonospora Unassigned	aerobic chemoheterotrophy_unknown	Group 2	Peaked its abundance in medium and highly urbanized area
160	Roseomonas arctica	animal parasites or symbionts_unknown	Group 2	Peaked its abundance in medium and highly urbanized area
161	Phycicoccus jejuensis	aerobic chemoheterotrophy_unknown	Group 2	Peaked its abundance in medium and highly urbanized area
162	Phycicoccus elongatus	aerobic chemoheterotrophy_unknown	Group 2	Peaked its abundance in medium and highly urbanized area
163	Tetrasphaera veronensis	aerobic chemoheterotrophy_unknown	Group 2	Peaked its abundance in medium and highly urbanized area
164	Neobacillus vireti	chemoheterotrophy_adipate degradation	Group 3	Peaked its abundance only in highly urbanized area
165	Paracoccus koreensis	methylotrophy_methanol oxidation	Group 3	Peaked its abundance only in highly urbanized area
166	Acidimangrovimonas sediminis	anaerobic chemoheterotrophy_unknown	Group 3	Peaked its abundance only in highly urbanized area
167	Cumulibacter manganitolerans	aerobic chemoheterotrophy_manganese oxidation	Group 3	Peaked its abundance only in highly urbanized area
168	Calothrix desertica	oxygenic photoautotrophy_nitrogen fixation	Group 3	Peaked its abundance only in highly urbanized area
169	Paludisphaera borealis	aerobic chemoheterotrophy_adipate degradation	Group 3	Peaked its abundance only in highly urbanized area
170	Lederbergia panacisoli	aerobic chemoheterotrophy_unknown	Group 3	Peaked its abundance only in highly urbanized area
171	Sporosarcina luteola	aerobic chemoheterotrophy_denitrification	Group 3	Peaked its abundance only in highly urbanized area
172	Sporosarcina ureae	chemoheterotrophy_unknown	Group 3	Peaked its abundance only in highly urbanized area
173	Sporosarcina newyorkensis	animal parasites or symbionts_unknown	Group 3	Peaked its abundance only in highly urbanized area
174	Aurantiacibacter atlanticus	aerobic chemoheterotrophy_unknown	Group 3	Peaked its abundance only in highly urbanized area
175	Roseovarius antarcticus	animal parasites or symbionts_unknown	Group 3	Peaked its abundance only in highly urbanized area
176	Virgibacillus zhanjiangensis	aerobic chemoheterotrophy_unknown	Group 3	Peaked its abundance only in highly urbanized area
177	Piscibacillus Unassigned	unknown_unknown	Group 3	Peaked its abundance only in highly urbanized area
178	Tumebacillus permanentifrigoris	aerobic chemoheterotrophy_sulfur oxidation	Group 3	Peaked its abundance only in highly urbanized area
179	Halobacillus Unassigned	unknown_unknown	Group 3	Peaked its abundance only in highly urbanized area
180	Kaistia soli	aerobic chemoheterotrophy_fermentation	Group 3	Peaked its abundance only in highly urbanized area

Supplementary Table 1b

No	Phylum	Genus	Species	Guild	Group	Remark
1	Ascomycota	Arthroderma	crocatum	animal_parasite	Group1	Peaked its abundance only in low urbanized area
2	Ascomycota	Pseudosigmoidea	sp	soil_saprotroph	Group1	Peaked its abundance only in low urbanized area
3	Ascomycota	Unassigned	Unassigned	Unknown	Group1	Peaked its abundance only in low urbanized area
4	Ascomycota	Oidiodendron	eucalypti	soil_saprotroph	Group1	Peaked its abundance only in low urbanized area
5	Ascomycota	Cordyceps	farinosa	animal_parasite	Group1	Peaked its abundance only in low urbanized area
6	Basidiomycota	GS27	ord	Unknown	Group1	Peaked its abundance only in low urbanized area
7	Ascomycota	Keithomyces	Unassigned	Unknown	Group1	Peaked its abundance only in low urbanized area
8	Ascomycota	Pararoussoella	sp	litter_saprotroph	Group1	Peaked its abundance only in low urbanized area
9	Ascomycota	Neooccultibambusa	sp	wood_saprotroph	Group1	Peaked its abundance only in low urbanized area
10	Ascomycota	Calycina	Unassigned	wood_saprotroph	Group1	Peaked its abundance only in low urbanized area
11	Ascomycota	Ciboria	coryli	plant_pathogen	Group1	Peaked its abundance only in low urbanized area
12	Ascomycota	Pochonia	sp	animal_parasite	Group1	Peaked its abundance only in low urbanized area
13	Basidiomycota	Mutinus	sp	soil_saprotroph	Group1	Peaked its abundance only in low urbanized area
14	Basidiomycota	Oliveoniaceae	sp	Unknown	Group1	Peaked its abundance only in low urbanized area
15	Ascomycota	Valsaria	Unassigned	wood_saprotroph	Group1	Peaked its abundance only in low urbanized area
16	Basidiomycota	Armillaria	borealis	litter_saprotroph	Group1	Peaked its abundance only in low urbanized area
17	Ascomycota	Minimelanolocus	sp	wood_saprotroph	Group1	Peaked its abundance only in low urbanized area
18	Ascomycota	Beauveria	Unassigned	animal_parasite	Group1	Peaked its abundance only in low urbanized area
19	Ascomycota	Amphichorda	sp	dung_saprotroph	Group1	Peaked its abundance only in low urbanized area
20	Ascomycota	Unassigned	Unassigned	Unknown	Group1	Peaked its abundance only in low urbanized area
21	Ascomycota	Metapochonia	Unassigned	animal_parasite	Group1	Peaked its abundance only in low urbanized area
22	Zoopagomycota	Syncephalis	sp	mycoparasite	Group1	Peaked its abundance only in low urbanized area
23	Ascomycota	Keithomyces	carneus	Unknown	Group1	Peaked its abundance only in low urbanized area
24	Ascomycota	Clavicipitaceae	sp	Unknown	Group1	Peaked its abundance only in low urbanized area
25	Ascomycota	Pleotrichocladium	opacum	wood_saprotroph	Group1	Peaked its abundance only in low urbanized area
26	Ascomycota	Clonostachys	candelabrum	wood_saprotroph	Group1	Peaked its abundance only in low urbanized area
27	Ascomycota	Humicola	Unassigned	wood_saprotroph	Group1	Peaked its abundance only in low urbanized area
28	Ascomycota	Teratosphaeriaceae	sp	Unknown	Group1	Peaked its abundance only in low urbanized area
29	Ascomycota	Keithomyces	sp	Unknown	Group1	Peaked its abundance only in low urbanized area
30	Mortierellomycota	Podila	humilis	Unknown	Group1	Peaked its abundance only in low urbanized area
31	Ascomycota	Volutella	sp	plant_pathogen	Group1	Peaked its abundance only in low urbanized area
32	Ascomycota	Penicillium	scabrosum	unspecified_saprotroph	Group1	Peaked its abundance only in low urbanized area
33	Ascomycota	Penicillium	angulare	unspecified_saprotroph	Group1	Peaked its abundance only in low urbanized area
34	Ascomycota	Unassigned	Unassigned.1	Unknown	Group1	Peaked its abundance only in low urbanized area

35	Ascomycota	Penicillium	christenseniae	unspecified_saprotroph	Group1	Peaked its abundance only in low urbanized area
36	Ascomycota	Lophiotrema	sp	wood_saprotroph	Group1	Peaked its abundance only in low urbanized area
37	Basidiomycota	Ganoderma	adspersum	plant_pathogen	Group1	Peaked its abundance only in low urbanized area
38	Ascomycota	Penicillium	glabrum	unspecified_saprotroph	Group1	Peaked its abundance only in low urbanized area
39	Ascomycota	Helotiales	sp	Unknown	Group1	Peaked its abundance only in low urbanized area
40	Ascomycota	Geosmithia	sp	unspecified_saprotroph	Group1	Peaked its abundance only in low urbanized area
41	Ascomycota	Trichothecium	sympodiale	plant_pathogen	Group1	Peaked its abundance only in low urbanized area
42	Basidiobolomycota	Basidiobolomycota	sp	Unknown	Group1	Peaked its abundance only in low urbanized area
43	Olpidiomycota	Olpidiomycota	sp	Unknown	Group1	Peaked its abundance only in low urbanized area
44	Ascomycota	Metarhizium	sp	animal_parasite	Group1	Peaked its abundance only in low urbanized area
45	Ascomycota	Penicillium	camponoti	unspecified_saprotroph	Group1	Peaked its abundance only in low urbanized area
46	Ascomycota	Plenodomus	sp	plant_pathogen	Group1	Peaked its abundance only in low urbanized area
47	Ascomycota	Pyrenopeziza	atrata	plant_pathogen	Group1	Peaked its abundance only in low urbanized area
48	Ascomycota	Myrmecridium	sp	soil_saprotroph	Group1	Peaked its abundance only in low urbanized area
49	Basidiomycota	Unassigned	Unassigned	Unknown	Group1	Peaked its abundance only in low urbanized area
50	Ascomycota	Achroceratosphaeria	sp	wood_saprotroph	Group1	Peaked its abundance only in low urbanized area
51	Ascomycota	Unassigned	Unassigned.2	Unknown	Group1	Peaked its abundance only in low urbanized area
52	Ascomycota	Myrmecridium	Unassigned	soil_saprotroph	Group1	Peaked its abundance only in low urbanized area
53	Ascomycota	Myrmecridium	schulzeri	soil_saprotroph	Group1	Peaked its abundance only in low urbanized area
54	Ascomycota	Amphisphaeriales	sp	Unknown	Group1	Peaked its abundance only in low urbanized area
55	Basidiomycota	Ganoderma	applanatum	plant_pathogen	Group1	Peaked its abundance only in low urbanized area
56	Ascomycota	Dactylonectria	Unassigned	plant_pathogen	Group1	Peaked its abundance only in low urbanized area
57	Ascomycota	Dactylonectria	macrodidyma	plant_pathogen	Group1	Peaked its abundance only in low urbanized area
58	Ascomycota	Ilyonectria	destructans	plant_pathogen	Group1	Peaked its abundance only in low urbanized area
59	Basidiomycota	Agaricomycetes	sp	Unknown	Group1	Peaked its abundance only in low urbanized area
60	Ascomycota	Ilyonectria	Unassigned	plant_pathogen	Group1	Peaked its abundance only in low urbanized area
61	Ascomycota	Bionectria	Unassigned	wood_saprotroph	Group1	Peaked its abundance only in low urbanized area
62	Ascomycota	Trichothecium	crotocinigenum	plant_pathogen	Group1	Peaked its abundance only in low urbanized area
63	Ascomycota	Aspergillus	alliaceus	unspecified_saprotroph	Group1	Peaked its abundance only in low urbanized area
64	Ascomycota	Beauveria	sp	animal_parasite	Group1	Peaked its abundance only in low urbanized area
65	Ascomycota	Cephalotrichum	microsporum	soil_saprotroph	Group1	Peaked its abundance only in low urbanized area
66	Mucoromycota	Absidia	sp	soil_saprotroph	Group1	Peaked its abundance only in low urbanized area
67	Ascomycota	Paracosmospora	physciae	Unknown	Group2	Peaked its abundance only in medium and highly urbanized area
68	Ascomycota	Scolecopeltidium	menglaense	epiphyte	Group2	Peaked its abundance only in medium and highly urbanized area
69	Basidiomycota	Solicoccozyma	Unassigned	soil_saprotroph	Group2	Peaked its abundance only in medium and highly urbanized area
70	Basidiomycota	Solicoccozyma	gelidoterrea	soil_saprotroph	Group2	Peaked its abundance only in medium and highly urbanized area
71	Basidiomycota	Solicoccozyma	sp	soil_saprotroph	Group2	Peaked its abundance only in medium and highly urbanized area

72	Glomeromycota	Archaeosporaceae	sp	Unknown	Group2	Peaked its abundance only in medium and highly urbanized area
73	Basidiomycota	Puccinia	recondita	plant_pathogen	Group2	Peaked its abundance only in medium and highly urbanized area
74	Ascomycota	Unassigned	Unassigned.8	Unknown	Group2	Peaked its abundance only in medium and highly urbanized area
75	Basidiomycota	Termitomyces	sp	litter_saprotroph	Group2	Peaked its abundance only in medium and highly urbanized area
76	Ascomycota	Pyricularia	pennisetigena	plant_pathogen	Group2	Peaked its abundance only in medium and highly urbanized area
77	Ascomycota	Paraphoma	sp	plant_pathogen	Group2	Peaked its abundance only in medium and highly urbanized area
78	Ascomycota	Penidiella	sp	plant_pathogen	Group2	Peaked its abundance only in medium and highly urbanized area
79	Basidiomycota	Sporobolomyces	roseus	mycoparasite	Group2	Peaked its abundance only in medium and highly urbanized area
80	Ascomycota	Sarcinomyces	sp	foliar_endophyte	Group2	Peaked its abundance only in medium and highly urbanized area
81	Basidiomycota	Vishniacozyma	victoriae	soil_saprotroph	Group2	Peaked its abundance only in medium and highly urbanized area
82	Ascomycota	Acarospora	sp	lichenized	Group2	Peaked its abundance only in medium and highly urbanized area
83	Ascomycota	Aureobasidium	melanogenum	sooty_mold	Group2	Peaked its abundance only in medium and highly urbanized area
84	Ascomycota	Aureobasidium	sp	sooty_mold	Group2	Peaked its abundance only in medium and highly urbanized area
85	Ascomycota	Unassigned	Unassigned.9	Unknown	Group2	Peaked its abundance only in medium and highly urbanized area
86	Ascomycota	Aureobasidium	Unassigned	sooty_mold	Group2	Peaked its abundance only in medium and highly urbanized area
87	Ascomycota	Aureobasidium	pullulans	sooty_mold	Group2	Peaked its abundance only in medium and highly urbanized area
88	Ascomycota	Unassigned	Unassigned.6	Unknown	Group2	Peaked its abundance only in medium and highly urbanized area
89	Basidiomycota	Microstroma	bacarum	plant_pathogen	Group2	Peaked its abundance only in medium and highly urbanized area
90	Ascomycota	Acarospora	stapfiana	lichenized	Group2	Peaked its abundance only in medium and highly urbanized area
91	Ascomycota	Aureobasidium	proteae	sooty_mold	Group2	Peaked its abundance only in medium and highly urbanized area
92	Ascomycota	Pseudosydowia	sp	plant_pathogen	Group2	Peaked its abundance only in medium and highly urbanized area
93	Ascomycota	Erioscyphella	sasibrevispora	litter_saprotroph	Group2	Peaked its abundance only in medium and highly urbanized area
94	Ascomycota	Neophaeomoniella	constricta	plant_pathogen	Group2	Peaked its abundance only in medium and highly urbanized area
95	Basidiomycota	Pluteus	plautus	litter_saprotroph	Group2	Peaked its abundance only in medium and highly urbanized area
96	Basidiomycota	Unassigned	Unassigned.1	Unknown	Group2	Peaked its abundance only in medium and highly urbanized area
97	Basidiomycota	Unassigned	Unassigned.2	Unknown	Group2	Peaked its abundance only in medium and highly urbanized area
98	Basidiomycota	Occultifur	sp	mycoparasite	Group2	Peaked its abundance only in medium and highly urbanized area
99	Ascomycota	Penicillium	castellonense	unspecified_saprotroph	Group2	Peaked its abundance only in medium and highly urbanized area
100	Ascomycota	Alternaria	atra	plant_pathogen	Group2	Peaked its abundance only in medium and highly urbanized area
101	Ascomycota	Pseudocercospora	musae	plant_pathogen	Group2	Peaked its abundance only in medium and highly urbanized area
102	Ascomycota	Unassigned	Unassigned.7	Unknown	Group2	Peaked its abundance only in medium and highly urbanized area
103	Ascomycota	Neocosmospora	falciformis	soil_saprotroph	Group2	Peaked its abundance only in medium and highly urbanized area
104	Ascomycota	Achaetomium	lippiae	unspecified_saprotroph	Group2	Peaked its abundance only in medium and highly urbanized area
105	Ascomycota	Neopyrenochaeta	chromolaenae	unspecified_saprotroph	Group2	Peaked its abundance only in medium and highly urbanized area
106	Ascomycota	Lepraria	santosii	lichenized	Group2	Peaked its abundance only in medium and highly urbanized area
107	Ascomycota	Setophaeosphaeria	sp	plant_pathogen	Group2	Peaked its abundance only in medium and highly urbanized area
108	Ascomycota	Dothidotthia	symphoricarpi	plant_pathogen	Group2	Peaked its abundance only in medium and highly urbanized area

109	Ascomycota	Exserohilum	turcicum	plant_pathogen	Group2	Peaked its abundance only in medium and highly urbanized area
110	Ascomycota	Didymellaceae	sp	Unknown	Group2	Peaked its abundance only in medium and highly urbanized area
111	Ascomycota	Neocamarosporium	betae	wood_saprotroph	Group2	Peaked its abundance only in medium and highly urbanized area
112	Ascomycota	Ampelomyces	quisqualis	plant_pathogen	Group2	Peaked its abundance only in medium and highly urbanized area
113	Ascomycota	Unassigned	Unassigned.5	Unknown	Group2	Peaked its abundance only in medium and highly urbanized area
114	Ascomycota	Phaeosphaeria	pontiformis	litter_saprotroph	Group2	Peaked its abundance only in medium and highly urbanized area
115	Ascomycota	Alternaria	radicina	plant_pathogen	Group2	Peaked its abundance only in medium and highly urbanized area
116	Ascomycota	Pleosporaceae	sp	Unknown	Group2	Peaked its abundance only in medium and highly urbanized area
117	Ascomycota	Petrophila	sp	unspecified_saprotroph	Group2	Peaked its abundance only in medium and highly urbanized area
118	Ascomycota	Tubeufiaceae	sp	Unknown	Group2	Peaked its abundance only in medium and highly urbanized area
119	Ascomycota	Mytilinidion	sp	wood_saprotroph	Group2	Peaked its abundance only in medium and highly urbanized area
120	Ascomycota	Curvularia	senegalensis	plant_pathogen	Group2	Peaked its abundance only in medium and highly urbanized area
121	Ascomycota	Banhegyia	sp	wood_saprotroph	Group2	Peaked its abundance only in medium and highly urbanized area
122	Ascomycota	Golovinomyces	cichoracearum	plant_pathogen	Group2	Peaked its abundance only in medium and highly urbanized area
123	Basidiomycota	Papiliotrema	Unassigned	mycoparasite	Group2	Peaked its abundance only in medium and highly urbanized area
124	Ascomycota	Unassigned	Unassigned.10	Unknown	Group2	Peaked its abundance only in medium and highly urbanized area
125	Ascomycota	Otidea	cantharella	ectomycorrhizal	Group2	Peaked its abundance only in medium and highly urbanized area
126	Ascomycota	Peziza	echinospora	soil_saprotroph	Group2	Peaked its abundance only in medium and highly urbanized area
127	Ascomycota	Unassigned	Unassigned	Unknown	Group2	Peaked its abundance only in medium and highly urbanized area
128	Ascomycota	Hypomontagnella	monticulosa	wood_saprotroph	Group2	Peaked its abundance only in medium and highly urbanized area
129	Ascomycota	Morchella	sp	soil_saprotroph	Group2	Peaked its abundance only in medium and highly urbanized area
130	Ascomycota	Botryosphaeria	dothidea	plant_pathogen	Group2	Peaked its abundance only in medium and highly urbanized area
131	Ascomycota	Fusarium	iranicum	plant_pathogen	Group2	Peaked its abundance only in medium and highly urbanized area
132	Ascomycota	Colletotrichum	sp	plant_pathogen	Group2	Peaked its abundance only in medium and highly urbanized area
133	Ascomycota	Comoclathris	sp	wood_saprotroph	Group2	Peaked its abundance only in medium and highly urbanized area
134	Ascomycota	Neoscirrhia	matteucciicola	Unknown	Group2	Peaked its abundance only in medium and highly urbanized area
135	Ascomycota	Phoma	sp	plant_pathogen	Group2	Peaked its abundance only in medium and highly urbanized area
136	Ascomycota	Drechslera	sp	plant_pathogen	Group2	Peaked its abundance only in medium and highly urbanized area
137	Ascomycota	Fusarium	solani	plant_pathogen	Group2	Peaked its abundance only in medium and highly urbanized area
138	Ascomycota	Epicoccum	sorghinum	plant_pathogen	Group2	Peaked its abundance only in medium and highly urbanized area
139	Ascomycota	Epicoccum	sp	plant_pathogen	Group2	Peaked its abundance only in medium and highly urbanized area
140	Ascomycota	Ectophoma	multirostrata	soil_saprotroph	Group2	Peaked its abundance only in medium and highly urbanized area
141	Ascomycota	Karstenia	sp	unspecified_saprotroph	Group3	Peaked its abundance only in highly urbanized area
142	Ascomycota	Parmelia	ernstiae	lichenized	Group3	Peaked its abundance only in highly urbanized area
143	Ascomycota	Aspergillus	flocculosus	unspecified_saprotroph	Group3	Peaked its abundance only in highly urbanized area
144	Ascomycota	Kiliasia	athallina	lichenized	Group3	Peaked its abundance only in highly urbanized area
145	Basidiomycota	Leucocoprinus	sp	soil_saprotroph	Group3	Peaked its abundance only in highly urbanized area

146	Basidiomycota	Trichosporon	sp	animal_parasite	Group3	Peaked its abundance only in highly urbanized area
147	Mucoromycota	Mucor	Unassigned	soil_saprotroph	Group3	Peaked its abundance only in highly urbanized area
148	Basidiomycota	Cutaneotrichosporon	cutaneum	animal_parasite	Group3	Peaked its abundance only in highly urbanized area
149	Ascomycota	Petriella	sp	dung_saprotroph	Group3	Peaked its abundance only in highly urbanized area
150	Mucoromycota	Mucor	circinelloides	soil_saprotroph	Group3	Peaked its abundance only in highly urbanized area
151	Ascomycota	Debaryomyces	hansenii	nectar/tap_saprotroph	Group3	Peaked its abundance only in highly urbanized area

Supplementary Table 2a

No	Name	log2fold_change	guild	guild2	code	remark	guildfunction	target_microbe
1	Rhizobium azooxidifex	-5.07989	chemoheterotrophy	nitrification	CP	Higher Candelaria - Physcia	chemoheterotrophy nitrification	Lichen Bacteria
2	Cytophaga hutchinsonii	-4.97093	aerobic chemoheterotrophy	cellulolysis	CP	Higher Candelaria - Physcia	aerobic chemoheterotrophy cellulolysis	Lichen Bacteria
3	Chryseolinea serpens	-4.75613	aerobic chemoheterotrophy	xylanolysis	CP	Higher Candelaria - Physcia	aerobic chemoheterotrophy xylanolysis	Lichen Bacteria
4	Hymenobacter lapidarius	-4.41913	aerobic chemoheterotrophy	unknown	CP	Higher Candelaria - Physcia	aerobic chemoheterotrophy unknown	Lichen Bacteria
5	Mesorhizobium soli	-4.33812	aerobic chemoheterotrophy	ureolysis	CP	Higher Candelaria - Physcia	aerobic chemoheterotrophy ureolysis	Lichen Bacteria
6	Mucilaginibacter pineti	-4.18425	aerobic chemoheterotrophy	cellulolysis	CP	Higher Candelaria - Physcia	aerobic chemoheterotrophy cellulolysis	Lichen Bacteria
7	Mucilaginibacter dorajii	-4.14455	aerobic chemoheterotrophy	unknown	CP	Higher Candelaria - Physcia	aerobic chemoheterotrophy unknown	Lichen Bacteria
8	Variovorax boronicumulans	-3.8193	aerobic chemoheterotrophy	plant growth promotion	CP	Higher Candelaria - Physcia	aerobic chemoheterotrophy plant growth promotion	Lichen Bacteria
9	Luteibacter rhizovicinus	-3.81104	aerobic chemoheterotrophy	plant growth promotion	CP	Higher Candelaria - Physcia	aerobic chemoheterotrophy plant growth promotion	Lichen Bacteria
10	Mucilaginibacter craterilacus	-3.75516	aerobic chemoheterotrophy	unknown	CP	Higher Candelaria - Physcia	aerobic chemoheterotrophy unknown	Lichen Bacteria
11	Cellvibrio japonicus	-3.69273	aerobic chemoheterotrophy	cellulolysis	CP	Higher Candelaria - Physcia	aerobic chemoheterotrophy cellulolysis	Lichen Bacteria
12	Devosia chinhatensis	-3.64651	aerobic chemoheterotrophy	hydrocarbon degradation	CP	Higher Candelaria - Physcia	aerobic chemoheterotrophy hydrocarbon degradation	Lichen Bacteria
13	Solirubrobacter taibaiensis	-3.56365	aerobic chemoheterotrophy	unknown	CP	Higher Candelaria - Physcia	aerobic chemoheterotrophy unknown	Lichen Bacteria
14	Mucilaginibacter pedocola	-3.54073	aerobic chemoheterotrophy	cellulolysis	CP	Higher Candelaria - Physcia	aerobic chemoheterotrophy cellulolysis	Lichen Bacteria

15	Cellvibrio gandavensis	-3.44559	unknown	unknown	CP	Higher Candelaria - Physcia	unknown unknown	Lichen Bacteria
16	Pedobacter changchengzhani	-3.43403	aerobic chemoheterotrophy	unknown	CP	Higher Candelaria - Physcia	aerobic chemoheterotrophy unknown	Lichen Bacteria
17	Mucilaginibacter endophyticus	-3.43364	unknown	unknown	CP	Higher Candelaria - Physcia	unknown unknown	Lichen Bacteria
18	Flavobacterium cerinum	-3.41159	aerobic chemoheterotrophy	unknown	CP	Higher Candelaria - Physcia	aerobic chemoheterotrophy unknown	Lichen Bacteria
19	Reyranella terrae	-3.37074	aerobic chemoheterotrophy	unknown	CP	Higher Candelaria - Physcia	aerobic chemoheterotrophy unknown	Lichen Bacteria
20	Thermasporomyces composti	-3.29762	unknown	unknown	CP	Higher Candelaria - Physcia	unknown unknown	Lichen Bacteria
21	Polyangium sorediatum	-3.28941	aerobic chemoheterotrophy	unknown	CP	Higher Candelaria - Physcia	aerobic chemoheterotrophy unknown	Lichen Bacteria
22	Alicycliphilus denitrificans	-3.26384	chemoheterotrophy	denitrification	CP	Higher Candelaria - Physcia	chemoheterotrophy denitrification	Lichen Bacteria
23	Jahnella thaxteri	-3.24241	aerobic chemoheterotrophy	unknown	CP	Higher Candelaria - Physcia	aerobic chemoheterotrophy unknown	Lichen Bacteria
24	Ramlibacter solisilvae	-3.19593	aerobic chemoheterotrophy	unknown	CP	Higher Candelaria - Physcia	aerobic chemoheterotrophy unknown	Lichen Bacteria
25	Acidibacter ferrireducens	-3.11613	extremophilic	iron reduction	CP	Higher Candelaria - Physcia	extremophilic iron reduction	Lichen Bacteria
26	Rhizobium sp	-3.07809	unknown	unknown	CP	Higher Candelaria - Physcia	unknown unknown	Lichen Bacteria
27	Ferruginibacter alkalilentus	-2.91456	aerobic chemoheterotrophy	xenobiotics degradation	CP	Higher Candelaria - Physcia	aerobic chemoheterotrophy xenobiotics degradation	Lichen Bacteria
28	Compostibacter hankyongensis	-2.8894	aerobic chemoheterotrophy	unknown	CP	Higher Candelaria - Physcia	aerobic chemoheterotrophy unknown	Lichen Bacteria
29	Cellvibrio sp	-2.86807	unknown	unknown	CP	Higher Candelaria - Physcia	unknown unknown	Lichen Bacteria
30	Polaromonas vacuolata	-2.81248	chemoheterotrophy	ureolysis	CP	Higher Candelaria - Physcia	chemoheterotrophy ureolysis	Lichen Bacteria
31	Microcystis aeruginosa	-2.7772	oxygenic photoautotrophy	toxin producing cyanobacteria	CP	Higher Candelaria - Physcia	oxygenic photoautotrophy toxin producing cyanobacteria	Lichen Bacteria
32	Parviterribacter kavangonensis	-2.74087	aerobic chemoheterotrophy	unknown	CP	Higher Candelaria - Physcia	aerobic chemoheterotrophy unknown	Lichen Bacteria
33	Comamonas denitrificans	-2.66959	chemoheterotrophy	denitrification	CP	Higher Candelaria - Physcia	chemoheterotrophy denitrification	Lichen Bacteria
34	Algoriphagus aquaemixtae	-2.66062	aerobic chemoheterotrophy	unknown	CP	Higher Candelaria - Physcia	aerobic chemoheterotrophy unknown	Lichen Bacteria

35	Variovorax paradoxus	-2.51661	chemoheterotrophy	arsenic detoxification	CP	Higher Candelaria - Physcia	chemoheterotrophy arsenic detoxification	Lichen Bacteria
36	Thermus Deinococcus	-2.50227	extremophilic	unknown	CP	Higher Candelaria - Physcia	extremophilic unknown	Lichen Bacteria
37	Microvirga rosea	-2.48258	aerobic chemoheterotrophy	unknown	CP	Higher Candelaria - Physcia	aerobic chemoheterotrophy unknown	Lichen Bacteria
38	Aetherobacter sp	-2.46767	unknown	unknown	CP	Higher Candelaria - Physcia	unknown unknown	Lichen Bacteria
39	Acidovorax sp	-2.35288	unknown	unknown	CP	Higher Candelaria - Physcia	unknown unknown	Lichen Bacteria
40	Mucilaginibacter hankyongensis	-2.26295	aerobic chemoheterotrophy	unknown	CP	Higher Candelaria - Physcia	aerobic chemoheterotrophy unknown	Lichen Bacteria
41	Mucilaginibacter panaciglaebae	-1.61599	aerobic chemoheterotrophy	unknown	CP	Higher Candelaria - Physcia	aerobic chemoheterotrophy unknown	Lichen Bacteria
42	Mucilaginibacter sp	-1.58442	unknown	unknown	CP	Higher Candelaria - Physcia	unknown unknown	Lichen Bacteria
43	Cyanobacteria Unassigned	0.71938	oxygenic photoautotrophy	unknown	PC	Higher Physcia - Candelaria	oxygenic photoautotrophy unknown	Lichen Bacteria
44	Methylobacterium pseudosasicola	1.354421	chemoheterotrophy	methanol oxidation	PC	Higher Physcia - Candelaria	chemoheterotrophy methanol oxidation	Lichen Bacteria
45	Methylobacterium persicinum	1.516283	chemoheterotrophy	methanol oxidation	PC	Higher Physcia - Candelaria	chemoheterotrophy methanol oxidation	Lichen Bacteria
46	Pseudomonas alcaligenes	2.311588	aerobic chemoheterotrophy	hydrocarbon degradation	PC	Higher Physcia - Candelaria	aerobic chemoheterotrophy hydrocarbon degradation	Lichen Bacteria
47	Sphingomonas polyaromaticivorans	2.919573	aerobic chemoheterotrophy	hydrocarbon degradation	PC	Higher Physcia - Candelaria	aerobic chemoheterotrophy hydrocarbon degradation	Lichen Bacteria
48	Aureimonas frigidaquae	2.993637	aerobic chemoheterotrophy	denitrification	PC	Higher Physcia - Candelaria	aerobic chemoheterotrophy denitrification	Lichen Bacteria
49	Cyanobacteria Unassigned	3.201983	oxygenic photoautotrophy	unknown	PC	Higher Physcia - Candelaria	oxygenic photoautotrophy unknown	Lichen Bacteria
50	Clostridium algifaecis	4.428511	chemoheterotrophy	unknown	PC	Higher Physcia - Candelaria	chemoheterotrophy unknown	Lichen Bacteria
51	Pseudomonas bohemica	4.66907	aerobic chemoheterotrophy	unknown	PC	Higher Physcia - Candelaria	aerobic chemoheterotrophy unknown	Lichen Bacteria
52	Haloleptolyngbya elongata	-4.15486	oxygenic photoautotrophy	unknown	CX	Higher Candelaria - Xanthoria	oxygenic photoautotrophy unknown	Lichen Bacteria
53	Hymenobacter lapidarius	-3.12833	aerobic chemoheterotrophy	unknown	CX	Higher Candelaria - Xanthoria	aerobic chemoheterotrophy unknown	Lichen Bacteria
54	Cyanobacteria Unassigned	-2.98802	oxygenic photoautotrophy	unknown	CX	Higher Candelaria - Xanthoria	oxygenic photoautotrophy unknown	Lichen Bacteria

55	Leptodesmis alaskaensis	-2.93685	oxygenic photoautotrophy	unknown	CX	Higher Candelaria - Xanthoria	oxygenic photoautotrophy unknown	Lichen Bacteria
56	Aegeococcus thureti	-2.90594	oxygenic photoautotrophy	unknown	CX	Higher Candelaria - Xanthoria	oxygenic photoautotrophy unknown	Lichen Bacteria
57	Alicycliphilus denitrificans	-2.83405	chemoheterotrophy	denitrification	CX	Higher Candelaria - Xanthoria	chemoheterotrophy denitrification	Lichen Bacteria
58	Proteobacteria Unassigned	-2.70225	unknown	unknown	CX	Higher Candelaria - Xanthoria	unknown unknown	Lichen Bacteria
59	Comamonas denitrificans	-2.62399	chemoheterotrophy	denitrification	CX	Higher Candelaria - Xanthoria	chemoheterotrophy denitrification	Lichen Bacteria
60	Thermus Deinococcus	-2.54333	extremophilic	unknown	CX	Higher Candelaria - Xanthoria	extremophilic unknown	Lichen Bacteria
61	Altericista violacea	-2.46907	oxygenic photoautotrophy	unknown	CX	Higher Candelaria - Xanthoria	oxygenic photoautotrophy unknown	Lichen Bacteria
62	Solirubrobacter taibaiensis	-1.98144	aerobic chemoheterotrophy	unknown	CX	Higher Candelaria - Xanthoria	aerobic chemoheterotrophy unknown	Lichen Bacteria
63	Cyanobacteria Unassigned	-1.9307	oxygenic photoautotrophy	unknown	CX	Higher Candelaria - Xanthoria	oxygenic photoautotrophy unknown	Lichen Bacteria
64	Mucilaginibacter sp	1.052378	unknown	unknown	XC	Higher Xanthoria - Candelaria	unknown unknown	Lichen Bacteria
65	Microcystis aeruginosa	1.335928	oxygenic photoautotrophy	toxin producing cyanobacteria	XC	Higher Xanthoria - Candelaria	oxygenic photoautotrophy toxin producing cyanobacteria	Lichen Bacteria
66	Polyangium sorediatum	1.620839	aerobic chemoheterotrophy	unknown	XC	Higher Xanthoria - Candelaria	aerobic chemoheterotrophy unknown	Lichen Bacteria
67	Devosia psychrophila	2.079031	aerobic chemoheterotrophy	unknown	XC	Higher Xanthoria - Candelaria	aerobic chemoheterotrophy unknown	Lichen Bacteria
68	Cellvibrio sp	2.240917	unknown	unknown	XC	Higher Xanthoria - Candelaria	unknown unknown	Lichen Bacteria
69	Nostoc oromo	2.566233	oxygenic photoautotrophy	nitrogen fixation	XC	Higher Xanthoria - Candelaria	oxygenic photoautotrophy nitrogen fixation	Lichen Bacteria
70	Rhizobium gei	2.595558	aerobic chemoheterotrophy	unknown	XC	Higher Xanthoria - Candelaria	aerobic chemoheterotrophy unknown	Lichen Bacteria
71	Nodularia spumigena	2.645446	oxygenic photoautotrophy	unknown	XC	Higher Xanthoria - Candelaria	oxygenic photoautotrophy unknown	Lichen Bacteria
72	Sphingomonas polyaromaticivorans	2.649442	aerobic chemoheterotrophy	hydrocarbon degradation	XC	Higher Xanthoria - Candelaria	aerobic chemoheterotrophy hydrocarbon degradation	Lichen Bacteria
73	Pseudomonas alcaligenes	2.72106	aerobic chemoheterotrophy	hydrocarbon degradation	XC	Higher Xanthoria - Candelaria	aerobic chemoheterotrophy hydrocarbon degradation	Lichen Bacteria
74	Prochlorothrix hollandica	2.771192	oxygenic photoautotrophy	nitrogen fixation	XC	Higher Xanthoria - Candelaria	oxygenic photoautotrophy nitrogen fixation	Lichen Bacteria

75	Mucilaginibacter mallensis	2.845517	aerobic chemoheterotrophy	adipate degradation	XC	Higher Xanthoria - Candelaria	aerobic chemoheterotrophy adipate degradation	Lichen Bacteria
76	Streptomyces polyantibioticus	2.893551	aerobic chemoheterotrophy	unknown	XC	Higher Xanthoria - Candelaria	aerobic chemoheterotrophy unknown	Lichen Bacteria
77	Luteolibacter pohnpeiensis	2.914643	aerobic chemoheterotrophy	unknown	XC	Higher Xanthoria - Candelaria	aerobic chemoheterotrophy unknown	Lichen Bacteria
78	Aureimonas frigidaquae	2.985537	aerobic chemoheterotrophy	denitrification	XC	Higher Xanthoria - Candelaria	aerobic chemoheterotrophy denitrification	Lichen Bacteria
79	Simplicispira metamorpha	3.01386	aerobic chemoheterotrophy	denitrification	XC	Higher Xanthoria - Candelaria	aerobic chemoheterotrophy denitrification	Lichen Bacteria
80	Cellvibrio zantedeschiae	3.049221	aerobic chemoheterotrophy	unknown	XC	Higher Xanthoria - Candelaria	aerobic chemoheterotrophy unknown	Lichen Bacteria
81	Kallipyga massiliensis	3.149408	animal parasite or symbiones	unknown	XC	Higher Xanthoria - Candelaria	animal parasite or symbiones unknown	Lichen Bacteria
82	Methylobacterium pseudosasicola	3.157114	chemoheterotrophy	methanol oxidation	XC	Higher Xanthoria - Candelaria	chemoheterotrophy methanol oxidation	Lichen Bacteria
83	Methylobacterium persicinum	3.228631	chemoheterotrophy	methanol oxidation	XC	Higher Xanthoria - Candelaria	chemoheterotrophy methanol oxidation	Lichen Bacteria
84	Herbiconiux moechotypicola	3.260079	aerobic chemoheterotrophy	xylanolysis	XC	Higher Xanthoria - Candelaria	aerobic chemoheterotrophy xylanolysis	Lichen Bacteria
85	Methylobacterium bullatum	3.283085	aerobic chemoheterotrophy	unknown	XC	Higher Xanthoria - Candelaria	aerobic chemoheterotrophy unknown	Lichen Bacteria
86	Microbacterium aurum	3.301134	aerobic chemoheterotrophy	fermentation	XC	Higher Xanthoria - Candelaria	aerobic chemoheterotrophy fermentation	Lichen Bacteria
87	Pedobacter ginsengiterrae	3.381817	aerobic chemoheterotrophy	unknown	XC	Higher Xanthoria - Candelaria	aerobic chemoheterotrophy unknown	Lichen Bacteria
88	Luteolibacter gellanilyticus	3.542922	aerobic chemoheterotrophy	unknown	XC	Higher Xanthoria - Candelaria	aerobic chemoheterotrophy unknown	Lichen Bacteria
89	Sphingomonas qilianensis	3.720564	aerobic chemoheterotrophy	unknown	XC	Higher Xanthoria - Candelaria	aerobic chemoheterotrophy unknown	Lichen Bacteria
90	Nitrosospira multiformis	3.898159	chemoautotrophy	nitrification	XC	Higher Xanthoria - Candelaria	chemoautotrophy nitrification	Lichen Bacteria
91	Hymenobacter actinosclerus	4.100776	aerobic chemoheterotrophy	hydrocarbon degradation	XC	Higher Xanthoria - Candelaria	aerobic chemoheterotrophy hydrocarbon degradation	Lichen Bacteria
92	Agreia sp	4.235077	unknown	unknown	XC	Higher Xanthoria - Candelaria	unknown unknown	Lichen Bacteria
93	Cyanobacteria Unassigned	4.569271	oxygenic photoautotrophy	unknown	XC	Higher Xanthoria - Candelaria	oxygenic photoautotrophy unknown	Lichen Bacteria
94	Noviherbaspirillum soli	4.799155	aerobic chemoheterotrophy	unknown	XC	Higher Xanthoria - Candelaria	aerobic chemoheterotrophy unknown	Lichen Bacteria

95	Chitinimonas koreensis	4.922568	aerobic chemoheterotrophy	unknown	XC	Higher Xanthoria - Candelaria	aerobic chemoheterotrophy unknown	Lichen Bacteria
96	Pseudomonas bohemica	6.006303	aerobic chemoheterotrophy	unknown	XC	Higher Xanthoria - Candelaria	aerobic chemoheterotrophy unknown	Lichen Bacteria
97	Gloeothece membranacea	8.579619	oxygenic photoautotrophy	unknown	XC	Higher Xanthoria - Candelaria	oxygenic photoautotrophy unknown	Lichen Bacteria
98	Cyanobacteria Unassigned	-4.61192	oxygenic photoautotrophy	unknown	PX	Higher Physcia - Xanthoria	oxygenic photoautotrophy unknown	Lichen Bacteria
99	Haloleptolyngbya elongata	-4.53297	oxygenic photoautotrophy	unknown	PX	Higher Physcia - Xanthoria	oxygenic photoautotrophy unknown	Lichen Bacteria
100	Leptodesmis alaskaensis	-3.45083	oxygenic photoautotrophy	unknown	PX	Higher Physcia - Xanthoria	oxygenic photoautotrophy unknown	Lichen Bacteria
101	Aegeococcus thureti	-3.399	oxygenic photoautotrophy	unknown	PX	Higher Physcia - Xanthoria	oxygenic photoautotrophy unknown	Lichen Bacteria
102	Altericista violacea	-3.36256	oxygenic photoautotrophy	unknown	PX	Higher Physcia - Xanthoria	oxygenic photoautotrophy unknown	Lichen Bacteria
103	Proteobacteria Unassigned	-3.3032	unknown	unknown	PX	Higher Physcia - Xanthoria	unknown unknown	Lichen Bacteria
104	Cyanobacteria Unassigned	-2.80865	oxygenic photoautotrophy	unknown	PX	Higher Physcia - Xanthoria	oxygenic photoautotrophy unknown	Lichen Bacteria
105	Clostridium algifaecis	-2.76632	chemoheterotrophy	unknown	PX	Higher Physcia - Xanthoria	chemoheterotrophy unknown	Lichen Bacteria
106	Cyanobacteria Unassigned	-2.71349	oxygenic photoautotrophy	unknown	PX	Higher Physcia - Xanthoria	oxygenic photoautotrophy unknown	Lichen Bacteria
107	Solirubrobacter taibaiensis	1.606684	aerobic chemoheterotrophy	unknown	XP	Higher Xanthoria - Physcia	aerobic chemoheterotrophy unknown	Lichen Bacteria
108	Methylobacterium persicinum	1.63484	chemoheterotrophy	methanol oxidation	XP	Higher Xanthoria - Physcia	chemoheterotrophy methanol oxidation	Lichen Bacteria
109	Methylobacterium pseudosasicola	1.720483	chemoheterotrophy	methanol oxidation	XP	Higher Xanthoria - Physcia	chemoheterotrophy methanol oxidation	Lichen Bacteria
110	Mucilaginibacter sp	2.526938	unknown	unknown	XP	Higher Xanthoria - Physcia	unknown unknown	Lichen Bacteria
111	Nodularia spumigena	2.534671	oxygenic photoautotrophy	unknown	XP	Higher Xanthoria - Physcia	oxygenic photoautotrophy unknown	Lichen Bacteria
112	Compostibacter hankyongensis	2.550991	aerobic chemoheterotrophy	unknown	XP	Higher Xanthoria - Physcia	aerobic chemoheterotrophy unknown	Lichen Bacteria
113	Parviterribacter kavangonensis	2.610219	aerobic chemoheterotrophy	unknown	XP	Higher Xanthoria - Physcia	aerobic chemoheterotrophy unknown	Lichen Bacteria
114	Variovorax boronicumulans	2.692913	aerobic chemoheterotrophy	plant growth promotion	XP	Higher Xanthoria - Physcia	aerobic chemoheterotrophy plant growth promotion	Lichen Bacteria

115	Streptomyces polyantibioticus	2.707325	aerobic chemoheterotrophy	unknown	XP	Higher Xanthoria - Physcia	aerobic chemoheterotrophy unknown	Lichen Bacteria
116	Variovorax paradoxus	2.741463	chemoheterotrophy	arsenic detoxification	XP	Higher Xanthoria - Physcia	chemoheterotrophy arsenic detoxification	Lichen Bacteria
117	Nostoc oromo	2.778268	oxygenic photoautotrophy	nitrogen fixation	XP	Higher Xanthoria - Physcia	oxygenic photoautotrophy nitrogen fixation	Lichen Bacteria
118	Mesorhizobium soli	2.846567	aerobic chemoheterotrophy	ureolysis	XP	Higher Xanthoria - Physcia	aerobic chemoheterotrophy ureolysis	Lichen Bacteria
119	Microvirga rosea	2.901153	aerobic chemoheterotrophy	unknown	XP	Higher Xanthoria - Physcia	aerobic chemoheterotrophy unknown	Lichen Bacteria
120	Ramlibacter solisilvae	2.911285	aerobic chemoheterotrophy	unknown	XP	Higher Xanthoria - Physcia	aerobic chemoheterotrophy unknown	Lichen Bacteria
121	Kallipyga massiliensis	2.968546	animal parasite or symbiones	unknown	XP	Higher Xanthoria - Physcia	animal parasite or symbiones unknown	Lichen Bacteria
122	Acidibacter ferrireducens	2.984731	extremophilic	iron reduction	XP	Higher Xanthoria - Physcia	extremophilic iron reduction	Lichen Bacteria
123	Simplicispira metamorpha	3.005411	aerobic chemoheterotrophy	denitrification	XP	Higher Xanthoria - Physcia	aerobic chemoheterotrophy denitrification	Lichen Bacteria
124	Mucilaginibacter hankyongensis	3.059599	aerobic chemoheterotrophy	unknown	XP	Higher Xanthoria - Physcia	aerobic chemoheterotrophy unknown	Lichen Bacteria
125	Devosia psychrophila	3.203319	aerobic chemoheterotrophy	unknown	XP	Higher Xanthoria - Physcia	aerobic chemoheterotrophy unknown	Lichen Bacteria
126	Rhizobium sp	3.205882	unknown	unknown	XP	Higher Xanthoria - Physcia	unknown unknown	Lichen Bacteria
127	Polaromonas vacuolata	3.232298	chemoheterotrophy	ureolysis	XP	Higher Xanthoria - Physcia	chemoheterotrophy ureolysis	Lichen Bacteria
128	Herbiconiux moechotypicola	3.2695	aerobic chemoheterotrophy	xylanolysis	XP	Higher Xanthoria - Physcia	aerobic chemoheterotrophy xylanolysis	Lichen Bacteria
129	Jahnella thaxteri	3.27899	aerobic chemoheterotrophy	unknown	XP	Higher Xanthoria - Physcia	aerobic chemoheterotrophy unknown	Lichen Bacteria
130	Agreia sp	3.28613	unknown	unknown	XP	Higher Xanthoria - Physcia	unknown unknown	Lichen Bacteria
131	Cellvibrio gandavensis	3.360045	unknown	unknown	XP	Higher Xanthoria - Physcia	unknown unknown	Lichen Bacteria
132	Hymenobacter actinosclerus	3.439069	aerobic chemoheterotrophy	hydrocarbon degradation	XP	Higher Xanthoria - Physcia	aerobic chemoheterotrophy hydrocarbon degradation	Lichen Bacteria
133	Mucilaginibacter mallensis	3.468473	aerobic chemoheterotrophy	adipate degradation	XP	Higher Xanthoria - Physcia	aerobic chemoheterotrophy adipate degradation	Lichen Bacteria
134	Methylobacterium bullatum	3.502571	aerobic chemoheterotrophy	unknown	XP	Higher Xanthoria - Physcia	aerobic chemoheterotrophy unknown	Lichen Bacteria

135	Devosia chinhatensis	3.527617	aerobic chemoheterotrophy	hydrocarbon degradation	XP	Higher Xanthoria - Physcia	aerobic chemoheterotrophy hydrocarbon degradation	Lichen Bacteria
136	Microbacterium aurum	3.538403	aerobic chemoheterotrophy	fermentation	XP	Higher Xanthoria - Physcia	aerobic chemoheterotrophy fermentation	Lichen Bacteria
137	Thermasporomyces composti	3.614634	unknown	unknown	XP	Higher Xanthoria - Physcia	unknown unknown	Lichen Bacteria
138	Prochlorothrix hollandica	3.628794	oxygenic photoautotrophy	nitrogen fixation	XP	Higher Xanthoria - Physcia	oxygenic photoautotrophy nitrogen fixation	Lichen Bacteria
139	Algoriphagus aquaemixtae	3.769698	aerobic chemoheterotrophy	unknown	XP	Higher Xanthoria - Physcia	aerobic chemoheterotrophy unknown	Lichen Bacteria
140	Aetherobacter sp	3.78954	unknown	unknown	XP	Higher Xanthoria - Physcia	unknown unknown	Lichen Bacteria
141	Cytophaga hutchinsonii	3.799503	aerobic chemoheterotrophy	cellulolysis	XP	Higher Xanthoria - Physcia	aerobic chemoheterotrophy cellulolysis	Lichen Bacteria
142	Ferruginibacter alkalilentus	3.820434	aerobic chemoheterotrophy	xenobiotics degradation	XP	Higher Xanthoria - Physcia	aerobic chemoheterotrophy xenobiotics degradation	Lichen Bacteria
143	Rhizobium gei	3.829571	aerobic chemoheterotrophy	unknown	XP	Higher Xanthoria - Physcia	aerobic chemoheterotrophy unknown	Lichen Bacteria
144	Reyranella terrae	3.896324	aerobic chemoheterotrophy	unknown	XP	Higher Xanthoria - Physcia	aerobic chemoheterotrophy unknown	Lichen Bacteria
145	Cellvibrio japonicus	3.984147	aerobic chemoheterotrophy	cellulolysis	XP	Higher Xanthoria - Physcia	aerobic chemoheterotrophy cellulolysis	Lichen Bacteria
146	Mucilaginibacter pedocola	3.987855	aerobic chemoheterotrophy	cellulolysis	XP	Higher Xanthoria - Physcia	aerobic chemoheterotrophy cellulolysis	Lichen Bacteria
147	Luteolibacter pohnpeiensis	4.027802	aerobic chemoheterotrophy	unknown	XP	Higher Xanthoria - Physcia	aerobic chemoheterotrophy unknown	Lichen Bacteria
148	Microcystis aeruginosa	4.043109	oxygenic photoautotrophy	harmful algal blooms	XP	Higher Xanthoria - Physcia	oxygenic photoautotrophy harmful algal blooms	Lichen Bacteria
149	Mucilaginibacter pineti	4.056808	aerobic chemoheterotrophy	cellulolysis	XP	Higher Xanthoria - Physcia	aerobic chemoheterotrophy cellulolysis	Lichen Bacteria
150	Sphingomonas qilianensis	4.223755	aerobic chemoheterotrophy	unknown	XP	Higher Xanthoria - Physcia	aerobic chemoheterotrophy unknown	Lichen Bacteria
151	Mucilaginibacter craterilacus	4.273815	aerobic chemoheterotrophy	unknown	XP	Higher Xanthoria - Physcia	aerobic chemoheterotrophy unknown	Lichen Bacteria
152	Nitrosospira multiformis	4.293871	chemoautotrophy	nitrification	XP	Higher Xanthoria - Physcia	chemoautotrophy nitrification	Lichen Bacteria
153	Rhizobium azooxidifex	4.33581	chemoheterotrophy	nitrification	XP	Higher Xanthoria - Physcia	chemoheterotrophy nitrification	Lichen Bacteria
154	Acidovorax sp	4.357816	unknown	unknown	XP	Higher Xanthoria - Physcia	unknown unknown	Lichen Bacteria

155	Chryseolinea serpens	4.392456	aerobic chemoheterotrophy	xylanolysis	XP	Higher Xanthoria - Physcia	aerobic chemoheterotrophy xylanolysis	Lichen Bacteria
156	Pedobacter changchengzhani	4.42305	aerobic chemoheterotrophy	unknown	XP	Higher Xanthoria - Physcia	aerobic chemoheterotrophy unknown	Lichen Bacteria
157	Luteolibacter gellanilyticus	4.450068	aerobic chemoheterotrophy	unknown	XP	Higher Xanthoria - Physcia	aerobic chemoheterotrophy unknown	Lichen Bacteria
158	Mucilaginibacter endophyticus	4.454052	unknown	unknown	XP	Higher Xanthoria - Physcia	unknown unknown	Lichen Bacteria
159	Mucilaginibacter dorajii	4.523886	aerobic chemoheterotrophy	unknown	XP	Higher Xanthoria - Physcia	aerobic chemoheterotrophy unknown	Lichen Bacteria
160	Chitinimonas koreensis	4.648891	aerobic chemoheterotrophy	unknown	XP	Higher Xanthoria - Physcia	aerobic chemoheterotrophy unknown	Lichen Bacteria
161	Luteibacter rhizovicinus	4.730256	aerobic chemoheterotrophy	plant growth promotion	XP	Higher Xanthoria - Physcia	aerobic chemoheterotrophy plant growth promotion	Lichen Bacteria
162	Polyangium sorediatum	4.847124	aerobic chemoheterotrophy	unknown	XP	Higher Xanthoria - Physcia	aerobic chemoheterotrophy unknown	Lichen Bacteria
163	Noviherbaspirillum soli	4.870651	aerobic chemoheterotrophy	unknown	XP	Higher Xanthoria - Physcia	aerobic chemoheterotrophy unknown	Lichen Bacteria
164	Mucilaginibacter panaciglaebae	4.875595	aerobic chemoheterotrophy	unknown	XP	Higher Xanthoria - Physcia	aerobic chemoheterotrophy unknown	Lichen Bacteria
165	Cellvibrio sp	5.021197	unknown	unknown	XP	Higher Xanthoria - Physcia	unknown unknown	Lichen Bacteria
166	Flavobacterium hydrophilum	5.218657	aerobic chemoheterotrophy	unknown	XP	Higher Xanthoria - Physcia	aerobic chemoheterotrophy unknown	Lichen Bacteria
167	Flavobacterium cerinum	5.383626	aerobic chemoheterotrophy	unknown	XP	Higher Xanthoria - Physcia	aerobic chemoheterotrophy unknown	Lichen Bacteria
168	Cellvibrio zantedeschiae	5.483692	aerobic chemoheterotrophy	unknown	XP	Higher Xanthoria - Physcia	aerobic chemoheterotrophy unknown	Lichen Bacteria
169	Undibacterium amnicola	5.583421	aerobic chemoheterotrophy	unknown	XP	Higher Xanthoria - Physcia	aerobic chemoheterotrophy unknown	Lichen Bacteria
170	Pedobacter glucosidilyticus	5.699135	aerobic chemoheterotrophy	cellulolysis	XP	Higher Xanthoria - Physcia	aerobic chemoheterotrophy cellulolysis	Lichen Bacteria
171	Cyanobacteria Unassigned	5.898067	oxygenic photoautotrophy	unknown	XP	Higher Xanthoria - Physcia	oxygenic photoautotrophy unknown	Lichen Bacteria
172	Pedobacter ginsengiterrae	6.817809	aerobic chemoheterotrophy	unknown	XP	Higher Xanthoria - Physcia	aerobic chemoheterotrophy unknown	Lichen Bacteria
173	Gloeothece membranacea	8.192373	oxygenic photoautotrophy	unknown	XP	Higher Xanthoria - Physcia	oxygenic photoautotrophy unknown	Lichen Bacteria

Supplementary Table 2b

No	fungi	log2fold_change	guild	comparison	code	target microbe
1	Candelaria pacifica	-10.778	lichenized	Higher Candelaria - Physcia	CP	Lichen Fungi
2	Bannozyma sp	-6.12409	soil saprotroph	Higher Candelaria - Physcia	CP	Lichen Fungi
3	Basidiomycota Unassigned	-5.84298	unknown	Higher Candelaria - Physcia	CP	Lichen Fungi
4	Dactylellina parvicollis	-5.69932	animal parasite	Higher Candelaria - Physcia	CP	Lichen Fungi
5	Candelaria concolor	-5.15022	lichenized	Higher Candelaria - Physcia	CP	Lichen Fungi
6	Candelariella sp	-5.05681	lichenized	Higher Candelaria - Physcia	CP	Lichen Fungi
7	Microsporomycetaceae sp	-4.49322	unknown	Higher Candelaria - Physcia	CP	Lichen Fungi
8	Hyalorbilia sp	-4.15318	wood saprotroph	Higher Candelaria - Physcia	CP	Lichen Fungi
9	Atrocalyx sp	-3.93137	wood saprotroph	Higher Candelaria - Physcia	CP	Lichen Fungi
10	Dermateaceae sp	-3.86676	unknown	Higher Candelaria - Physcia	CP	Lichen Fungi
11	Trichomerium sp	-3.50784	foliar endophyte	Higher Candelaria - Physcia	CP	Lichen Fungi
12	Orbiliaceae sp	-3.40362	unknown	Higher Candelaria - Physcia	CP	Lichen Fungi
13	Punctelia borreri	-3.39957	lichenized	Higher Candelaria - Physcia	CP	Lichen Fungi
14	Trichomerium sp	-3.37761	foliar endophyte	Higher Candelaria - Physcia	CP	Lichen Fungi
15	Trypethelium platystomum	-3.35355	lichenized	Higher Candelaria - Physcia	CP	Lichen Fungi
16	Nectriaceae sp	-3.19622	unknown	Higher Candelaria - Physcia	CP	Lichen Fungi
17	Hyalorbilia sp	-3.16673	wood saprotroph	Higher Candelaria - Physcia	CP	Lichen Fungi
18	Aspergillus flavus	-3.08953	unspecified saprotroph	Higher Candelaria - Physcia	CP	Lichen Fungi
19	Exophiala eucalyptigena	-3.03099	animal parasite	Higher Candelaria - Physcia	CP	Lichen Fungi
20	Trypethelium eluteriae	-2.76859	lichenized	Higher Candelaria - Physcia	CP	Lichen Fungi
21	Diaporthe sp	-2.63106	plant pathogen	Higher Candelaria - Physcia	CP	Lichen Fungi
22	Ascomycota Unassigned	-2.57595	unknown	Higher Candelaria - Physcia	CP	Lichen Fungi
23	Cyphellophoraceae sp	-2.52746	unknown	Higher Candelaria - Physcia	CP	Lichen Fungi
24	Geopora sp	-2.33651	ectomycorrhizal	Higher Candelaria - Physcia	CP	Lichen Fungi
25	Cladosporium sp	-1.91624	litter saprotroph	Higher Candelaria - Physcia	CP	Lichen Fungi
26	Physcia alnophila	2.332593	lichenized	Higher Physcia - Candelaria	PC	Lichen Fungi
27	Rinodina teichophila	2.542951	lichenized	Higher Physcia - Candelaria	PC	Lichen Fungi
28	Physcia millegrana	2.769847	lichenized	Higher Physcia - Candelaria	PC	Lichen Fungi
29	Ascomycota Unassigned	2.790357	unknown	Higher Physcia - Candelaria	PC	Lichen Fungi
30	Rinodina luridescens	2.814045	lichenized	Higher Physcia - Candelaria	PC	Lichen Fungi
31	Ascomycota Unassigned	3.09911	unknown	Higher Physcia - Candelaria	PC	Lichen Fungi
32	Physcia dimidiata	3.711568	lichenized	Higher Physcia - Candelaria	PC	Lichen Fungi
33	Physcia dubia	3.85229	lichenized	Higher Physcia - Candelaria	PC	Lichen Fungi
34	Physcia aipolia	3.869284	lichenized	Higher Physcia - Candelaria	PC	Lichen Fungi

35	Kurokawia runcinata	3.902794	unknown	Higher Physcia - Candelaria	PC	Lichen Fungi
36	Tremellales sp	3.934844	unknown	Higher Physcia - Candelaria	PC	Lichen Fungi
37	Physcia caesia	4.216841	lichenized	Higher Physcia - Candelaria	PC	Lichen Fungi
38	Flavopunctelia flaventior	4.347083	lichenized	Higher Physcia - Candelaria	PC	Lichen Fungi
39	Physcia alba	4.362143	lichenized	Higher Physcia - Candelaria	PC	Lichen Fungi
40	Physcia stellaris	4.496095	lichenized	Higher Physcia - Candelaria	PC	Lichen Fungi
41	Physcia sp	4.569024	lichenized	Higher Physcia - Candelaria	PC	Lichen Fungi
42	Physcia orientostellaris	4.573509	lichenized	Higher Physcia - Candelaria	PC	Lichen Fungi
43	Physcia sp	4.608336	lichenized	Higher Physcia - Candelaria	PC	Lichen Fungi
44	Physcia undulata	4.623005	lichenized	Higher Physcia - Candelaria	PC	Lichen Fungi
45	Physcia tribacia	4.685046	lichenized	Higher Physcia - Candelaria	PC	Lichen Fungi
46	Physcia atrostriata	4.737713	lichenized	Higher Physcia - Candelaria	PC	Lichen Fungi
47	Physcia adscendens	5.386762	lichenized	Higher Physcia - Candelaria	PC	Lichen Fungi
48	Physcia jackii	5.387341	lichenized	Higher Physcia - Candelaria	PC	Lichen Fungi
49	Pseudoschismatomma rufescens	7.201892	lichenized	Higher Physcia - Candelaria	PC	Lichen Fungi
50	Pterula gracilis	26.68971	litter saprotroph	Higher Physcia - Candelaria	PC	Lichen Fungi
51	Candelaria pacifica	-11.2887	lichenized	Higher Candelaria - Xanthoria	CX	Lichen Fungi
52	Bannozyma sp	-6.94771	soil_saprotroph	Higher Candelaria - Xanthoria	CX	Lichen Fungi
53	Paranectria oropensis	-6.43587	lichen_parasite	Higher Candelaria - Xanthoria	CX	Lichen Fungi
54	Melanelixia subaurifera	-6.4037	lichenized	Higher Candelaria - Xanthoria	CX	Lichen Fungi
55	Candelaria concolor	-5.49944	lichenized	Higher Candelaria - Xanthoria	CX	Lichen Fungi
56	Candelariella sp	-5.27589	lichenized	Higher Candelaria - Xanthoria	CX	Lichen Fungi
57	Didymocyrtis epiphyscia	-5.27308	lichen_parasite	Higher Candelaria - Xanthoria	CX	Lichen Fungi
58	Trichomerium sp	-4.88373	foliar_endophyte	Higher Candelaria - Xanthoria	CX	Lichen Fungi
59	Exophiala eucalyptigena	-4.58678	animal_parasite	Higher Candelaria - Xanthoria	CX	Lichen Fungi
60	Punctelia borreri	-4.42273	lichenized	Higher Candelaria - Xanthoria	CX	Lichen Fungi
61	Dactylellina parvicollis	-4.37042	animal_parasite	Higher Candelaria - Xanthoria	CX	Lichen Fungi
62	Trichomerium sp	-4.31772	foliar_endophyte	Higher Candelaria - Xanthoria	CX	Lichen Fungi
63	Diaporthe sp	-4.19017	plant_pathogen	Higher Candelaria - Xanthoria	CX	Lichen Fungi
64	Microsporomycetaceae_sp	-4.12148	unknown	Higher Candelaria - Xanthoria	CX	Lichen Fungi
65	Nectriaceae_sp	-4.06545	unknown	Higher Candelaria - Xanthoria	CX	Lichen Fungi
66	Vishniacozyma victoriae	-4.05172	soil_saprotroph	Higher Candelaria - Xanthoria	CX	Lichen Fungi
67	Filobasidium wieringae	-3.99643	unspecified_saprotroph	Higher Candelaria - Xanthoria	CX	Lichen Fungi
68	Knufia perfecta	-3.90569	soil_saprotroph	Higher Candelaria - Xanthoria	CX	Lichen Fungi
69	Vishniacozyma sp	-3.76902	soil_saprotroph	Higher Candelaria - Xanthoria	CX	Lichen Fungi
70	Vishniacozyma sp	-3.69337	soil_saprotroph	Higher Candelaria - Xanthoria	CX	Lichen Fungi
71	Opeltiella rubrisoli	-3.63383	unknown	Higher Candelaria - Xanthoria	CX	Lichen Fungi

72	Trypethelium platystomum	-3.54515	lichenized	Higher Candelaria - Xanthoria	CX	Lichen Fungi
73	Aureobasidium sp	-3.53395	sooty_mold	Higher Candelaria - Xanthoria	CX	Lichen Fungi
74	Atrocalyx sp	-3.51414	wood_saprotroph	Higher Candelaria - Xanthoria	CX	Lichen Fungi
75	Aureobasidium pullulans	-3.30671	sooty_mold	Higher Candelaria - Xanthoria	CX	Lichen Fungi
76	Knufia sp	-3.25721	soil_saprotroph	Higher Candelaria - Xanthoria	CX	Lichen Fungi
77	Aureobasidium sp	-3.19046	sooty_mold	Higher Candelaria - Xanthoria	CX	Lichen Fungi
78	Cladosporium sp	-3.16717	litter_saprotroph	Higher Candelaria - Xanthoria	CX	Lichen Fungi
79	Lichenostigmatales_sp	-3.12473	unknown	Higher Candelaria - Xanthoria	CX	Lichen Fungi
80	Chaetothyriaceae_sp	-3.11453	unknown	Higher Candelaria - Xanthoria	CX	Lichen Fungi
81	Knufia tsunedae	-3.1061	soil_saprotroph	Higher Candelaria - Xanthoria	CX	Lichen Fungi
82	Otidea cantharella	-3.10552	ectomycorrhizal	Higher Candelaria - Xanthoria	CX	Lichen Fungi
83	Hyalorbilia sp	-3.05204	wood_saprotroph	Higher Candelaria - Xanthoria	CX	Lichen Fungi
84	Micarea fallax	-3.05191	lichenized	Higher Candelaria - Xanthoria	CX	Lichen Fungi
85	Cyphellophoraceae_sp	-2.97827	unknown	Higher Candelaria - Xanthoria	CX	Lichen Fungi
86	Geopora sp	-2.96778	ectomycorrhizal	Higher Candelaria - Xanthoria	CX	Lichen Fungi
87	Trypethelium eluteriae	-2.96018	lichenized	Higher Candelaria - Xanthoria	CX	Lichen Fungi
88	Erythrobasidium primogenitum	-2.88156	mycoparasite	Higher Candelaria - Xanthoria	CX	Lichen Fungi
89	Knufia sp	-2.86517	soil_saprotroph	Higher Candelaria - Xanthoria	CX	Lichen Fungi
90	Neodevriesia sp	-2.84759	plant_pathogen	Higher Candelaria - Xanthoria	CX	Lichen Fungi
91	Microstroma bacarum	-2.71292	plant_pathogen	Higher Candelaria - Xanthoria	CX	Lichen Fungi
92	Ascomycota Unassigned	-2.66809	unknown	Higher Candelaria - Xanthoria	CX	Lichen Fungi
93	Ascomycota Unassigned	-2.65806	unknown	Higher Candelaria - Xanthoria	CX	Lichen Fungi
94	Ascomycota Unassigned	-2.57518	unknown	Higher Candelaria - Xanthoria	CX	Lichen Fungi
95	Glomeromyceta Unassigned	-2.28143	unknown	Higher Candelaria - Xanthoria	CX	Lichen Fungi
96	Helotiales_sp	2.308883	unknown	Higher Xanthoria - Candelaria	XC	Lichen Fungi
97	Caloplaca elvebakkiana	2.385084	lichenized	Higher Xanthoria - Candelaria	XC	Lichen Fungi
98	Calogaya sp	2.728298	lichenized	Higher Xanthoria - Candelaria	XC	Lichen Fungi
99	Dufourea dissectula	2.805421	lichenized	Higher Xanthoria - Candelaria	XC	Lichen Fungi
100	Athallia saxifragarum	3.001747	lichenized	Higher Xanthoria - Candelaria	XC	Lichen Fungi
101	Remototrachyna flexilis	3.076795	lichenized	Higher Xanthoria - Candelaria	XC	Lichen Fungi
102	Xanthoria resendei	3.085129	lichenized	Higher Xanthoria - Candelaria	XC	Lichen Fungi
103	Laundonia flavovirescens	3.325859	unknown	Higher Xanthoria - Candelaria	XC	Lichen Fungi
104	Calogaya sp	3.456457	lichenized	Higher Xanthoria - Candelaria	XC	Lichen Fungi
105	Calogaya biatorina_subsp._asiatica	3.510481	lichenized	Higher Xanthoria - Candelaria	XC	Lichen Fungi
106	Fominiella skii	3.727007	unknown	Higher Xanthoria - Candelaria	XC	Lichen Fungi
107	Calogaya pusilla	3.863693	lichenized	Higher Xanthoria - Candelaria	XC	Lichen Fungi
108	Ascomycota Unassigned	3.979487	unknown	Higher Xanthoria - Candelaria	XC	Lichen Fungi

109	Xanthoria parietina	4.101755	lichenized	Higher Xanthoria - Candelaria	XC	Lichen Fungi
110	Ascomycota Unassigned	4.119858	unknown	Higher Xanthoria - Candelaria	XC	Lichen Fungi
111	Xanthoria sp	4.143133	lichenized	Higher Xanthoria - Candelaria	XC	Lichen Fungi
112	Xanthoria calcicola	4.217724	lichenized	Higher Xanthoria - Candelaria	XC	Lichen Fungi
113	Xanthoria ulophyllodes	4.222951	lichenized	Higher Xanthoria - Candelaria	XC	Lichen Fungi
114	Xanthoria sp	4.250896	lichenized	Higher Xanthoria - Candelaria	XC	Lichen Fungi
115	Xanthoria elegans	4.396706	lichenized	Higher Xanthoria - Candelaria	XC	Lichen Fungi
116	Squamulea kiamae	4.412628	lichenized	Higher Xanthoria - Candelaria	XC	Lichen Fungi
117	Ascomycota Unassigned	4.464715	unknown	Higher Xanthoria - Candelaria	XC	Lichen Fungi
118	Dufourea sp	4.536656	lichenized	Higher Xanthoria - Candelaria	XC	Lichen Fungi
119	Athallia holocarpa	4.548632	lichenized	Higher Xanthoria - Candelaria	XC	Lichen Fungi
120	Ascomycota Unassigned	4.888632	unknown	Higher Xanthoria - Candelaria	XC	Lichen Fungi
121	Caloplaca obscurella	5.08491	lichenized	Higher Xanthoria - Candelaria	XC	Lichen Fungi
122	Unguiculariopsis lettaui	5.898931	lichen_parasite	Higher Xanthoria - Candelaria	XC	Lichen Fungi
123	Distocercospora pachyderma	7.137233	plant_pathogen	Higher Xanthoria - Candelaria	XC	Lichen Fungi
124	Pterula gracilis	-8.54613	litter_saprotroph	Higher Physcia - Xanthoria	PX	Lichen Fungi
125	Opeltiella rubrisoli	-4.93544	unknown	Higher Physcia - Xanthoria	PX	Lichen Fungi
126	Filobasidium wieringae	-4.67276	unspecified_saprotroph	Higher Physcia - Xanthoria	PX	Lichen Fungi
127	Tremellales_sp	-4.53603	unknown	Higher Physcia - Xanthoria	PX	Lichen Fungi
128	Vishniacozyma victoriae	-4.46837	soil_saprotroph	Higher Physcia - Xanthoria	PX	Lichen Fungi
129	Physcia dimidiata	-4.28967	lichenized	Higher Physcia - Xanthoria	PX	Lichen Fungi
130	Physcia alba	-3.8319	lichenized	Higher Physcia - Xanthoria	PX	Lichen Fungi
131	Physcia alnophila	-3.71941	lichenized	Higher Physcia - Xanthoria	PX	Lichen Fungi
132	Physcia dubia	-3.67428	lichenized	Higher Physcia - Xanthoria	PX	Lichen Fungi
133	Aureobasidium sp	-3.61965	sooty_mold	Higher Physcia - Xanthoria	PX	Lichen Fungi
134	Vishniacozyma sp	-3.57509	soil_saprotroph	Higher Physcia - Xanthoria	PX	Lichen Fungi
135	Kurokawia runcinata	-3.57054	unknown	Higher Physcia - Xanthoria	PX	Lichen Fungi
136	Physcia aipolia	-3.48996	lichenized	Higher Physcia - Xanthoria	PX	Lichen Fungi
137	Physcia atrostriata	-3.38663	lichenized	Higher Physcia - Xanthoria	PX	Lichen Fungi
138	Neodevriesia sp	-3.37252	plant_pathogen	Higher Physcia - Xanthoria	PX	Lichen Fungi
139	Physcia stellaris	-3.35006	lichenized	Higher Physcia - Xanthoria	PX	Lichen Fungi
140	Flavopunctelia flaventior	-3.33315	lichenized	Higher Physcia - Xanthoria	PX	Lichen Fungi
141	Aureobasidium sp	-3.33238	sooty_mold	Higher Physcia - Xanthoria	PX	Lichen Fungi
142	Micarea fallax	-3.28363	lichenized	Higher Physcia - Xanthoria	PX	Lichen Fungi
143	Physcia tribacia	-3.27228	lichenized	Higher Physcia - Xanthoria	PX	Lichen Fungi
144	Rinodina teichophila	-3.25643	lichenized	Higher Physcia - Xanthoria	PX	Lichen Fungi
145	Physcia sp	-3.24179	lichenized	Higher Physcia - Xanthoria	PX	Lichen Fungi

146	Aureobasidium pullulans	-3.2306	sooty_mold	Higher Physcia - Xanthoria	PX	Lichen Fungi
147	Knufia perfecta	-3.21104	soil_saprotroph	Higher Physcia - Xanthoria	PX	Lichen Fungi
148	Microstroma bacarum	-3.08461	plant_pathogen	Higher Physcia - Xanthoria	PX	Lichen Fungi
149	Physcia caesia	-3.07857	lichenized	Higher Physcia - Xanthoria	PX	Lichen Fungi
150	Glomeromyceta Unassigned	-3.05443	unknown	Higher Physcia - Xanthoria	PX	Lichen Fungi
151	Physcia sp	-3.03471	lichenized	Higher Physcia - Xanthoria	PX	Lichen Fungi
152	Physcia orientostellaris	-3.00186	lichenized	Higher Physcia - Xanthoria	PX	Lichen Fungi
153	Physcia undulata	-2.99779	lichenized	Higher Physcia - Xanthoria	PX	Lichen Fungi
154	Lichenostigmatales_sp	-2.9776	unknown	Higher Physcia - Xanthoria	PX	Lichen Fungi
155	Ascomycota Unassigned	-2.94607	unknown	Higher Physcia - Xanthoria	PX	Lichen Fungi
156	Physcia jackii	-2.89888	lichenized	Higher Physcia - Xanthoria	PX	Lichen Fungi
157	Knufia sp	-2.87891	soil_saprotroph	Higher Physcia - Xanthoria	PX	Lichen Fungi
158	Physcia millegrana	-2.76151	lichenized	Higher Physcia - Xanthoria	PX	Lichen Fungi
159	Erythrobasidium primogenitum	-2.72901	mycoparasite	Higher Physcia - Xanthoria	PX	Lichen Fungi
160	Otidea cantharella	-2.63509	ectomycorrhizal	Higher Physcia - Xanthoria	PX	Lichen Fungi
161	Ascomycota Unassigned	-2.51524	unknown	Higher Physcia - Xanthoria	PX	Lichen Fungi
162	Ascomycota Unassigned	-2.44747	unknown	Higher Physcia - Xanthoria	PX	Lichen Fungi
163	Rinodina luridescens	-2.40926	lichenized	Higher Physcia - Xanthoria	PX	Lichen Fungi
164	Knufia tsunedae	-2.39977	soil_saprotroph	Higher Physcia - Xanthoria	PX	Lichen Fungi
165	Vishniacozyma sp	-2.35697	soil_saprotroph	Higher Physcia - Xanthoria	PX	Lichen Fungi
166	Chaetothyriaceae_sp	-2.34715	unknown	Higher Physcia - Xanthoria	PX	Lichen Fungi
167	Physcia adscendens	-2.31934	lichenized	Higher Physcia - Xanthoria	PX	Lichen Fungi
168	Knufia sp	-2.17778	soil_saprotroph	Higher Physcia - Xanthoria	PX	Lichen Fungi
169	Punctelia borreri	-1.02504	lichenized	Higher Physcia - Xanthoria	PX	Lichen Fungi
170	Caloplaca elvebakkiana	2.637537	lichenized	Higher Xanthoria - Physcia	XP	Lichen Fungi
171	Helotiales_sp	2.69721	unknown	Higher Xanthoria - Physcia	XP	Lichen Fungi
172	Dufourea dissectula	2.794474	lichenized	Higher Xanthoria - Physcia	XP	Lichen Fungi
173	Aspergillus flavus	2.963756	unspecified_saprotroph	Higher Xanthoria - Physcia	XP	Lichen Fungi
174	Calogaya sp	3.038871	lichenized	Higher Xanthoria - Physcia	XP	Lichen Fungi
175	Athallia saxifragarum	3.043687	lichenized	Higher Xanthoria - Physcia	XP	Lichen Fungi
176	Dermateaceae_sp	3.06387	unknown	Higher Xanthoria - Physcia	XP	Lichen Fungi
177	Remototrachyna flexilis	3.067504	lichenized	Higher Xanthoria - Physcia	XP	Lichen Fungi
178	Xanthoria resendei	3.073114	lichenized	Higher Xanthoria - Physcia	XP	Lichen Fungi
179	Hyalorbilia sp	3.239254	wood_saprotroph	Higher Xanthoria - Physcia	XP	Lichen Fungi
180	Calogaya biatorina_subsp._asiatica	3.499475	lichenized	Higher Xanthoria - Physcia	XP	Lichen Fungi
181	Fominiella skii	3.771486	unknown	Higher Xanthoria - Physcia	XP	Lichen Fungi
182	Laundonia flavovirescens	3.835973	unknown	Higher Xanthoria - Physcia	XP	Lichen Fungi

183	Ascomycota Unassigned	3.850723	unknown	Higher Xanthoria - Physcia	XP	Lichen Fungi
184	Ascomycota Unassigned	4.059827	unknown	Higher Xanthoria - Physcia	XP	Lichen Fungi
185	Ascomycota Unassigned	4.161193	unknown	Higher Xanthoria - Physcia	XP	Lichen Fungi
186	Orbiliaceae_sp	4.167051	unknown	Higher Xanthoria - Physcia	XP	Lichen Fungi
187	Squamulea kiamae	4.249233	lichenized	Higher Xanthoria - Physcia	XP	Lichen Fungi
188	Dufourea sp	4.31897	lichenized	Higher Xanthoria - Physcia	XP	Lichen Fungi
189	Ascomycota Unassigned	4.463106	unknown	Higher Xanthoria - Physcia	XP	Lichen Fungi
190	Basidiomycota Unassigned	4.715938	unknown	Higher Xanthoria - Physcia	XP	Lichen Fungi
191	Xanthoria parietina	4.72794	lichenized	Higher Xanthoria - Physcia	XP	Lichen Fungi
192	Xanthoria sp	4.732861	lichenized	Higher Xanthoria - Physcia	XP	Lichen Fungi
193	Calogaya sp	4.756544	lichenized	Higher Xanthoria - Physcia	XP	Lichen Fungi
194	Xanthoria sp	4.860824	lichenized	Higher Xanthoria - Physcia	XP	Lichen Fungi
195	Xanthoria ulophyllodes	4.875699	lichenized	Higher Xanthoria - Physcia	XP	Lichen Fungi
196	Calogaya pusilla	4.902399	lichenized	Higher Xanthoria - Physcia	XP	Lichen Fungi
197	Xanthoria calcicola	4.95351	lichenized	Higher Xanthoria - Physcia	XP	Lichen Fungi
198	Xanthoria elegans	5.05735	lichenized	Higher Xanthoria - Physcia	XP	Lichen Fungi
199	Athallia holocarpa	5.377783	lichenized	Higher Xanthoria - Physcia	XP	Lichen Fungi
200	Unguiculariopsis lettaui	5.463556	lichen_parasite	Higher Xanthoria - Physcia	XP	Lichen Fungi
201	Distocercospora pachyderma	6.982218	plant_pathogen	Higher Xanthoria - Physcia	XP	Lichen Fungi

Supplementary Table 3

No	Network	Node	Edge	Positive correlation	Negative correlation	Diameter	radius	Heterogeneity	Density
1	Low urbanized	37	26	26	0	2	1	0.425	0.600
2	Medium urbanized	121	151	128	23	13	7	0.824	0.046
3	Highly urbanized	75	58	45	13	8	4	0.650	0.125
4	Candelaria concolor	160	175	113	62	10	5	0.676	0.057
5	Xanthoria parietina	62	74	63	11	2	1	0.444	0.600
6	Physcia adscendens	51	73	59	14	5	3	0.700	0.251

Supplementary Table 4

asv1	asv2	cor	pval	phylum_1	phylum_2	species_1	species_2	type_interaction	urbanization_level
Fung-769	Fung-17174	0.9103	0.001	Ascomycota	Ascomycota	Unassigned	Exophiala sp	positive	low
Fung-3315	Fung-17177	0.9652	0.001	Ascomycota	Ascomycota	Exophiala sp	Exophiala tremulae	positive	low
Fung-3375	Fung-17967	0.9207	0.001	Ascomycota	Ascomycota	Penicillium sp	Penicillium amphipolaria	positive	low
Fung-18138	Fung-18058	0.9454	0.001	Ascomycota	Ascomycota	Penicillium fundyense	Penicillium citreosulfuratum	positive	low
Fung-10239	Fung-2070	0.9702	0.001	Ascomycota	Ascomycota	Cladosporium cladosporioides	Cladosporium sp	positive	low
Fung-1136	Fung-34569	0.921	0.001	Ascomycota	Ascomycota	Unassigned	Clonostachys sp	positive	low
Fung-6046	Fung-36424	0.9182	0.001	Ascomycota	Ascomycota	Fusarium sp	Fusarium oxysporum	positive	low
Fung-6046	Fung-36484	0.956	0.001	Ascomycota	Ascomycota	Fusarium sp	Fusarium sp	positive	low
Fung-36424	Fung-36484	0.914	0.001	Ascomycota	Ascomycota	Fusarium oxysporum	Fusarium sp	positive	low
Fung-61251	Fung-8972	0.9114	0.001	Mucoromycota	Mucoromycota	Absidia cylindrospora	Absidia sp	positive	low
Fung-59990	Fung-8598	0.9475	0.001	Basidiomycota	Basidiomycota	Saitozyma podzolica	Saitozyma sp	positive	low
Bac-114975	Bac-1365606	0.9033	0.001	Firmicutes	Firmicutes	Sporosarcina aquimarina	Sporosarcina siberiensis	positive	low
Bac-114975	Bac-1569	0.908	0.001	Firmicutes	Firmicutes	Sporosarcina aquimarina	Sporosarcina sp	positive	low
Bac-582850	Bac-1365606	0.9102	0.001	Firmicutes	Firmicutes	Sporosarcina luteola	Sporosarcina siberiensis	positive	low
Bac-582850	Bac-1569	0.9263	0.001	Firmicutes	Firmicutes	Sporosarcina luteola	Sporosarcina sp	positive	low
Bac-1365606	Bac-1569	0.9396	0.001	Firmicutes	Firmicutes	Sporosarcina siberiensis	Sporosarcina sp	positive	low
Bac-334736	Bac-1569	0.9112	0.001	Firmicutes	Firmicutes	Sporosarcina soli	Sporosarcina sp	positive	low
Bac-467093	Bac-467091	0.915	0.001	Actinobacteria	Actinobacteria	Ilumatobacter nonamiensis	Ilumatobacter fluminis	positive	low
Bac-166793	Bac-1395955	0.9295	0.001	Actinobacteria	Actinobacteria	Solirubrobacter pauli	Solirubrobacter taibaiensis	positive	low

Bac-166793	Bac-363832	0.9373	0.001	Actinobacteria	Actinobacteria	Solirubrobacter pauli	Solirubrobacter soli	positive	low
Bac-166793	Bac-207599	0.9252	0.001	Actinobacteria	Actinobacteria	Solirubrobacter pauli	Solirubrobacter sp	positive	low
Bac-363832	Bac-207599	0.9573	0.001	Actinobacteria	Actinobacteria	Solirubrobacter soli	Solirubrobacter sp	positive	low
Bac-1640866	Bac-588673	0.9065	0.001	Actinobacteria	Actinobacteria	Parviterribacter multiflagellatus	Unassigned	positive	low
Bac-1002870	Bac-201174	0.9041	0.001	Actinobacteria	Actinobacteria	Gaiella occulta	Unassigned	positive	low
Bac-57003	Bac-57002	0.9552	0.001	Proteobacteria	Proteobacteria	Amaricoccus veronensis	Amaricoccus tamworthensis	positive	low
Bac-57003	Bac-56999	0.9037	0.001	Proteobacteria	Proteobacteria	Amaricoccus veronensis	Amaricoccus sp	positive	low
Fung-196	Bac-191494	-0.8405	0.001	Ascomycota	Actinobacteria	Unassigned	Conexibacter sp	negative	medium
Fung-2244	Bac-616952	-0.7909	0.001	Ascomycota	Proteobacteria	Arxiella sp	Bauldia consociata	negative	medium
Fung-39201	Bac-126348	-0.7899	0.001	Ascomycota	Proteobacteria	Mammaria echinobotryoides	Cellvibrio gandavensis	negative	medium
Fung-196	Bac-1002870	-0.7882	0.001	Ascomycota	Actinobacteria	Unassigned	Gaiella occulta	negative	medium
Fung-196	Bac-201174	-0.7865	0.001	Ascomycota	Actinobacteria	Unassigned	Unassigned	negative	medium
Fung-34944	Bac-1742993	-0.783	0.001	Ascomycota	Actinobacteria	Marquandomyces marquandii	Pseudarthrobacter sp	negative	medium
Fung-12920	Bac-616952	-0.7821	0.001	Ascomycota	Proteobacteria	Coniothyrium sp	Bauldia consociata	negative	medium
Fung-17108	Bac-667306	-0.7766	0.001	Ascomycota	Actinobacteria	Cladophialophora sp	Aciditerrimonas ferrireducens	negative	medium
Fung-196	Bac-1640866	-0.7754	0.001	Ascomycota	Actinobacteria	Unassigned	Parviterribacter multiflagellatus	negative	medium
Fung-772	Bac-667306	-0.771	0.001	Ascomycota	Actinobacteria	Unassigned	Aciditerrimonas ferrireducens	negative	medium

Fung-60081	Bac-28221	-0.7697	0.001	Basidiomycota	Proteobacteria	Trichosporon sp	Unassigned	negative	medium
Fung-16981	Bac-676517	-0.7667	0.001	Ascomycota	Proteobacteria	Chaetothyriales_sp	Rickettsiella massiliensis	negative	medium
Fung-3315	Bac-676517	-0.7658	0.001	Ascomycota	Proteobacteria	Exophiala sp	Rickettsiella massiliensis	negative	medium
Fung-18037	Bac-616952	-0.7655	0.001	Ascomycota	Proteobacteria	Penicillium castellonense	Bauldia consociata	negative	medium
Fung-18261	Bac-1236	-0.761	0.001	Ascomycota	Proteobacteria	Penicillium miczynskii	Unassigned	negative	medium
Fung-196	Bac-696763	-0.7586	0.001	Ascomycota	Actinobacteria	Unassigned	Thermasporomyces composti	negative	medium
Fung-3315	Bac-1383030	-0.7572	0.001	Ascomycota	Proteobacteria	Exophiala sp	Pseudoxanthomonas gei	negative	medium
Fung-17177	Bac-1383030	-0.7571	0.001	Ascomycota	Proteobacteria	Exophiala tremulae	Pseudoxanthomonas gei	negative	medium
Fung-59544	Bac-2789776	-0.7552	0.001	Basidiomycota	Actinobacteria	Naganishia brisbanensis	Actinomarinicola tropica	negative	medium
Fung-17598	Bac-2665496	-0.7532	0.001	Ascomycota	Actinobacteria	Aspergillus inflatus	Streptomyces carminius	negative	medium
Fung-196	Bac-452623	-0.7528	0.001	Ascomycota	Actinobacteria	Unassigned	Pseudoclavibacter soli	negative	medium
Fung-292	Bac-1742993	-0.7505	0.001	Ascomycota	Actinobacteria	Unassigned	Pseudarthrobacter sp	negative	medium
Fung-60756	Bac-467093	-0.75	0.001	Unassigned	Actinobacteria	Unassigned	Ilumatobacter nonamiensis	negative	medium
Fung-6043	Bac-1855912	0.7505	0.001	Ascomycota	Acidobacteria	Dactylonectria sp	Luteitalea pratensis	positive	medium
Fung-6043	Bac-1002870	0.7507	0.001	Ascomycota	Actinobacteria	Dactylonectria sp	Gaiella occulta	positive	medium
Fung-8544	Bac-115979	0.751	0.001	Basidiomycota	Firmicutes	Naganishia sp	Niallia nealsonii	positive	medium

Fung-16029	Bac-675609	0.7515	0.001	Ascomycota	Proteobacteria	Pyrenochaetopsis tabarestanensis	Nevskia terrae	positive	medium
Fung-17177	Bac-1002870	0.7516	0.001	Ascomycota	Actinobacteria	Exophiala tremulae	Gaiella occulta	positive	medium
Fung-17177	Bac-467091	0.7523	0.001	Ascomycota	Actinobacteria	Exophiala tremulae	Ilumatobacter fluminis	positive	medium
Fung-14767	Bac-186650	0.7529	0.001	Ascomycota	Proteobacteria	Ampelomyces quisqualis	Microvirga sp	positive	medium
Fung-51814	Bac-75309	0.7531	0.001	Basidiomycota	Proteobacteria	Scleroderma areolatum	Rhodanobacter sp	positive	medium
Fung-6043	Bac-467091	0.754	0.001	Ascomycota	Actinobacteria	Dactylonectria sp	Ilumatobacter fluminis	positive	medium
Fung-34569	Bac-2789776	0.7542	0.001	Ascomycota	Actinobacteria	Clonostachys sp	Actinomarinicola tropica	positive	medium
Fung-6043	Bac-55968	0.7543	0.001	Ascomycota	Actinobacteria	Dactylonectria sp	Leucobacter sp	positive	medium
Fung-34944	Bac-85023	0.7547	0.001	Ascomycota	Actinobacteria	Marquandomyces marquandii	Unassigned	positive	medium
Fung-59544	Bac-455078	0.7549	0.001	Basidiomycota	Bacteroidetes	Naganishia brisbanensis	Rhodocytophaga aerolata	positive	medium
Fung-59544	Bac-1123067	0.7551	0.001	Basidiomycota	Proteobacteria	Naganishia brisbanensis	Rubellimicrobium mesophilum	positive	medium
Fung-17177	Bac-40544	0.7554	0.001	Ascomycota	Proteobacteria	Exophiala tremulae	Sutterella sp	positive	medium
Fung-16029	Bac-85023	0.757	0.001	Ascomycota	Actinobacteria	Pyrenochaetopsis tabarestanensis	Unassigned	positive	medium
Fung-17177	Bac-1384459	0.758	0.001	Ascomycota	Proteobacteria	Exophiala tremulae	Methyloceanibacter caenitepidi	positive	medium
Fung-292	Bac-57003	0.7589	0.001	Ascomycota	Proteobacteria	Unassigned	Amaricoccus veronensis	positive	medium
Fung-2244	Bac-174951	0.7593	0.001	Ascomycota	Proteobacteria	Arxiella sp	Ramlibacter sp	positive	medium
Fung-8544	Bac-1854496	0.7595	0.001	Basidiomycota	Proteobacteria	Naganishia sp	Microvirga soli	positive	medium
Fung-34569	Bac-467091	0.7603	0.001	Ascomycota	Actinobacteria	Clonostachys sp	Ilumatobacter fluminis	positive	medium
Fung-772	Bac-1742993	0.7622	0.001	Ascomycota	Actinobacteria	Unassigned	Pseudarthrobacter sp	positive	medium
Fung-18037	Bac-2800373	0.763	0.001	Ascomycota	Firmicutes	Penicillium castellonense	Priestia sp	positive	medium
Fung-59544	Bac-2086471	0.7641	0.001	Basidiomycota	Bacteroidetes	Naganishia brisbanensis	Adhaeribacter swui	positive	medium

Fung-10146	Bac-1412147	0.7641	0.001	Ascomycota	Firmicutes	Capnodiales_gen_Incertae_sedis Capnodiales_sp	Aquibacillus salifodinae	positive	medium
Fung-59553	Bac-115979	0.7653	0.001	Basidiomycota	Firmicutes	Naganishia sp	Niallia nealsonii	positive	medium
Fung-61125	Bac-31953	0.7655	0.001	Mortierellomycota	Actinobacteria	Mortierella alpina	Unassigned	positive	medium
Fung-59544	Bac-115979	0.7656	0.001	Basidiomycota	Firmicutes	Naganishia brisbanensis	Niallia nealsonii	positive	medium
Fung-59544	Bac-1854496	0.7668	0.001	Basidiomycota	Proteobacteria	Naganishia brisbanensis	Microvirga soli	positive	medium
Fung-10440	Bac-2800373	0.7671	0.001	Ascomycota	Firmicutes	Coniosporium sp	Priestia sp	positive	medium
Fung-10440	Bac-115979	0.7678	0.001	Ascomycota	Firmicutes	Coniosporium sp	Niallia nealsonii	positive	medium
Fung-15081	Bac-393310	0.7687	0.001	Ascomycota	Proteobacteria	Phaeosphaeriopsis sp	Skermanella aerolata	positive	medium
Fung-2244	Bac-760117	0.7689	0.001	Ascomycota	Proteobacteria	Arxiella sp	Massilia consociata	positive	medium
Fung-16974	Bac-1017273	0.7692	0.001	Ascomycota	Firmicutes	Bacillicladium lobatum	Domibacillus enclensis	positive	medium
Fung-16029	Bac-696763	0.7704	0.001	Ascomycota	Actinobacteria	Pyrenochaetopsis tabarestanensis	Thermasporomyces composti	positive	medium
Fung-10146	Bac-1433999	0.7705	0.001	Ascomycota	Firmicutes	Capnodiales_gen_Incertae_sedis Capnodiales_sp	Domibacillus sp	positive	medium
Fung-2244	Bac-149698	0.771	0.001	Ascomycota	Proteobacteria	Arxiella sp	Massilia sp	positive	medium
Fung-36481	Bac-204072	0.7712	0.001	Ascomycota	Proteobacteria	Fusarium solani	Ramlibacter henchirensis	positive	medium
Fung-10440	Bac-1123067	0.7789	0.001	Ascomycota	Proteobacteria	Coniosporium sp	Rubellimicrobium mesophilum	positive	medium
Fung-10648	Bac-1017273	0.7796	0.001	Ascomycota	Firmicutes	Pseudoarthrographis sp	Domibacillus enclensis	positive	medium
Fung-10146	Bac-1123067	0.7804	0.001	Ascomycota	Proteobacteria	Capnodiales_gen_Incertae_sedis Capnodiales_sp	Rubellimicrobium mesophilum	positive	medium
Fung-3315	Bac-1855912	0.7805	0.001	Ascomycota	Acidobacteria	Exophiala sp	Luteitalea pratensis	positive	medium
Fung-10146	Bac-2086471	0.7815	0.001	Ascomycota	Bacteroidetes	Capnodiales_gen_Incertae_sedis Capnodiales_sp	Adhaeribacter swui	positive	medium
Fung-10146	Bac-1742993	0.7819	0.001	Ascomycota	Actinobacteria	Capnodiales_gen_Incertae_sedis Capnodiales_sp	Pseudarthrobacter sp	positive	medium
Fung-34569	Bac-696763	0.7826	0.001	Ascomycota	Actinobacteria	Clonostachys sp	Thermasporomyces composti	positive	medium

Fung-10440	Bac-1742993	0.7852	0.001	Ascomycota	Actinobacteria	Coniosporium sp	Pseudarthrobacter sp	positive	medium
Fung-10440	Bac-2086471	0.7873	0.001	Ascomycota	Bacteroidetes	Coniosporium sp	Adhaeribacter swui	positive	medium
Fung-27781	Bac-2800373	0.7884	0.001	Ascomycota	Firmicutes	Thelebolus sp	Priestia sp	positive	medium
Fung-10146	Bac-1017273	0.7886	0.001	Ascomycota	Firmicutes	Capnodiales_gen_Incertae_sedis Capnodiales_sp	Domibacillus enclensis	positive	medium
Fung-185	Bac-186650	0.7919	0.001	Ascomycota	Proteobacteria	Unassigned	Microvirga sp	positive	medium
Fung-10146	Bac-115979	0.7927	0.001	Ascomycota	Firmicutes	Capnodiales_gen_Incertae_sedis Capnodiales_sp	Niallia nealsonii	positive	medium
Fung-16971	Bac-115979	0.7987	0.001	Ascomycota	Firmicutes	Anthracina saxicola	Niallia nealsonii	positive	medium
Fung-17177	Bac-1759365	0.7997	0.001	Ascomycota	Actinobacteria	Exophiala tremulae	Parviterribacter sp	positive	medium
Fung-36481	Bac-1442381	0.8023	0.001	Ascomycota	Proteobacteria	Fusarium solani	Dankookia rubra	positive	medium
Fung-8544	Bac-1742993	0.8076	0.001	Basidiomycota	Actinobacteria	Naganishia sp	Pseudarthrobacter sp	positive	medium
Fung-16974	Bac-2800373	0.8084	0.001	Ascomycota	Firmicutes	Bacillicladium lobatum	Priestia sp	positive	medium
Fung-59579	Bac-393310	0.8093	0.001	Basidiomycota	Proteobacteria	Solicoccozyma gelidoterrea	Skermanella aerolata	positive	medium
Fung-17177	Bac-1855912	0.8106	0.001	Ascomycota	Acidobacteria	Exophiala tremulae	Luteitalea pratensis	positive	medium
Fung-37205	Bac-85023	0.8114	0.001	Ascomycota	Actinobacteria	Purpureocillium lilacinum	Unassigned	positive	medium
Fung-10146	Bac-2800373	0.8156	0.001	Ascomycota	Firmicutes	Capnodiales_gen_Incertae_sedis Capnodiales_sp	Priestia sp	positive	medium
Fung-59544	Bac-1742993	0.8337	0.001	Basidiomycota	Actinobacteria	Naganishia brisbanensis	Pseudarthrobacter sp	positive	medium
Bac-1229157	Bac-1616821	0.9017	0.001	Firmicutes	Firmicutes	Tumebacillus soli	Tumebacillus luteolus	positive	medium
Bac-1855912	Bac-40544	0.9019	0.001	Acidobacteria	Proteobacteria	Luteitalea pratensis	Sutterella sp	positive	medium
Fung-6046	Fung-36484	0.9025	0.001	Ascomycota	Ascomycota	Fusarium sp	Fusarium sp	positive	medium
Bac-85023	Bac-201174	0.9034	0.001	Actinobacteria	Actinobacteria	Unassigned	Unassigned	positive	medium
Bac-490573	Bac-207599	0.9035	0.001	Actinobacteria	Actinobacteria	Solirubrobacter ginsenosidimutans	Solirubrobacter sp	positive	medium

Bac-1803212	Bac-1474	0.9042	0.001	Firmicutes	Firmicutes	Sporosarcina terrae	Sporosarcina pasteurii	positive	medium
Fung-59990	Fung-8598	0.9043	0.001	Basidiomycota	Basidiomycota	Saitozyma podzolica	Saitozyma sp	positive	medium
Bac-452623	Bac-1839	0.9047	0.001	Actinobacteria	Actinobacteria	Pseudoclavibacter soli	Nocardioides sp	positive	medium
Bac-1474	Bac-114975	0.9049	0.001	Firmicutes	Firmicutes	Sporosarcina pasteurii	Sporosarcina aquimarina	positive	medium
Bac-2665496	Bac-56359	0.9055	0.001	Actinobacteria	Proteobacteria	Streptomyces carminius	Sandaracinobacter sibiricus	positive	medium
Bac-1002870	Bac-191494	0.9056	0.001	Actinobacteria	Actinobacteria	Gaiella occulta	Conexibacter sp	positive	medium
Bac-1839	Bac-201174	0.9058	0.001	Actinobacteria	Actinobacteria	Nocardioides sp	Unassigned	positive	medium
Bac-467091	Bac-452623	0.9058	0.001	Actinobacteria	Actinobacteria	Ilumatobacter fluminis	Pseudoclavibacter soli	positive	medium
Bac-467976	Bac-696763	0.9085	0.001	Actinobacteria	Actinobacteria	Iamia majanohamensis	Thermasporomyces composti	positive	medium
Bac-696763	Bac-1760	0.9089	0.001	Actinobacteria	Actinobacteria	Thermasporomyces composti	Unassigned	positive	medium
Fung-8565	Fung-59731	0.9103	0.001	Basidiomycota	Basidiomycota	Vishniacozyma sp	Vishniacozyma victoriae	positive	medium
Bac-1612172	Bac-1002870	0.9106	0.001	Acidobacteria	Actinobacteria	Vicinamibacter silvestris	Gaiella occulta	positive	medium
Bac-1395955	Bac-490573	0.9107	0.001	Actinobacteria	Actinobacteria	Solirubrobacter taibaiensis	Solirubrobacter ginsenosidimutans	positive	medium
Bac-1760	Bac-1002870	0.9118	0.001	Actinobacteria	Actinobacteria	Unassigned	Gaiella occulta	positive	medium
Bac-85023	Bac-1784839	0.9128	0.001	Actinobacteria	Actinobacteria	Unassigned	Tenggerimyces sp	positive	medium
Bac-166793	Bac-490573	0.9132	0.001	Actinobacteria	Actinobacteria	Solirubrobacter pauli	Solirubrobacter ginsenosidimutans	positive	medium
Bac-1839	Bac-1002870	0.9132	0.001	Actinobacteria	Actinobacteria	Nocardioides sp	Gaiella occulta	positive	medium
Bac-1784839	Bac-696763	0.9132	0.001	Actinobacteria	Actinobacteria	Tenggerimyces sp	Thermasporomyces composti	positive	medium
Bac-452623	Bac-201174	0.9133	0.001	Actinobacteria	Actinobacteria	Pseudoclavibacter soli	Unassigned	positive	medium
Bac-57002	Bac-56999	0.9146	0.001	Proteobacteria	Proteobacteria	Amaricoccus tamworthensis	Amaricoccus sp	positive	medium
Fung-10354	Fung-2070	0.9146	0.001	Ascomycota	Ascomycota	Cladosporium sp	Cladosporium sp	positive	medium

Fung-10907	Fung-2244	0.9159	0.001	Ascomycota	Ascomycota	Arxiella longispora	Arxiella sp	positive	medium
Bac-334736	Bac-1569	0.916	0.001	Firmicutes	Firmicutes	Sporosarcina soli	Sporosarcina sp	positive	medium
Bac-57003	Bac-56999	0.918	0.001	Proteobacteria	Proteobacteria	Amaricoccus veronensis	Amaricoccus sp	positive	medium
Bac-1459	Bac-1569	0.9191	0.001	Firmicutes	Firmicutes	Sporosarcina globispora	Sporosarcina sp	positive	medium
Bac-1002870	Bac-588673	0.9198	0.001	Actinobacteria	Actinobacteria	Gaiella occulta	Unassigned	positive	medium
Bac-1612172	Bac-69541	0.9199	0.001	Acidobacteria	Proteobacteria	Vicinamibacter silvestris	Unassigned	positive	medium
Fung-769	Fung-17177	0.921	0.001	Ascomycota	Ascomycota	Unassigned	Exophiala tremulae	positive	medium
Fung-769	Fung-3315	0.9211	0.001	Ascomycota	Ascomycota	Unassigned	Exophiala sp	positive	medium
Bac-582850	Bac-1365606	0.9213	0.001	Firmicutes	Firmicutes	Sporosarcina luteola	Sporosarcina siberiensis	positive	medium
Fung-2244	Fung-10909	0.9232	0.001	Ascomycota	Ascomycota	Arxiella sp	Arxiella sp	positive	medium
Bac-1395955	Bac-363832	0.9242	0.001	Actinobacteria	Actinobacteria	Solirubrobacter taibaiensis	Solirubrobacter soli	positive	medium
Fung-10146	Fung-10440	0.9244	0.001	Ascomycota	Ascomycota	Capnodiales_gen_Incertae_sedis Capnodiales_sp	Coniosporium sp	positive	medium
Bac-85023	Bac-1002870	0.9247	0.001	Actinobacteria	Actinobacteria	Unassigned	Gaiella occulta	positive	medium
Bac-452623	Bac-1784839	0.9249	0.001	Actinobacteria	Actinobacteria	Pseudoclavibacter soli	Tenggerimyces sp	positive	medium
Bac-582850	Bac-1569	0.9289	0.001	Firmicutes	Firmicutes	Sporosarcina luteola	Sporosarcina sp	positive	medium
Bac-191494	Bac-1640866	0.9292	0.001	Actinobacteria	Actinobacteria	Conexibacter sp	Parviterribacter multiflagellatus	positive	medium
Bac-1002870	Bac-1640866	0.9313	0.001	Actinobacteria	Actinobacteria	Gaiella occulta	Parviterribacter multiflagellatus	positive	medium
Fung-10354	Fung-10239	0.9321	0.001	Ascomycota	Ascomycota	Cladosporium sp	Cladosporium cladosporioides	positive	medium
Fung-8544	Fung-59544	0.9339	0.001	Basidiomycota	Basidiomycota	Naganishia sp	Naganishia brisbanensis	positive	medium
Bac-467091	Bac-696763	0.9356	0.001	Actinobacteria	Actinobacteria	Ilumatobacter fluminis	Thermasporomyces composti	positive	medium
Bac-1784839	Bac-1002870	0.9357	0.001	Actinobacteria	Actinobacteria	Tenggerimyces sp	Gaiella occulta	positive	medium

Bac-1365606	Bac-1569	0.9385	0.001	Firmicutes	Firmicutes	Sporosarcina siberiensis	Sporosarcina sp	positive	medium
Fung-18138	Fung-18058	0.9389	0.001	Ascomycota	Ascomycota	Penicillium fundyense	Penicillium citreosulfuratum	positive	medium
Bac-114975	Bac-582850	0.939	0.001	Firmicutes	Firmicutes	Sporosarcina aquimarina	Sporosarcina luteola	positive	medium
Bac-696763	Bac-1002870	0.9397	0.001	Actinobacteria	Actinobacteria	Thermasporomyces composti	Gaiella occulta	positive	medium
Fung-3375	Fung-17967	0.9398	0.001	Ascomycota	Ascomycota	Penicillium sp	Penicillium amphipolaria	positive	medium
Bac-114975	Bac-1569	0.9402	0.001	Firmicutes	Firmicutes	Sporosarcina aquimarina	Sporosarcina sp	positive	medium
Bac-452623	Bac-1002870	0.9415	0.001	Actinobacteria	Actinobacteria	Pseudoclavibacter soli	Gaiella occulta	positive	medium
Bac-1640866	Bac-588673	0.9425	0.001	Actinobacteria	Actinobacteria	Parviterribacter multiflagellatus	Unassigned	positive	medium
Bac-114975	Bac-1365606	0.9432	0.001	Firmicutes	Firmicutes	Sporosarcina aquimarina	Sporosarcina siberiensis	positive	medium
Bac-166793	Bac-207599	0.9458	0.001	Actinobacteria	Actinobacteria	Solirubrobacter pauli	Solirubrobacter sp	positive	medium
Bac-467091	Bac-1002870	0.9458	0.001	Actinobacteria	Actinobacteria	Ilumatobacter fluminis	Gaiella occulta	positive	medium
Bac-166793	Bac-363832	0.9471	0.001	Actinobacteria	Actinobacteria	Solirubrobacter pauli	Solirubrobacter soli	positive	medium
Bac-1002870	Bac-201174	0.9488	0.001	Actinobacteria	Actinobacteria	Gaiella occulta	Unassigned	positive	medium
Bac-1855912	Bac-1612172	0.9556	0.001	Acidobacteria	Acidobacteria	Luteitalea pratensis	Vicinamibacter silvestris	positive	medium
Bac-166793	Bac-1395955	0.9588	0.001	Actinobacteria	Actinobacteria	Solirubrobacter pauli	Solirubrobacter taibaiensis	positive	medium
Bac-363832	Bac-207599	0.9603	0.001	Actinobacteria	Actinobacteria	Solirubrobacter soli	Solirubrobacter sp	positive	medium
Fung-10239	Fung-2070	0.9665	0.001	Ascomycota	Ascomycota	Cladosporium cladosporioides	Cladosporium sp	positive	medium
Bac-1395955	Bac-207599	0.968	0.001	Actinobacteria	Actinobacteria	Solirubrobacter taibaiensis	Solirubrobacter sp	positive	medium
Bac-57003	Bac-57002	0.9687	0.001	Proteobacteria	Proteobacteria	Amaricoccus veronensis	Amaricoccus tamworthensis	positive	medium
Fung-3315	Fung-17177	0.9824	0.001	Ascomycota	Ascomycota	Exophiala sp	Exophiala tremulae	positive	medium
Fung-15976	Bac-1379270	-0.7687	0.001	Ascomycota	Gemmatimonadetes	Pleosporales sp	Gemmatimonas phototrophica	negative	high

Fung-15976	Bac-75309	-0.7579	0.001	Ascomycota	Proteobacteria	Pleosporales sp	Rhodanobacter sp	negative	high
Fung-16029	Bac-1740721	-0.7531	0.001	Ascomycota	Firmicutes	Pyrenochaetopsis tabarestanensis	Neobacillus cucumis	negative	high
Fung-35852	Bac-220684	-0.741	0.001	Ascomycota	Firmicutes	Hypocreales sp	Neobacillus drentensis	negative	high
Fung-17598	Bac-40544	-0.7403	0.001	Ascomycota	Proteobacteria	Aspergillus inflatus	Sutterella sp	negative	high
Fung-61184	Bac-1310416	-0.7297	0.001	Mortierellomycota	Bacteroidetes	Podila humilis	Ferruginibacter yonginensis	negative	high
Fung-36736	Bac-34072	-0.7283	0.001	Ascomycota	Proteobacteria	Neocosmospora falciformis	Variovorax sp	negative	high
Fung-15976	Bac-75310	-0.7266	0.001	Ascomycota	Proteobacteria	Pleosporales sp	Rhodanobacter lindaniclasticus	negative	high
Fung-15976	Bac-305976	-0.7251	0.001	Ascomycota	Proteobacteria	Pleosporales sp	Pusillimonas sp	negative	high
Fung-16029	Bac-220684	-0.7188	0.001	Ascomycota	Firmicutes	Pyrenochaetopsis tabarestanensis	Neobacillus drentensis	negative	high
Fung-61184	Bac-288995	-0.7105	0.001	Mortierellomycota	Proteobacteria	Podila humilis	Devosia limi	negative	high
Fung-36615	Bac-1310416	-0.7098	0.001	Ascomycota	Bacteroidetes	Ilyonectria destructans	Ferruginibacter yonginensis	negative	high
Fung-47054	Bac-2008795	-0.7086	0.001	Basidiomycota	Proteobacteria	Inocybe phaeoleuca	Unassigned	negative	high
Fung-1136	Bac-696763	0.7001	0.001	Ascomycota	Actinobacteria	Unassigned	Thermasporomyces composti	positive	high
Fung-19109	Bac-1221880	0.7006	0.001	Ascomycota	Firmicutes	Aphanoascus fulvescens	Psychrobacillus sp	positive	high
Fung-34944	Bac-85023	0.7025	0.001	Ascomycota	Actinobacteria	Marquandomyces marquandii	Unassigned	positive	high
Fung-1136	Bac-85023	0.7034	0.001	Ascomycota	Actinobacteria	Unassigned	Unassigned	positive	high

Fung-36615	Bac-696763	0.7043	0.001	Ascomycota	Actinobacteria	Ilyonectria destructans	Thermasporomyces composti	positive	high
Fung-15976	Bac-2789776	0.7148	0.001	Ascomycota	Actinobacteria	Pleosporales sp	Actinomarinicola tropica	positive	high
Fung-29945	Bac-57000	0.7175	0.001	Ascomycota	Proteobacteria	Geotrichum candidum	Amaricoccus kaplicensis	positive	high
Fung-13044	Bac-213115	0.7209	0.003	Ascomycota	Proteobacteria	Pyrenochaeta sp	Unassigned	positive	high
Fung-34569	Bac-2789776	0.7267	0.001	Ascomycota	Actinobacteria	Clonostachys sp	Actinomarinicola tropica	positive	high
Fung-10550	Bac-149698	0.7281	0.001	Ascomycota	Proteobacteria	Aureobasidium pullulans	Massilia sp	positive	high
Fung-34569	Bac-696763	0.7424	0.001	Ascomycota	Actinobacteria	Clonostachys sp	Thermasporomyces composti	positive	high
Fung-15976	Bac-467093	0.7772	0.001	Ascomycota	Actinobacteria	Pleosporales sp	Ilumatobacter nonamiensis	positive	high
Fung-10239	Fung-31	0.9007	0.001	Ascomycota	Ascomycota	Cladosporium cladosporioides	Unassigned	positive	high
Bac-1229157	Bac-1616821	0.9012	0.001	Firmicutes	Firmicutes	Tumebacillus soli	Tumebacillus luteolus	positive	high
Fung-8565	Fung-59728	0.9026	0.001	Basidiomycota	Basidiomycota	Vishniacozyma sp	Vishniacozyma sp	positive	high
Bac-1474	Bac-1365606	0.9033	0.001	Firmicutes	Firmicutes	Sporosarcina pasteurii	Sporosarcina siberiensis	positive	high
Fung-59990	Fung-8598	0.9048	0.001	Basidiomycota	Basidiomycota	Saitozyma podzolica	Saitozyma sp	positive	high
Fung-18138	Fung-18059	0.9056	0.001	Ascomycota	Ascomycota	Penicillium fundyense	Penicillium citrinum	positive	high
Bac-1474	Bac-1467	0.9062	0.001	Firmicutes	Firmicutes	Sporosarcina pasteurii	Lederbergia lentus	positive	high
Bac-57003	Bac-57002	0.9069	0.001	Proteobacteria	Proteobacteria	Amaricoccus veronensis	Amaricoccus tamworthensis	positive	high
Bac-582850	Bac-1467	0.9085	0.001	Firmicutes	Firmicutes	Sporosarcina luteola	Lederbergia lentus	positive	high
Bac-166793	Bac-363832	0.9089	0.001	Actinobacteria	Actinobacteria	Solirubrobacter pauli	Solirubrobacter soli	positive	high
Fung-8550	Fung-59579	0.9093	0.001	Basidiomycota	Basidiomycota	Solicoccozyma sp	Solicoccozyma gelidoterrea	positive	high
Bac-363832	Bac-207599	0.9104	0.001	Actinobacteria	Actinobacteria	Solirubrobacter soli	Solirubrobacter sp	positive	high
Fung-8544	Fung-59544	0.9109	0.001	Basidiomycota	Basidiomycota	Naganishia sp	Naganishia brisbanensis	positive	high

Bac-1365606	Bac-1569	0.9134	0.001	Firmicutes	Firmicutes	Sporosarcina siberiensis	Sporosarcina sp	positive	high
Fung-35747	Fung-5988	0.9156	0.001	Ascomycota	Ascomycota	Trichoderma sp	Trichoderma sp	positive	high
Bac-1855912	Bac-1612172	0.9165	0.001	Acidobacteria	Acidobacteria	Luteitalea pratensis	Vicinamibacter silvestris	positive	high
Fung-769	Fung-3315	0.9167	0.001	Ascomycota	Ascomycota	Unassigned	Exophiala sp	positive	high
Bac-1474	Bac-1569	0.9187	0.001	Firmicutes	Firmicutes	Sporosarcina pasteurii	Sporosarcina sp	positive	high
Bac-1474	Bac-582850	0.923	0.001	Firmicutes	Firmicutes	Sporosarcina pasteurii	Sporosarcina luteola	positive	high
Bac-582850	Bac-1569	0.9302	0.001	Firmicutes	Firmicutes	Sporosarcina luteola	Sporosarcina sp	positive	high
Bac-114975	Bac-1569	0.9308	0.001	Firmicutes	Firmicutes	Sporosarcina aquimarina	Sporosarcina sp	positive	high
Bac-582850	Bac-1365606	0.9331	0.001	Firmicutes	Firmicutes	Sporosarcina luteola	Sporosarcina siberiensis	positive	high
Fung-36424	Fung-49	0.9335	0.001	Ascomycota	Ascomycota	Fusarium oxysporum	Unassigned	positive	high
Fung-5220	Fung-29770	0.9352	0.001	Ascomycota	Ascomycota	Debaryomyces sp	Debaryomyces hansenii	positive	high
Fung-769	Fung-17177	0.9369	0.001	Ascomycota	Ascomycota	Unassigned	Exophiala tremulae	positive	high
Fung-18138	Fung-18058	0.9392	0.001	Ascomycota	Ascomycota	Penicillium fundyense	Penicillium citreosulfuratum	positive	high
Bac-57003	Bac-56999	0.9408	0.001	Proteobacteria	Proteobacteria	Amaricoccus veronensis	Amaricoccus sp	positive	high
Bac-114975	Bac-582850	0.9433	0.001	Firmicutes	Firmicutes	Sporosarcina aquimarina	Sporosarcina luteola	positive	high
Fung-10239	Fung-2070	0.9442	0.001	Ascomycota	Ascomycota	Cladosporium cladosporioides	Cladosporium sp	positive	high
Bac-1803212	Bac-1474	0.9472	0.001	Firmicutes	Firmicutes	Sporosarcina terrae	Sporosarcina pasteurii	positive	high
Fung-8565	Fung-59731	0.954	0.001	Basidiomycota	Basidiomycota	Vishniacozyma sp	Vishniacozyma victoriae	positive	high
Bac-114975	Bac-1365606	0.9553	0.001	Firmicutes	Firmicutes	Sporosarcina aquimarina	Sporosarcina siberiensis	positive	high
Fung-3315	Fung-17177	0.9732	0.001	Ascomycota	Ascomycota	Exophiala sp	Exophiala tremulae	positive	high

Supplementary Table 5

asv1	asv2	cor	pval	phlum_1	phlum_2	species_1	species_2	guild_1	guild_2	type_interaction	lichen
Bac-888829	Bac-1806202	-0.9193	0.001	Proteobacteria	Bacteroidetes	Aetherobacter sp	Spirosoma areae	unknown_unknown	unknown_unknown	negative	Candelaria concolor
Fung-4	Bac-57002	-0.8927	0.001	Ascomycota	Proteobacteria	Unassigned	Amaricoccus tamworthensis	unknown_unknown	aerobic chemoheterotrophy_unknown	negative	Candelaria concolor
Fung-24903	Bac-534635	-0.8901	0.003	Ascomycota	Bacteroidetes	Xanthoria parietina	Mucilaginibacter rigui	lichenized_unknown	aerobic chemoheterotroph_cellulolysis	negative	Candelaria concolor
Fung-49	Bac-33882	-0.8773	0.001	Ascomycota	Actinobacteria	Unassigned	Microbacterium sp	unknown_unknown	unknown_unknown	negative	Candelaria concolor
Fung-2244	Bac-1763824	-0.8729	0.001	Ascomycota	Proteobacteria	Arxiella sp	Sphingomonas aquatica	litter saprotroph_unknown	aerobic chemoheterotrophy_unknown	negative	Candelaria concolor
Fung-9385	Bac-582672	-0.8666	0.001	Ascomycota	Proteobacteria	Xylohypha sp	Methylobacterium phyllostachyos	wood saprotroph_unknown	methylotrophy_methanol oxidation	negative	Candelaria concolor
Fung-49	Bac-85021	-0.8595	0.001	Ascomycota	Actinobacteria	Unassigned	Unassigned	unknown_unknown	unknown_unknown	negative	Candelaria concolor
Fung-12647	Bac-582672	-0.8576	0.001	Ascomycota	Proteobacteria	Myriangiaceae Myriangiaceae_sp	Methylobacterium phyllostachyos	unknown_unknown	methylotrophy_methanol oxidation	negative	Candelaria concolor
Fung-59981	Bac-582672	-0.8527	0.001	Basidiomycota	Proteobacteria	Tremellales Tremellales_sp	Methylobacterium phyllostachyos	unknown_unknown	methylotrophy_methanol oxidation	negative	Candelaria concolor

Fung-59849	Bac-653931	-0.8468	0.004	Basidiomycota	Proteobacteria	Papiliotrema flavescens	Sphingomonas alpina	mycoparasite_unknown	aerobic chemoheterotrophy_unknown	negative	Candelaria concolor
Fung-15065	Bac-888830	-0.8437	0.002	Ascomycota	Proteobacteria	Phaeosphaeriaceae Phaeosphaeriaceae_sp	Aetherobacter fasciculatus	unknown_unknown	unknown_unknown	negative	Candelaria concolor
Fung-292	Bac-1983306	-0.8426	0.001	Ascomycota	Bacteroidetes	Unassigned	Edaphobaculum flavum	unknown_unknown	unknown_unknown	negative	Candelaria concolor
Fung-17303	Bac-1827279	-0.8416	0.006	Ascomycota	Cyanobacteria	Knufia sp	Aliterella antarctica	soil saprotroph_unknown	oxygenic photoautotroph_unknown	negative	Candelaria concolor
Fung-4	Bac-80864	-0.8414	0.001	Ascomycota	Proteobacteria	Unassigned	Unassigned	unknown_unknown	unknown_unknown	negative	Candelaria concolor
Fung-3674	Bac-80816	-0.8369	0.001	Ascomycota	Proteobacteria	Phaeophyscia sp	Haliangium ochraceum	lichenized_unknown	unknown_unknown	negative	Candelaria concolor
Fung-17338	Bac-1640865	-0.8362	0.002	Ascomycota	Actinobacteria	Trichomerium sp	Parviterribacter kavangonensis	foliar endophyte_unknown	aerobic chemoheterotrophy_unknown	negative	Candelaria concolor
Fung-59849	Bac-119045	-0.8356	0.001	Basidiomycota	Proteobacteria	Papiliotrema flavescens	Unassigned	mycoparasite_unknown	unknown_unknown	negative	Candelaria concolor
Fung-59849	Bac-373043	-0.8339	0.001	Basidiomycota	Proteobacteria	Papiliotrema flavescens	Roseomonas aquatica	mycoparasite_unknown	animal parasites or symbionts_ureolysis	negative	Candelaria concolor
Fung-9385	Bac-1005039	-0.8335	0.001	Ascomycota	Armatimonadetes	Xylohypha sp	Fimbriimonas ginsengisoli	wood saprotroph_unknown	unknown_unknown	negative	Candelaria concolor
Fung-292	Bac-1055355	-0.8	0.002	Ascomycota	Proteobacteria	Unassigned	Belnapia soli	unknown_unknown	unknown_unknown	negative	Candelaria concolor

		323								
Fung-2244	Bac-1235480	-0.8311	0.002	Ascomycota	Acidobacteria	Arxiella sp	Blastocatella fastidiosa	litter saprotroph_unknown	aerobic chemoheterotrophy_cellulolysis	negative	Candelaria concolor
Fung-22759	Bac-582672	-0.8306	0.002	Ascomycota	Proteobacteria	Bacidina sp	Methylobacterium phyllostachyos	lichenized_unknown	methylotrophy_methanol oxidation	negative	Candelaria concolor
Fung-22759	Bac-107	-0.8279	0.001	Ascomycota	Bacteroidetes	Bacidina sp	Spirosoma sp	lichenized_unknown	unknown_unknown	negative	Candelaria concolor
Fung-59849	Bac-1763824	-0.8258	0.001	Basidiomycota	Proteobacteria	Papiliotrema flavescens	Sphingomonas aquatica	mycoparasite_unknown	aerobic chemoheterotrophy_unknown	negative	Candelaria concolor
Fung-20430	Bac-1752142	-0.8256	0.002	Ascomycota	Proteobacteria	Physcia dubia	Paraburkholderia pallidirosea	lichenized_unknown	unknown_unknown	negative	Candelaria concolor
Fung-4	Bac-1189325	-0.8238	0.003	Ascomycota	Proteobacteria	Unassigned	Oceanicella actignis	unknown_unknown	unknown_unknown	negative	Candelaria concolor
Fung-22759	Bac-767694	-0.8227	0.001	Ascomycota	Proteobacteria	Bacidina sp	Methylobacterium longum	lichenized_unknown	methylotrophy_methanol oxidation	negative	Candelaria concolor
Fung-15103	Bac-1987378	-0.8215	0.001	Ascomycota	Bacteroidetes	Sclerostagonospora cycadis	Spirosoma jeollabukense	litter saprotroph_unknown	unknown_unknown	negative	Candelaria concolor
Fung-9430	Bac-2784132	-0.8212	0.002	Ascomycota	Cyanobacteria	Candelariella xanthostigmoides	Leptodesmis alaskaensis	lichenized_unknown	oxygenic photoautotroph_unknown	negative	Candelaria concolor
Fung-36668	Bac-119060	-0.8188	0.001	Ascomycota	Proteobacteria	Microcera physciae	Unassigned	animal parasite_unknown	unknown_unknown	negative	Candelaria concolor

Fung-9385	Bac-373043	-0.8185	0.002	Ascomycota	Proteobacteria	Xylohypha sp	Roseomonas aquatica	wood saprotroph_unknown	animal parasites or symbionts_ureolysis	negative	Candelaria concolor
Fung-59849	Bac-582672	-0.8169	0.002	Basidiomycota	Proteobacteria	Papiliotrema flavescens	Methylobacterium phyllostachyos	mycoparasite_unknown	methylotrophy_methanol oxidation	negative	Candelaria concolor
Fung-10635	Bac-1434046	-0.8163	0.005	Ascomycota	Proteobacteria	Oncopodiella sp	Sphingorhabdus sp	woodsaprotroph_unknown	unknown_unknown	negative	Candelaria concolor
Fung-59849	Bac-407	-0.8159	0.001	Basidiomycota	Proteobacteria	Papiliotrema flavescens	Methylobacterium sp	mycoparasite_unknown	unknown_unknown	negative	Candelaria concolor
Fung-12647	Bac-512383	-0.8158	0.002	Ascomycota	Proteobacteria	Myriangiaceae Myriangiaceae_sp	Acidisoma sibiricum	unknown_unknown	aerobic chemoheterotrophy_unknown	negative	Candelaria concolor
Fung-4	Bac-1861874	-0.8153	0.003	Ascomycota	Proteobacteria	Unassigned	Sphingomonas gotjawalisoli	unknown_unknown	aerobic chemoheterotrophy_unknown	negative	Candelaria concolor
Fung-49	Bac-88374	-0.8151	0.002	Ascomycota	Actinobacteria	Unassigned	Agromyces rhizosphaerae	unknown_unknown	aerobic chemoheterotrophy_unknown	negative	Candelaria concolor
Fung-58762	Bac-1452450	-0.8151	0.003	Basidiomycota	Proteobacteria	Chrysozymaceae Chrysozymaceae_sp	Roseomonas alkaliterrae	unknown_unknown	animal parasite or symbionts_unknown	negative	Candelaria concolor
Fung-22057	Bac-418707	-0.8144	0.003	Ascomycota	Proteobacteria	Parmelia encryptata	Methylobacterium iners	lichenized_unknown	methylotrophy_methanol oxidation	negative	Candelaria concolor
Fung-809	Bac-888829	-0.8132	0.002	Ascomycota	Proteobacteria	Unassigned	Aetherobacter sp	unknown_unknown	unknown_unknown	negative	Candelaria concolor
Fung-22739	Bac-418707	-0.8	0.002	Ascomycota	Proteobacteria	Bacidia sp	Methylobacterium iners	lichenized_unknown	methylotrophy_methanol oxidation	negative	Candelaria concolor

		122									
Fung-45033	Bac-932741	-0.8119	0.002	Basidiomycota	Proteobacteria	Clitopilus sp	Methylobacterium cerastii	litter saprotroph_unknown	methylotrophy_methanol oxidation	negative	Candelaria concolor
Fung-59981	Bac-373043	-0.8116	0.001	Basidiomycota	Proteobacteria	Tremellales Tremellales_sp	Roseomonas aquatica	unknown_unknown	animal parasites or symbionts_ureolysis	negative	Candelaria concolor
Fung-11276	Bac-2802093	-0.8109	0.001	Ascomycota	Cyanobacteria	Mycosphaerellaceae Mycosphaerellaceae_sp	Aliinostoc catenatum	unknown_unknown	oxygenic photoautotroph_nitrogen fixation	negative	Candelaria concolor
Fung-1146	Bac-1310416	-0.8093	0.002	Ascomycota	Bacteroidetes	Unassigned	Ferruginibacter yonginensis	unknown_unknown	aerobic chemoheterotrophy_unknown	negative	Candelaria concolor
Fung-2244	Bac-373043	-0.8092	0.002	Ascomycota	Proteobacteria	Arxiella sp	Roseomonas aquatica	litter saprotroph_unknown	animal parasites or symbionts_ureolysis	negative	Candelaria concolor
Fung-21408	Bac-317664	-0.8091	0.003	Ascomycota	Actinobacteria	Lecidella elaeochroma	Quadrisphaera granulorum	lichenized_unknown	unknown_unknown	negative	Candelaria concolor
Fung-20174	Bac-534635	-0.8091	0.003	Ascomycota	Bacteroidetes	Amandinea punctata	Mucilaginibacter rigui	lichenized_unknown	aerobic chemoheterotroph_cellulolysis	negative	Candelaria concolor
Fung-59731	Bac-191863	-0.8085	0.002	Basidiomycota	Verrucomicrobia	Vishniacozyma victoriae	Chthoniobacter flavus	soil saprotroph_unknown	aerobic chemoheterotrophy_unknown	negative	Candelaria concolor
Fung-24994	Bac-888830	-0.8078	0.003	Ascomycota	Proteobacteria	Umbilicaria esculenta	Aetherobacter fasciculatus	lichenized_unknown	unknown_unknown	negative	Candelaria concolor
Fung-37	Bac-888829	-0.8078	0.002	Ascomycota	Proteobacteria	Unassigned	Aetherobacter sp	unknown_unknown	unknown_unknown	negative	Candelaria concolor

Fung-37237	Bac-41297	-0.8077	0.001	Ascomycota	Proteobacteria	Tolypocladium sp	Unassigned	animal parasite_unknown	unknown_unknown	negative	Candelaria concolor
Fung-4	Bac-2678302	-0.8072	0.003	Ascomycota	Bacteroidetes	Unassigned	Mucibacter soli	unknown_unknown	aerobic chemoheterotroph_cellulolysis	negative	Candelaria concolor
Fung-4	Bac-2040274	-0.8072	0.003	Ascomycota	Proteobacteria	Unassigned	Sphingomonas antarctica	unknown_unknown	aerobic chemoheterotrophy_unknown	negative	Candelaria concolor
Fung-59849	Bac-1278057	-0.8064	0.002	Basidiomycota	Bacteroidetes	Papiliotrema flavescens	Hymenobacter marinus	mycoparasite_unknown	unknown_unknown	negative	Candelaria concolor
Fung-59981	Bac-767694	-0.8059	0.005	Basidiomycota	Proteobacteria	Tremellales Tremellales_sp	Methylobacterium longum	unknown_unknown	methylotrophy_methanol oxidation	negative	Candelaria concolor
Fung-17304	Bac-1452450	-0.8055	0.001	Ascomycota	Proteobacteria	Knufia tsunedae	Roseomonas alkaliterrae	soil saprotroph_unknown	animal parasite or symbionts_unknown	negative	Candelaria concolor
Fung-59849	Bac-107	-0.8051	0.002	Basidiomycota	Bacteroidetes	Papiliotrema flavescens	Spirosoma sp	mycoparasite_unknown	unknown_unknown	negative	Candelaria concolor
Fung-22057	Bac-223967	-0.8044	0.001	Ascomycota	Proteobacteria	Parmelia encryptata	Methylorubrum populi	lichenized_unknown	unknown_unknown	negative	Candelaria concolor
Fung-9393	Bac-662367	-0.8037	0.002	Ascomycota	Bacteroidetes	Candelaria pacifica	Spirosoma endophyticum	lichenized_unknown	unknown_unknown	negative	Candelaria concolor
Fung-22739	Bac-767694	-0.8037	0.001	Ascomycota	Proteobacteria	Bacidia sp	Methylobacterium longum	lichenized_unknown	methylotrophy_methanol oxidation	negative	Candelaria concolor
Fung-58762	Bac-1395955	-0.8	0.004	Basidiomycota	Actinobacteria	Chrysozymaceae Chrysozymaceae_sp	Solirubrobacter taibaiensis	unknown_unknown	aerobic chemoheterotrophy_unknown	negative	Candelaria concolor

		009									
Fung-9387	Bac-2799740	0.8001	0.006	Ascomycota	Cyanobacteria	Candelaria concolor	Aegeococcus thureti	lichenized_unknown	oxygenic photoautotroph_unknown	positive	Candelaria concolor
Fung-37	Bac-364410	0.8002	0.002	Ascomycota	Proteobacteria	Unassigned	Granulibacter bethesdensis	unknown_unknown	unknown_unknown	positive	Candelaria concolor
Fung-10239	Bac-1792845	0.8005	0.003	Ascomycota	Bacteroidetes	Cladosporium cladosporioides	Mucilaginibacter pedocola	litter saprotroph_unknown	aerobic chemoheterotroph_cellulolysis	positive	Candelaria concolor
Fung-21933	Bac-2048557	0.8011	0.003	Ascomycota	Bacteroidetes	Melanelixia subaurifera	Spirosoma flavum	lichenized_unknown	unknown_unknown	positive	Candelaria concolor
Fung-2881	Bac-1815558	0.802	0.001	Ascomycota	Bacteroidetes	Nigrograna sp	Mucilaginibacter amnicola	animal parasite_unknown	unknown_unknown	positive	Candelaria concolor
Fung-17271	Bac-468913	0.8022	0.001	Ascomycota	Deinococcus-Thermus	Neophaeococcomyces catenatus	Deinococcus alpinitundrae	plant pathogen_unknown	unknown_unknown	positive	Candelaria concolor
Fung-24903	Bac-1975704	0.8036	0.001	Ascomycota	Proteobacteria	Xanthoria parietina	Sphingomonas frigidaeris	lichenized_unknown	aerobic chemoheterotrophy_unknown	positive	Candelaria concolor
Fung-17338	Bac-940613	0.804	0.006	Ascomycota	Acidobacteria	Trichomerium sp	Granulicella arctica	foliar endophyte_unknown	aerobic chemoheterotrophy_unknown	positive	Candelaria concolor
Fung-292	Bac-1603353	0.8046	0.002	Ascomycota	Proteobacteria	Unassigned	Massilia psychrophila	unknown_unknown	unknown_unknown	positive	Candelaria concolor
Fung-15976	Bac-2219509	0.8048	0.004	Ascomycota	Cyanobacteria	Pleosporales Pleosporales_sp	Haloleptolyngbya elongata	unknown_unknown	oxygenic photoautotroph_unknown	positive	Candelaria concolor
Fung-20386	Bac-1983306	0.8068	0.002	Ascomycota	Bacteroidetes	Phaeophyscia ciliata	Edaphobaculum flavum	lichenized_unknown	unknown_unknown	positive	Candelaria concolor
Fung-24994	Bac-708199	0.8084	0.002	Ascomycota	Bacteroidetes	Umbilicaria esculenta	Flavitalea gansuensis	lichenized_unknown	unknown_unknown	positive	Candelaria concolor
Fung-26839	Bac-36862	0.8087	0.004	Ascomycota	Proteobacteria	Calycina sp	Ideonella sp	wood saprotroph_unknown	unknown_unknown	positive	Candelaria concolor

Fung-16981	Bac-2784132	0.809	0.003	Ascomycota	Cyanobacteria	Chaetothyriales Chaetothyriales_sp	Leptodesmis alaskaensis	unknown_unknown	oxygenic photoautotroph_nitrogen fixation	positive	Candelaria concolor
Fung-12209	Bac-468913	0.8094	0.001	Ascomycota	Deinococcus-Thermus	Neodevriesia sp	Deinococcus alpinitundrae	plant pathogen_unknown	unknown_unknown	positive	Candelaria concolor
Fung-22759	Bac-36862	0.8118	0.001	Ascomycota	Proteobacteria	Bacidina sp	Ideonella sp	lichenized_unknown	unknown_unknown	positive	Candelaria concolor
Fung-15976	Bac-2831758	0.812	0.004	Ascomycota	Cyanobacteria	Pleosporales Pleosporales_sp	Unassigned	unknown_unknown	unknown_unknown	positive	Candelaria concolor
Fung-12647	Bac-36862	0.8137	0.001	Ascomycota	Proteobacteria	Myriangiaceae Myriangiaceae_sp	Ideonella sp	unknown_unknown	unknown_unknown	positive	Candelaria concolor
Fung-15976	Bac-693994	0.8138	0.005	Ascomycota	Cyanobacteria	Pleosporales Pleosporales_sp	Pseudoscillatoria coralii	unknown_unknown	oxygenic photoautotroph_nitrogen fixation	positive	Candelaria concolor
Fung-809	Bac-209650	0.8143	0.002	Ascomycota	Proteobacteria	Unassigned	Acidicaldus organivorans	unknown_unknown	aerobic chemoheterotrophy_iron reduction	positive	Candelaria concolor
Fung-59849	Bac-36862	0.8155	0.003	Basidiomycota	Proteobacteria	Papiliotrema flavescens	Ideonella sp	mycoparasite_unknown	unknown_unknown	positive	Candelaria concolor
Fung-21748	Bac-1150	0.8156	0.002	Ascomycota	Cyanobacteria	Flavopunctelia flaventior	Unassigned	lichenized_unknown	unknown_unknown	positive	Candelaria concolor
Fung-10115	Bac-889274	0.8158	0.002	Ascomycota	Proteobacteria	Chaetocapnodium sp	Polyangium sorediatum	sooty mold_unknown	unknown_unknown	positive	Candelaria concolor
Fung-17303	Bac-2831758	0.8166	0.009	Ascomycota	Cyanobacteria	Knufia sp	Unassigned	soil saprotroph_unknown	unknown_unknown	positive	Candelaria concolor
Fung-20386	Bac-582672	0.8167	0.003	Ascomycota	Proteobacteria	Phaeophyscia ciliata	Methylobacterium phyllostachyos	lichenized_unknown	methylotrophy_methanol oxidation	positive	Candelaria concolor
Fung-17303	Bac-693994	0.8173	0.009	Ascomycota	Cyanobacteria	Knufia sp	Pseudoscillatoria coralii	soil saprotroph_unknown	oxygenic photoautotroph_nitrogen fixation	positive	Candelaria concolor
Fung-17303	Bac-1117	0.8179	0.008	Ascomycota	Cyanobacteria	Knufia sp	Unassigned	soil saprotroph_unknown	unknown_unknown	positive	Candelaria concolor

Fung-12209	Bac-944322	0.8183	0.001	Ascomycota	Proteobacteria	Neodevriesia sp	Methylobacterium oxalidis	plant pathogen_unknown	methylotrophy_methanol oxidation	positive	Candelaria concolor
Fung-20366	Bac-468916	0.8192	0.002	Ascomycota	Deinococcus-Thermus	Hyperphyscia adglutinata	Deinococcus radiomollis	lichenized_unknown	unknown_unknown	positive	Candelaria concolor
Fung-2881	Bac-423349	0.8214	0.001	Ascomycota	Bacteroidetes	Nigrograna sp	Mucilaginibacter sp	animal parasite_unknown	aerobic chemoheterotroph_cellulolysis	positive	Candelaria concolor
Fung-576	Bac-889274	0.8217	0.003	Ascomycota	Proteobacteria	Unassigned	Polyangium sorediatum	unknown_unknown	unknown_unknown	positive	Candelaria concolor
Fung-20366	Bac-1031594	0.8218	0.002	Ascomycota	Proteobacteria	Hyperphyscia adglutinata	Extensimonas vulgaris	lichenized_unknown	unknown_unknown	positive	Candelaria concolor
Fung-11276	Bac-889274	0.8222	0.001	Ascomycota	Proteobacteria	Mycosphaerellaceae Mycosphaerellaceae_sp	Polyangium sorediatum	unknown_unknown	unknown_unknown	positive	Candelaria concolor
Fung-26839	Bac-2048557	0.8232	0.002	Ascomycota	Bacteroidetes	Calycina sp	Spirosoma flavum	wood saprotroph_unknown	unknown_unknown	positive	Candelaria concolor
Fung-26723	Bac-2881377	0.8233	0.001	Ascomycota	Cyanobacteria	Phialocephala fortinii	Unassigned	soil saprotroph_unknown	unknown_unknown	positive	Candelaria concolor
Fung-20366	Bac-1987378	0.8237	0.003	Ascomycota	Bacteroidetes	Hyperphyscia adglutinata	Spirosoma jeollabukense	lichenized_unknown	unknown_unknown	positive	Candelaria concolor
Fung-2244	Bac-286	0.8243	0.001	Ascomycota	Proteobacteria	Arxiella sp	Pseudomonas sp	litter saprotroph_unknown	unknown_unknown	positive	Candelaria concolor
Fung-26723	Bac-1458934	0.8267	0.002	Ascomycota	Cyanobacteria	Phialocephala fortinii	Okeania lorea	soil saprotroph_unknown	oxygenic photoautotroph_unknown	positive	Candelaria concolor
Fung-12314	Bac-1849581	0.8267	0.002	Ascomycota	Proteobacteria	Devriesia sp	Dyella caseinilytica	plant pathogen_unknown	unknown_unknown	positive	Candelaria concolor
Fung-15976	Bac-1117	0.8289	0.004	Ascomycota	Cyanobacteria	Pleosporales Pleosporales_sp	Unassigned	unknown_unknown	unknown_unknown	positive	Candelaria concolor
Fung-59849	Bac-1603353	0.8326	0.002	Basidiomycota	Proteobacteria	Papiliotrema flavescens	Massilia psychrophila	mycoparasite_unknown	unknown_unknown	positive	Candelaria concolor

Fung-14633	Bac-470868	0.8332	0.001	Ascomycota	Proteobacteria	Nigrograna sp	Achromobacter marplatensis	animal parasite_unknown	aerobic chemoheterotrophy_denitrification	positive	Candelaria concolor
Fung-12314	Bac-540746	0.8357	0.001	Ascomycota	Proteobacteria	Devriesia sp	Rudaea cellulosilytica	plant pathogen_unknown	unknown_unknown	positive	Candelaria concolor
Fung-23143	Bac-651143	0.836	0.001	Ascomycota	Bacteroidetes	Scoliciosporum fabisporum	Fibrella aestuarina	lichenized_unknown	unknown_unknown	positive	Candelaria concolor
Fung-20386	Bac-1760	0.8379	0.001	Ascomycota	Actinobacteria	Phaeophyscia ciliata	Unassigned	lichenized_unknown	unknown_unknown	positive	Candelaria concolor
Fung-9387	Bac-2881377	0.838	0.007	Ascomycota	Cyanobacteria	Candelaria concolor	Unassigned	lichenized_unknown	unknown_unknown	positive	Candelaria concolor
Fung-15976	Bac-1126	0.8397	0.002	Ascomycota	Cyanobacteria	Pleosporales Pleosporales_sp	Microcystis aeruginosa	unknown_unknown	oxygenic photoautotroph_harmful algal blooms	positive	Candelaria concolor
Fung-17265	Bac-1252216	0.8435	0.002	Ascomycota	Proteobacteria	Veronaea sp	Roseomonas sp	plant pathogen_unknown	unknown_unknown	positive	Candelaria concolor
Fung-17338	Bac-1560005	0.8493	0.003	Ascomycota	Acidobacteria	Trichomerium sp	Edaphobacter dinghuensis	foliar endophyte_unknown	aerobic chemoheterotrophy_unknown	positive	Candelaria concolor
Fung-9387	Bac-2784132	0.8512	0.007	Ascomycota	Cyanobacteria	Candelaria concolor	Leptodesmis alaskaensis	lichenized_unknown	oxygenic photoautotroph_nitrogen fixation	positive	Candelaria concolor
Fung-26723	Bac-1117	0.8513	0.004	Ascomycota	Cyanobacteria	Phialocephala fortinii	Unassigned	soil saprotroph_unknown	unknown_unknown	positive	Candelaria concolor
Fung-22057	Bac-363843	0.8518	0.002	Ascomycota	Proteobacteria	Parmelia encryptata	Rhizobacter fulvus	lichenized_unknown	unknown_unknown	positive	Candelaria concolor
Fung-9387	Bac-2831758	0.8557	0.009	Ascomycota	Cyanobacteria	Candelaria concolor	Unassigned	lichenized_unknown	unknown_unknown	positive	Candelaria concolor
Fung-26723	Bac-1126	0.8561	0.002	Ascomycota	Cyanobacteria	Phialocephala fortinii	Microcystis aeruginosa	soil saprotroph_unknown	oxygenic photoautotroph_harmful algal blooms	positive	Candelaria concolor
Fung-26723	Bac-2831758	0.8588	0.002	Ascomycota	Cyanobacteria	Phialocephala fortinii	Unassigned	soil saprotroph_unknown	unknown_unknown	positive	Candelaria concolor

Fung-809	Bac-1806202	0.8593	0.001	Ascomycota	Bacteroidetes	Unassigned	Spirosoma areae	unknown_unknown	unknown_unknown	positive	Candelaria concolor
Fung-26723	Bac-693994	0.8617	0.004	Ascomycota	Cyanobacteria	Phialocephala fortinii	Pseudoscillatoria coralii	soil saprotroph_unknown	oxygenic photoautotroph_nitrogen fixation	positive	Candelaria concolor
Fung-24994	Bac-119045	0.8633	0.001	Ascomycota	Proteobacteria	Umbilicaria esculenta	Unassigned	lichenized_unknown	unknown_unknown	positive	Candelaria concolor
Fung-22212	Bac-39956	0.865	0.001	Ascomycota	Proteobacteria	Punctelia borreri	Methylobacterium mesophilicum	lichenized_unknown	methylotrophy_methanol oxidation	positive	Candelaria concolor
Fung-9387	Bac-1126	0.8662	0.006	Ascomycota	Cyanobacteria	Candelaria concolor	Microcystis aeruginosa	lichenized_unknown	oxygenic photoautotroph_harmful algal blooms	positive	Candelaria concolor
Fung-12210	Bac-944322	0.8664	0.001	Ascomycota	Proteobacteria	Neodevriesia stirlingiae	Methylobacterium oxalidis	plant pathogen_unknown	methylotrophy_methanol oxidation	positive	Candelaria concolor
Fung-26723	Bac-2219509	0.8666	0.002	Ascomycota	Cyanobacteria	Phialocephala fortinii	Haloleptolyngbya elongata	soil saprotroph_unknown	oxygenic photoautotroph_unknown	positive	Candelaria concolor
Fung-10636	Bac-2784132	0.8957	0.002	Ascomycota	Cyanobacteria	Oncopodiella trigonella	Leptodesmis alaskaensis	wood saprotroph_unknown	oxygenic photoautotroph_nitrogen fixation	positive	Candelaria concolor
Bac-582672	Bac-373043	0.9002	0.001	Proteobacteria	Proteobacteria	Methylobacterium phyllostachyos	Roseomonas aquatica	methylotrophy_methanol oxidation	animal parasites or symbionts_ureolysis	positive	Candelaria concolor
Bac-1126	Bac-2799740	0.9017	0.001	Cyanobacteria	Cyanobacteria	Microcystis aeruginosa	Aegeococcus thureti	oxygenic photoautotroph_harmful algal blooms	oxygenic photoautotroph_unknown	positive	Candelaria concolor
Bac-2784132	Bac-2219509	0.9018	0.006	Cyanobacteria	Cyanobacteria	Leptodesmis alaskaensis	Haloleptolyngbya elongata	oxygenic photoautotroph_unknown	oxygenic photoautotroph_unknown	positive	Candelaria concolor
Bac-1117	Bac-1458934	0.9029	0.001	Cyanobacteria	Cyanobacteria	Unassigned	Okeania lorea	unknown_unknown	oxygenic photoautotroph_unknown	positive	Candelaria concolor
Bac-2219509	Bac-2799740	0.9031	0.002	Cyanobacteria	Cyanobacteria	Haloleptolyngbya elongata	Aegeococcus thureti	oxygenic photoautotroph_unknown	oxygenic photoautotroph_unknown	positive	Candelaria concolor
Fung-12210	Fung-12209	0.9032	0.001	Ascomycota	Ascomycota	Neodevriesia stirlingiae	Neodevriesia sp	plant pathogen_unknown	plant pathogen_unknown	positive	Candelaria concolor

Bac-2219509	Bac-1458934	0.9059	0.002	Cyanobacteria	Cyanobacteria	Haloleptolyngbya elongata	Okeania lorea	oxygenic photoautotroph_unknown	oxygenic photoautotroph_unknown	positive	Candelaria concolor
Bac-1458934	Bac-693994	0.9066	0.001	Cyanobacteria	Cyanobacteria	Okeania lorea	Pseudoscillatoria coralii	oxygenic photoautotroph_unknown	oxygenic photoautotroph_nitrogen fixation	positive	Candelaria concolor
Bac-270351	Bac-1278057	0.9076	0.001	Proteobacteria	Bacteroidetes	Methylobacterium aquaticum	Hymenobacter marinus	methylotrophy_methanol oxidation	unknown_unknown	positive	Candelaria concolor
Fung-37237	Fung-37205	0.9081	0.001	Ascomycota	Ascomycota	Tolypocladium sp	Purpureocillium lilacinum	animal parasite_unknown	animal parasite_unknown	positive	Candelaria concolor
Bac-693994	Bac-2799740	0.9105	0.001	Cyanobacteria	Cyanobacteria	Pseudoscillatoria coralii	Aegeococcus thureti	oxygenic photoautotroph_nitrogen fixation	oxygenic photoautotroph_unknown	positive	Candelaria concolor
Fung-10144	Fung-577	0.9107	0.001	Ascomycota	Ascomycota	Arthrocatena sp	Unassigned	soil saprotroph_unknown	unknown_unknown	positive	Candelaria concolor
Bac-32056	Bac-1246630	0.9115	0.002	Cyanobacteria	Cyanobacteria	Calothrix desertica	Aerosakkonema funiforme	oxygenic photoautotroph_nitrogen fixation	oxygenic photoautotroph_nitrogen fixation	positive	Candelaria concolor
Fung-37237	Fung-1150	0.913	0.001	Ascomycota	Ascomycota	Tolypocladium sp	Unassigned	animal parasite_unknown	unknown_unknown	positive	Candelaria concolor
Fung-17304	Fung-3337	0.9131	0.001	Ascomycota	Ascomycota	Knufia tsunedae	Knufia sp	soil saprotroph_unknown	soil saprotroph_unknown	positive	Candelaria concolor
Fung-196	Fung-17178	0.9136	0.001	Ascomycota	Ascomycota	Unassigned	Exophiala xenobiotica	unknown_unknown	animal parasite_unknown	positive	Candelaria concolor
Bac-1004	Bac-317664	0.9154	0.001	Bacteroidetes	Actinobacteria	Chitinophaga sancti	Quadrisphaera granulorum	chemoheterotrophy_cellulolysis	unknown_unknown	positive	Candelaria concolor
Bac-670293	Bac-107	0.9165	0.001	Bacteroidetes	Bacteroidetes	Segetibacter aerophilus	Spirosoma sp	unknown_unknown	unknown_unknown	positive	Candelaria concolor
Bac-2316528	Bac-2714751	0.9181	0.001	Proteobacteria	Proteobacteria	Lichenibacterium minor	Lichenibacterium sp	unknown_unknown	unknown_unknown	positive	Candelaria concolor
Bac-57002	Bac-1892851	0.9206	0.001	Proteobacteria	Bacteroidetes	Amaricoccus tamworthensis	Flavitalea flava	aerobic chemoheterotrophy_unknown	unknown_unknown	positive	Candelaria concolor

Fung-3337	Fung-17303	0.9212	0.001	Ascomycota	Ascomycota	Knufia sp	Knufia sp	soil saprotroph_unknown	soil saprotroph_unknown	positive	Candelaria concolor
Fung-17304	Fung-58762	0.9213	0.001	Ascomycota	Basidiomycota	Knufia tsunedae	Chrysozymaceae Chrysozymaceae_sp	soil saprotroph_unknown	unknown_unknown	positive	Candelaria concolor
Bac-512383	Bac-940141	0.9216	0.001	Proteobacteria	Proteobacteria	Acidisoma sibiricum	Roseomonas riguiloci	aerobic chemoheterotrophy_unknown	animal parasites or symbionts_ureolysis	positive	Candelaria concolor
Fung-10115	Fung-576	0.9222	0.001	Ascomycota	Ascomycota	Chaetocapnodium sp	Unassigned	sooty mold_unknown	unknown_unknown	positive	Candelaria concolor
Bac-2316528	Bac-940139	0.9248	0.001	Proteobacteria	Acidobacteria	Lichenibacterium minor	Terriglobus aquaticus	unknown_unknown	aerobic chemoheterotrophy_unknown	positive	Candelaria concolor
Bac-512383	Bac-433	0.9263	0.001	Proteobacteria	Proteobacteria	Acidisoma sibiricum	Unassigned	aerobic chemoheterotrophy_unknown	unknown_unknown	positive	Candelaria concolor
Fung-17178	Fung-17268	0.9268	0.001	Ascomycota	Ascomycota	Exophiala xenobiotica	Paracladophialophora sp	animal parasite_unknown	plant pathogen_unknown	positive	Candelaria concolor
Bac-1189325	Bac-57002	0.9281	0.001	Proteobacteria	Proteobacteria	Oceanicella actignis	Amaricoccus tamworthensis	unknown_unknown	aerobic chemoheterotrophy_unknown	positive	Candelaria concolor
Fung-3674	Fung-20386	0.9296	0.001	Ascomycota	Ascomycota	Phaeophyscia sp	Phaeophyscia ciliata	lichenized_unknown	lichenized_unknown	positive	Candelaria concolor
Bac-670293	Bac-1983306	0.9336	0.001	Bacteroidetes	Bacteroidetes	Segetibacter aerophilus	Edaphobaculum flavum	unknown_unknown	unknown_unknown	positive	Candelaria concolor
Bac-223967	Bac-1055355	0.9341	0.001	Proteobacteria	Proteobacteria	Methylorubrum populi	Belnapia soli	unknown_unknown	unknown_unknown	positive	Candelaria concolor
Fung-17030	Fung-196	0.9395	0.001	Ascomycota	Ascomycota	Cyphellophora sp	Unassigned	litter saprotroph_unknown	unknown_unknown	positive	Candelaria concolor
Bac-490567	Bac-540746	0.9398	0.001	Proteobacteria	Proteobacteria	Arenimonas sp	Rudaea cellulosilytica	unknown_unknown	unknown_unknown	positive	Candelaria concolor
Bac-670293	Bac-398037	0.9433	0.001	Bacteroidetes	Bacteroidetes	Segetibacter aerophilus	Segetibacter koreensis	unknown_unknown	aerobicchemoheterotrophy_unknown	positive	Candelaria concolor

Bac-490567	Bac-1849581	0.9447	0.001	Proteobacteria	Proteobacteria	Arenimonas sp	Dyella caseinilytica	unknown_unknown	unknown_unknown	positive	Candelaria concolor
Fung-196	Fung-17268	0.9503	0.001	Ascomycota	Ascomycota	Unassigned	Paracladophialophora sp	unknown_unknown	plant pathogen_unknown	positive	Candelaria concolor
Bac-2316527	Bac-940139	0.9548	0.001	Proteobacteria	Acidobacteria	Lichenibacterium ramalinae	Terriglobus aquaticus	unknown_unknown	aerobic chemoheterotrophy_unknown	positive	Candelaria concolor
Fung-17304	Fung-17303	0.9573	0.001	Ascomycota	Ascomycota	Knufia tsunedae	Knufia sp	soil saprotroph_unknown	soil saprotroph_unknown	positive	Candelaria concolor
Bac-540746	Bac-1849581	0.9633	0.001	Proteobacteria	Proteobacteria	Rudaea cellulosilytica	Dyella caseinilytica	unknown_unknown	unknown_unknown	positive	Candelaria concolor
Bac-433	Bac-940141	0.9725	0.001	Proteobacteria	Proteobacteria	Unassigned	Roseomonas riguiloci	unknown_unknown	animal parasites or symbionts_ureolysis	positive	Candelaria concolor
Bac-1126	Bac-2831758	0.9748	0.001	Cyanobacteria	Cyanobacteria	Microcystis aeruginosa	Unassigned	oxygenic photoautotroph_harmful algal blooms	unknown_unknown	positive	Candelaria concolor
Bac-1126	Bac-1117	0.9761	0.001	Cyanobacteria	Cyanobacteria	Microcystis aeruginosa	Unassigned	oxygenic photoautotroph_harmful algal blooms	unknown_unknown	positive	Candelaria concolor
Bac-1126	Bac-2219509	0.9797	0.002	Cyanobacteria	Cyanobacteria	Microcystis aeruginosa	Haloleptolyngbya elongata	oxygenic photoautotroph_harmful algal blooms	oxygenic photoautotroph_unknown	positive	Candelaria concolor
Bac-1126	Bac-693994	0.9807	0.002	Cyanobacteria	Cyanobacteria	Microcystis aeruginosa	Pseudoscillatoria coralii	oxygenic photoautotroph_harmful algal blooms	oxygenic photoautotroph_nitrogen fixation	positive	Candelaria concolor
Bac-2219509	Bac-2831758	0.9848	0.004	Cyanobacteria	Cyanobacteria	Haloleptolyngbya elongata	Unassigned	oxygenic photoautotroph_unknown	unknown_unknown	positive	Candelaria concolor
Bac-1117	Bac-2831758	0.9854	0.001	Cyanobacteria	Cyanobacteria	Unassigned	Unassigned	unknown_unknown	unknown_unknown	positive	Candelaria concolor
Bac-693994	Bac-2831758	0.9886	0.001	Cyanobacteria	Cyanobacteria	Pseudoscillatoria coralii	Unassigned	oxygenic photoautotroph_nitrogen fixation	unknown_unknown	positive	Candelaria concolor
Bac-2219509	Bac-1117	0.9921	0.001	Cyanobacteria	Cyanobacteria	Haloleptolyngbya elongata	Unassigned	oxygenic photoautotroph_unknown	unknown_unknown	positive	Candelaria concolor

Bac-1117	Bac-693994	0.9959	0.001	Cyanobacteria	Cyanobacteria	Unassigned	Pseudoscillatoria coralii	unknown_unknown	oxygenic photoautotroph_nitrogen fixation	positive	Candelaria concolor
Bac-2219509	Bac-693994	0.9961	0.003	Cyanobacteria	Cyanobacteria	Haloleptolyngbya elongata	Pseudoscillatoria coralii	oxygenic photoautotroph_unknown	oxygenic photoautotroph_nitrogen fixation	positive	Candelaria concolor
Fung-9291	Bac-534635	-0.8387	0.001	Ascomycota	Bacteroidetes	Ascomycota Ascomycota_sp	Mucilaginibacter rigui	unknown	unknown	negative	Xanthoria parietina
Fung-26271	Bac-2077091	-0.8338	0.001	Ascomycota	Bacteroidetes	Helotiales Helotiales_sp	Flavipsychrobacter stenotrophus	unknown	unknown	negative	Xanthoria parietina
Fung-10986	Bac-518753	-0.8322	0.001	Ascomycota	Verrucomicrobia	Petrophila sp	Luteolibacter sp	unspecified saprotroph	unknown	negative	Xanthoria parietina
Fung-12314	Bac-1237077	-0.831	0.001	Ascomycota	Proteobacteria	Devriesia sp	Cellvibrio zantedeschiae	plant pathogen	unknown	negative	Xanthoria parietina
Fung-10986	Bac-2315688	-0.815	0.001	Ascomycota	Proteobacteria	Petrophila sp	Simplicispira hankyongi	unspecified saprotroph	unknown_unknown	negative	Xanthoria parietina
Fung-26271	Bac-49	-0.8124	0.001	Ascomycota	Proteobacteria	Helotiales Helotiales_sp	Unassigned	unknown	unknown	negative	Xanthoria parietina
Fung-11175	Bac-2315688	-0.8081	0.001	Ascomycota	Proteobacteria	Distocercospora pachyderma	Simplicispira hankyongi	plant pathogen	unknown_unknown	negative	Xanthoria parietina
Fung-26271	Bac-767694	-0.8049	0.001	Ascomycota	Proteobacteria	Helotiales Helotiales_sp	Methylobacterium longum	unknown	methylotrophy_methanol oxidation	negative	Xanthoria parietina
Fung-15065	Bac-540746	-0.8049	0.001	Ascomycota	Proteobacteria	Phaeosphaeriaceae Phaeosphaeriaceae_sp	Rudaea cellulosilytica	unknown	unknown	negative	Xanthoria parietina
Fung-24903	Bac-1793872	-0.8	0.001	Ascomycota	Proteobacteria	Xanthoria parietina	Piscinibacterium candidicorallinum	lichenized	unknown_unknown	negative	Xanthoria parietina

		027									
Fung-4	Bac-888829	-0.8001	0.001	Ascomycota	Proteobacteria	Unassigned	Aetherobacter sp	unknown	unknown	negative	Xanthoria parietina
Fung-20444	Bac-2219509	0.8004	0.001	Ascomycota	Cyanobacteria	Physcia sp	Haloleptolyngbya elongata	lichenized	oxygenic photoautotroph_unknown	positive	Xanthoria parietina
Fung-23143	Bac-2219505	0.8057	0.001	Ascomycota	Cyanobacteria	Scoliciosporum fabisporum	Sodalinema komarekii	lichenized	oxygenic photoautotroph_unknown	positive	Xanthoria parietina
Fung-12314	Bac-2528642	0.8084	0.001	Ascomycota	Proteobacteria	Devriesia sp	Lichenihabitans psoromatis	plant pathogen	unknown	positive	Xanthoria parietina
Fung-10986	Bac-940139	0.8111	0.001	Ascomycota	Acidobacteria	Petrophila sp	Terriglobus aquaticus	unspecified saprotroph	aerobic chemoheterotrophy_unknown	positive	Xanthoria parietina
Fung-24903	Bac-2847114	0.8134	0.001	Ascomycota	Cyanobacteria	Xanthoria parietina	Alborzia kermanshahica	lichenized	oxygenic photoautotroph_unknown	positive	Xanthoria parietina
Fung-10146	Bac-1979270	0.8171	0.001	Ascomycota	Proteobacteria	Capnodiales Capnodiales_sp	Sphingomonas crocodyli	unknown	aerobic chemoheterotrophy_unknown	positive	Xanthoria parietina
Fung-10146	Bac-1874116	0.8187	0.001	Ascomycota	Actinobacteria	Capnodiales Capnodiales_sp	Allobranchiibius huperziae	unknown	unknown	positive	Xanthoria parietina
Fung-21748	Bac-2219509	0.8211	0.001	Ascomycota	Cyanobacteria	Flavopunctelia flaventior	Haloleptolyngbya elongata	lichenized	oxygenic photoautotroph_unknown	positive	Xanthoria parietina
Fung-17108	Bac-398037	0.8266	0.001	Ascomycota	Bacteroidetes	Cladophialophora sp	Segetibacter koreensis	soil saprotroph unknown	aerobic chemoheterotrophy_unknown	positive	Xanthoria parietina
Fung-15065	Bac-32056	0.8354	0.001	Ascomycota	Cyanobacteria	Phaeosphaeriaceae Phaeosphaeriaceae_sp	Calothrix desertica	unknown	oxygenic photoautotroph_nitrogen fixation	positive	Xanthoria parietina
Fung-3676	Bac-2219509	0.8381	0.001	Ascomycota	Cyanobacteria	Physcia sp	Haloleptolyngbya elongata	lichenized	oxygenic photoautotroph_unknown	positive	Xanthoria parietina
Fung-901	Bac-2774051	0.8434	0.001	Ascomycota	Cyanobacteria	Unassigned	Johannesbaptistia floridana	unknown	oxygenic photoautotroph_unknown	positive	Xanthoria parietina

Fung-12314	Bac-1874116	0.8435	0.001	Ascomycota	Actinobacteria	Devriesia sp	Allobranchiibius huperziae	plant pathogen	unknown	positive	Xanthoria parietina
Fung-20430	Bac-2219509	0.8766	0.001	Ascomycota	Cyanobacteria	Physcia dubia	Haloleptolyngbya elongata	lichenized	oxygenic photoautotroph_unknown	positive	Xanthoria parietina
Fung-24903	Bac-2774051	0.896	0.001	Ascomycota	Cyanobacteria	Xanthoria parietina	Johannesbaptistia floridana	lichenized	oxygenic photoautotroph_unknown	positive	Xanthoria parietina
Bac-1118	Bac-2799720	0.9001	0.001	Cyanobacteria	Cyanobacteria	Unassigned	Aegeococcus sp	unknown	unknown	positive	Xanthoria parietina
Bac-670293	Bac-398037	0.904	0.001	Bacteroidetes	Bacteroidetes	Segetibacter aerophilus	Segetibacter koreensis	unknown	aerobic chemoheterotrophy_unknown	positive	Xanthoria parietina
Bac-44473	Bac-1458934	0.9045	0.001	Cyanobacteria	Cyanobacteria	Gloeothece membranacea	Okeania lorea	oxygenic photoautotroph_unknown	oxygenic photoautotroph_unknown	positive	Xanthoria parietina
Bac-1117	Bac-2831758	0.9058	0.001	Cyanobacteria	Cyanobacteria	Unassigned	Unassigned	unknown	unknown	positive	Xanthoria parietina
Bac-540746	Bac-1849581	0.9067	0.001	Proteobacteria	Proteobacteria	Rudaea cellulosilytica	Dyella caseinilytica	unknown	unknown	positive	Xanthoria parietina
Bac-693994	Bac-2799720	0.9074	0.001	Cyanobacteria	Cyanobacteria	Pseudoscillatoria coralii	Aegeococcus sp	oxygenic photoautotroph_nitrogen fixation	unknown	positive	Xanthoria parietina
Fung-21748	Fung-3676	0.9077	0.001	Ascomycota	Ascomycota	Flavopunctelia flaventior	Physcia sp	lichenized	lichenized	positive	Xanthoria parietina
Bac-1118	Bac-1458934	0.9097	0.001	Cyanobacteria	Cyanobacteria	Unassigned	Okeania lorea	unknown	oxygenic photoautotroph_unknown	positive	Xanthoria parietina
Bac-1434001	Bac-433924	0.9102	0.001	Proteobacteria	Proteobacteria	Extensimonas sp	Pseudacidovorax intermedius	unknown	unknown	positive	Xanthoria parietina
Bac-889274	Bac-888829	0.9107	0.001	Proteobacteria	Proteobacteria	Polyangium sorediatum	Aetherobacter sp	unknown	unknown	positive	Xanthoria parietina
Bac-1117	Bac-693994	0.9128	0.001	Cyanobacteria	Cyanobacteria	Unassigned	Pseudoscillatoria coralii	unknown	oxygenic photoautotroph_nitrogen fixation	positive	Xanthoria parietina

Bac-1045927	Bac-693994	0.9132	0.001	Cyanobacteria	Cyanobacteria	Tapinothrix clintonii	Pseudoscillatoria coralii	oxygenic photoautotroph_unknown	oxygenic photoautotroph_nitrogen fixation	positive	Xanthoria parietina
Bac-1649476	Bac-1465490	0.917	0.001	Bacteroidetes	Bacteroidetes	Parafilimonas sp	Parafilimonas terrae	unknown	unknown	positive	Xanthoria parietina
Bac-49	Bac-888829	0.9176	0.001	Proteobacteria	Proteobacteria	Unassigned	Aetherobacter sp	unknown	unknown	positive	Xanthoria parietina
Bac-1237077	Bac-10	0.9194	0.001	Proteobacteria	Proteobacteria	Cellvibrio zantedeschiae	Cellvibrio sp	unknown	unknown	positive	Xanthoria parietina
Bac-1126	Bac-2799720	0.9196	0.001	Cyanobacteria	Cyanobacteria	Microcystis aeruginosa	Aegeococcus sp	oxygenic photoautotroph_unknown	unknown	positive	Xanthoria parietina
Bac-44473	Bac-2799720	0.9207	0.002	Cyanobacteria	Cyanobacteria	Gloeothece membranacea	Aegeococcus sp	oxygenic photoautotroph_unknown	unknown	positive	Xanthoria parietina
Bac-1126	Bac-1458934	0.9249	0.001	Cyanobacteria	Cyanobacteria	Microcystis aeruginosa	Okeania lorea	oxygenic photoautotroph_unknown	oxygenic photoautotroph_unknown	positive	Xanthoria parietina
Bac-2316528	Bac-2714751	0.9251	0.001	Proteobacteria	Proteobacteria	Lichenibacterium minor	Lichenibacterium sp	unknown	unknown	positive	Xanthoria parietina
Bac-1458934	Bac-2831758	0.9308	0.001	Cyanobacteria	Cyanobacteria	Okeania lorea	Unassigned	oxygenic photoautotroph_unknown	unknown	positive	Xanthoria parietina
Bac-1458934	Bac-693994	0.9319	0.001	Cyanobacteria	Cyanobacteria	Okeania lorea	Pseudoscillatoria coralii	oxygenic photoautotroph_unknown	oxygenic photoautotroph_nitrogen fixation	positive	Xanthoria parietina
Bac-2303523	Bac-118562	0.9348	0.001	Cyanobacteria	Cyanobacteria	Komarkovaea angustata	Arthrospira platensis	oxygenic photoautotroph_unknown	oxygenic photoautotroph_unknown	positive	Xanthoria parietina
Bac-2714751	Bac-2316527	0.9354	0.001	Proteobacteria	Proteobacteria	Lichenibacterium sp	Lichenibacterium ramalinae	unknown	unknown	positive	Xanthoria parietina
Bac-1004	Bac-317664	0.9358	0.001	Bacteroidetes	Actinobacteria	Chitinophaga sancti	Quadrisphaera granulorum	chemoheterotrophy_cellulolysis	unknown	positive	Xanthoria parietina
Bac-2831758	Bac-118562	0.937	0.001	Cyanobacteria	Cyanobacteria	Unassigned	Arthrospira platensis	unknown	oxygenic photoautotroph_unknown	positive	Xanthoria parietina

Bac-2303523	Bac-2831758	0.9404	0.001	Cyanobacteria	Cyanobacteria	Komarkovaea angustata	Unassigned	oxygenic photoautotroph_unknown	unknown	positive	Xanthoria parietina
Bac-44473	Bac-2303523	0.9445	0.001	Cyanobacteria	Cyanobacteria	Gloeothece membranacea	Komarkovaea angustata	oxygenic photoautotroph_unknown	oxygenic photoautotroph_unknown	positive	Xanthoria parietina
Bac-1118	Bac-2831758	0.946	0.001	Cyanobacteria	Cyanobacteria	Unassigned	Unassigned	unknown	unknown	positive	Xanthoria parietina
Bac-1118	Bac-2303523	0.9467	0.001	Cyanobacteria	Cyanobacteria	Unassigned	Komarkovaea angustata	unknown	oxygenic photoautotroph_unknown	positive	Xanthoria parietina
Bac-1263637	Bac-88374	0.9487	0.001	Actinobacteria	Actinobacteria	Microbacterium jejuense	Agromyces rhizosphaerae	aerobic_chemoheterotrophy_unknown	aerobic_chemoheterotrophy_unknown	positive	Xanthoria parietina
Bac-1126	Bac-2303523	0.9494	0.001	Cyanobacteria	Cyanobacteria	Microcystis aeruginosa	Komarkovaea angustata	oxygenic photoautotroph_unknown	oxygenic photoautotroph_unknown	positive	Xanthoria parietina
Bac-2316528	Bac-2316527	0.9544	0.001	Proteobacteria	Proteobacteria	Lichenibacterium minor	Lichenibacterium ramalinae	unknown	unknown	positive	Xanthoria parietina
Bac-44473	Bac-2831758	0.9556	0.002	Cyanobacteria	Cyanobacteria	Gloeothece membranacea	Unassigned	oxygenic photoautotroph_unknown	unknown	positive	Xanthoria parietina
Fung-21748	Fung-20430	0.9559	0.001	Ascomycota	Ascomycota	Flavopunctelia flaventior	Physcia dubia	lichenized	lichenized	positive	Xanthoria parietina
Bac-2303523	Bac-693994	0.9583	0.001	Cyanobacteria	Cyanobacteria	Komarkovaea angustata	Pseudoscillatoria coralii	oxygenic photoautotroph_unknown	oxygenic photoautotroph_nitrogen fixation	positive	Xanthoria parietina
Bac-1118	Bac-118562	0.9591	0.001	Cyanobacteria	Cyanobacteria	Unassigned	Arthrospira platensis	unknown	oxygenic photoautotroph_unknown	positive	Xanthoria parietina
Bac-1118	Bac-693994	0.9625	0.001	Cyanobacteria	Cyanobacteria	Unassigned	Pseudoscillatoria coralii	unknown	oxygenic photoautotroph_nitrogen fixation	positive	Xanthoria parietina
Fung-3676	Fung-20430	0.9628	0.001	Ascomycota	Ascomycota	Physcia sp	Physcia dubia	lichenized	lichenized	positive	Xanthoria parietina
Bac-693994	Bac-118562	0.9641	0.001	Cyanobacteria	Cyanobacteria	Pseudoscillatoria coralii	Arthrospira platensis	oxygenic photoautotroph_nitrogen fixation	oxygenic photoautotroph_unknown	positive	Xanthoria parietina

Bac-1126	Bac-2831758	0.9687	0.001	Cyanobacteria	Cyanobacteria	Microcystis aeruginosa	Unassigned	oxygenic photoautotroph_unknown	unknown	positive	Xanthoria parietina
Bac-1126	Bac-118562	0.975	0.001	Cyanobacteria	Cyanobacteria	Microcystis aeruginosa	Arthrospira platensis	oxygenic photoautotroph_unknown	oxygenic photoautotroph_unknown	positive	Xanthoria parietina
Bac-1118	Bac-44473	0.9761	0.001	Cyanobacteria	Cyanobacteria	Unassigned	Gloeothece membranacea	unknown	oxygenic photoautotroph_unknown	positive	Xanthoria parietina
Bac-44473	Bac-693994	0.9776	0.001	Cyanobacteria	Cyanobacteria	Gloeothece membranacea	Pseudoscillatoria coralii	oxygenic photoautotroph_unknown	oxygenic photoautotroph_nitrogen fixation	positive	Xanthoria parietina
Bac-1126	Bac-1118	0.9786	0.001	Cyanobacteria	Cyanobacteria	Microcystis aeruginosa	Unassigned	oxygenic photoautotroph_unknown	unknown	positive	Xanthoria parietina
Bac-44473	Bac-118562	0.9822	0.001	Cyanobacteria	Cyanobacteria	Gloeothece membranacea	Arthrospira platensis	oxygenic photoautotroph_unknown	oxygenic photoautotroph_unknown	positive	Xanthoria parietina
Bac-1126	Bac-693994	0.987	0.001	Cyanobacteria	Cyanobacteria	Microcystis aeruginosa	Pseudoscillatoria coralii	oxygenic photoautotroph_unknown	oxygenic photoautotroph_nitrogen fixation	positive	Xanthoria parietina
Bac-693994	Bac-2831758	0.9897	0.001	Cyanobacteria	Cyanobacteria	Pseudoscillatoria coralii	Unassigned	oxygenic photoautotroph_nitrogen fixation	unknown	positive	Xanthoria parietina
Bac-1126	Bac-44473	0.9955	0.001	Cyanobacteria	Cyanobacteria	Microcystis aeruginosa	Gloeothece membranacea	oxygenic photoautotroph_unknown	oxygenic photoautotroph_unknown	positive	Xanthoria parietina
Fung-20430	Bac-467091	-0.8568	0.001	Ascomycota	Actinobacteria	Physcia dubia	Ilumatobacter fluminis	lichenized	aerobic chemoheterotrophy	negative	Physcia adscendens
Fung-20444	Bac-467091	-0.8485	0.002	Ascomycota	Actinobacteria	Physcia sp	Ilumatobacter fluminis	lichenized	aerobic chemoheterotrophy	negative	Physcia adscendens
Fung-10550	Bac-356	-0.8476	0.001	Ascomycota	Proteobacteria	Aureobasidium pullulans	Unassigned	sooty_mold	unknown	negative	Physcia adscendens
Fung-20427	Bac-467091	-0.8427	0.001	Ascomycota	Actinobacteria	Physcia caesia	Ilumatobacter fluminis	lichenized	aerobic chemoheterotrophy	negative	Physcia adscendens

Fung-9291	Bac-34072	-0.8402	0.001	Ascomycota	Proteobacteria	Ascomycota Ascomycota_sp	Variovorax sp	unknown	unknown	negative	Physcia adscendens
Fung-20444	Bac-1391654	-0.8287	0.001	Ascomycota	Proteobacteria	Physcia sp	Labilithrix luteola	lichenized	unknown	negative	Physcia adscendens
Fung-10239	Bac-1310416	-0.8225	0.001	Ascomycota	Bacteroidetes	Cladosporium cladosporioides	Ferruginibacter yonginensis	litter saprotroph	aerobic chemoheterotrophy unknown	negative	Physcia adscendens
Fung-20441	Bac-467091	-0.8156	0.001	Ascomycota	Actinobacteria	Physcia orientostellaris	Ilumatobacter fluminis	lichenized	aerobic chemoheterotrophy	negative	Physcia adscendens
Fung-12210	Bac-1983306	-0.81	0.001	Ascomycota	Bacteroidetes	Neodevriesia stirlingiae	Edaphobaculum flavum	plant pathogen	unknown	negative	Physcia adscendens
Fung-21748	Bac-1391654	-0.8078	0.002	Ascomycota	Proteobacteria	Flavopunctelia flaventior	Labilithrix luteola	lichenized	unknown	negative	Physcia adscendens
Fung-20386	Bac-466153	-0.8076	0.002	Ascomycota	Planctomycetes	Phaeophyscia ciliata	Singulisphaera acidiphila	lichenized	aerobic chemoheterotrophy_xylanolysis	negative	Physcia adscendens
Fung-24994	Bac-2040274	-0.8056	0.001	Ascomycota	Proteobacteria	Umbilicaria esculenta	Sphingomonas antarctica	lichenized	aerobic chemoheterotrophy_unknown	negative	Physcia adscendens
Fung-855	Bac-1391654	-0.8052	0.001	Ascomycota	Proteobacteria	Unassigned	Labilithrix luteola	unknown	unknown	negative	Physcia adscendens
Fung-58762	Bac-1793872	-0.8038	0.001	Basidiomycota	Proteobacteria	Chrysozymaceae Chrysozymaceae_sp	Piscinibacterium candidicorallinum	unknown	unknown	negative	Physcia adscendens
Fung-22601	Bac-1126	0.8033	0.001	Ascomycota	Cyanobacteria	Micarea fallax	Microcystis aeruginosa	lichenized	oxygenic photoautotroph_toxin producing cyanobacteria	positive	Physcia adscendens

Fung-830	Bac-2799740	0.8058	0.001	Ascomycota	Cyanobacteria	Unassigned	Aegeococcus thureti	unknown	oxygenic photoautotroph_unknown	positive	Physcia adscendens
Fung-20444	Bac-2219509	0.8063	0.002	Ascomycota	Cyanobacteria	Physcia sp	Haloleptolyngbya elongata	lichenized	oxygenic photoautotroph_unknown	positive	Physcia adscendens
Fung-9291	Bac-512383	0.8094	0.002	Ascomycota	Proteobacteria	Ascomycota Ascomycota_sp	Acidisoma sibiricum	unknown	aerobic chemoheterotrophy_unknown	positive	Physcia adscendens
Fung-22601	Bac-1434046	0.8115	0.001	Ascomycota	Proteobacteria	Micarea fallax	Sphingorhabdus sp	lichenized	unknown	positive	Physcia adscendens
Fung-9291	Bac-433	0.8159	0.001	Ascomycota	Proteobacteria	Ascomycota Ascomycota_sp	Unassigned	unknown	unknown	positive	Physcia adscendens
Fung-1706	Bac-1890422	0.8213	0.002	Glomeromycota	Cyanobacteria	Unassigned	Unassigned	unknown	unknown	positive	Physcia adscendens
Fung-20430	Bac-102117	0.827	0.001	Ascomycota	Cyanobacteria	Physcia dubia	Oscillatoria sancta	lichenized	oxygenic photoautotroph_unknown	positive	Physcia adscendens
Fung-23143	Bac-470868	0.8309	0.001	Ascomycota	Proteobacteria	Scoliciosporum fabisporum	Achromobacter marplatensis	lichenized	aerobic chemoheterotrophy_denitrification	positive	Physcia adscendens
Fung-21748	Bac-2816234	0.8322	0.001	Ascomycota	Cyanobacteria	Flavopunctelia flaventior	Neochroococcus gongqingensis	lichenized	oxygenic photoautotroph_unknown	positive	Physcia adscendens
Fung-9291	Bac-940141	0.8396	0.001	Ascomycota	Proteobacteria	Ascomycota Ascomycota_sp	Roseomonas riguiloci	unknown	animal parasites or symbionts_ureolysis	positive	Physcia adscendens
Fung-21748	Bac-102117	0.8502	0.001	Ascomycota	Cyanobacteria	Flavopunctelia flaventior	Oscillatoria sancta	lichenized	oxygenic photoautotroph_unknown	positive	Physcia adscendens
Fung-22601	Bac-1890422	0.8503	0.001	Ascomycota	Cyanobacteria	Micarea fallax	Unassigned	lichenized	unknown	positive	Physcia adscendens
Fung-830	Bac-118562	0.864	0.001	Ascomycota	Cyanobacteria	Unassigned	Arthrospira platensis	unknown	oxygenic photoautotroph_unknown	positive	Physcia adscendens
Fung-21748	Bac-1458934	0.8771	0.001	Ascomycota	Cyanobacteria	Flavopunctelia flaventior	Okeania lorea	lichenized	unknown	positive	Physcia adscendens

Fung-830	Bac-2219509	0.8793	0.001	Ascomycota	Cyanobacteria	Unassigned	Haloleptolyngbya elongata	unknown	oxygenic photoautotroph_unknown	positive	Physcia adscendens
Fung-21748	Bac-2784132	0.8831	0.001	Ascomycota	Cyanobacteria	Flavopunctelia flaventior	Leptodesmis alaskaensis	lichenized	oxygenic photoautotroph_nitrogen fixation	positive	Physcia adscendens
Fung-21748	Bac-2799740	0.8883	0.001	Ascomycota	Cyanobacteria	Flavopunctelia flaventior	Aegeococcus thureti	lichenized	oxygenic photoautotroph_unknown	positive	Physcia adscendens
Fung-830	Bac-2831758	0.8902	0.001	Ascomycota	Cyanobacteria	Unassigned	Unassigned	unknown	unknown	positive	Physcia adscendens
Fung-830	Bac-693994	0.8905	0.001	Ascomycota	Cyanobacteria	Unassigned	Pseudoscillatoria coralii	unknown	oxygenic photoautotroph_nitrogen fixation	positive	Physcia adscendens
Fung-830	Bac-1117	0.8911	0.001	Ascomycota	Cyanobacteria	Unassigned	Unassigned	unknown	unknown	positive	Physcia adscendens
Fung-21748	Bac-118562	0.8942	0.001	Ascomycota	Cyanobacteria	Flavopunctelia flaventior	Arthrospira platensis	lichenized	oxygenic photoautotroph_unknown	positive	Physcia adscendens
Bac-102117	Bac-693994	0.9002	0.001	Cyanobacteria	Cyanobacteria	Oscillatoria sancta	Pseudoscillatoria coralii	oxygenic photoautotroph_unknown	oxygenic photoautotroph_nitrogen fixation	positive	Physcia adscendens
Bac-2219509	Bac-102117	0.9013	0.001	Cyanobacteria	Cyanobacteria	Haloleptolyngbya elongata	Oscillatoria sancta	oxygenic photoautotroph_unknown	oxygenic photoautotroph_unknown	positive	Physcia adscendens
Bac-693994	Bac-2799740	0.9019	0.001	Cyanobacteria	Cyanobacteria	Pseudoscillatoria coralii	Aegeococcus thureti	oxygenic photoautotroph_nitrogrn fixation	oxygenic photoautotroph_unknown	positive	Physcia adscendens
Fung-20430	Bac-118562	0.9036	0.001	Ascomycota	Cyanobacteria	Physcia dubia	Arthrospira platensis	lichenized	oxygenic photoautotroph_unknown	positive	Physcia adscendens
Fung-20430	Bac-118562	0.9036	0.001	Ascomycota	Cyanobacteria	Physcia dubia	Arthrospira platensis	lichenized	oxygenic photoautotroph_unknown	positive	Physcia adscendens
Fung-3674	Fung-20386	0.9074	0.001	Ascomycota	Ascomycota	Phaeophyscia sp	Phaeophyscia ciliata	lichenized	lichenized	positive	Physcia adscendens
Fung-3676	Fung-20445	0.9169	0.001	Ascomycota	Ascomycota	Physcia sp	Physcia stellaris	lichenized	lichenized	positive	Physcia adscendens

Fung-20430	Bac-2831758	0.9221	0.001	Ascomycota	Cyanobacteria	Physcia dubia	Unassigned	lichenized	unknown	positive	Physcia adscendens
Fung-20430	Bac-2831758	0.9221	0.001	Ascomycota	Cyanobacteria	Physcia dubia	Unassigned	lichenized	unknown	positive	Physcia adscendens
Fung-8574	Fung-59981	0.9326	0.001	Basidiomycota	Basidiomycota	Kockovaella sp	Tremellales Tremellales_sp	epiphyte	unknown	positive	Physcia adscendens
Fung-21748	Bac-2831758	0.9328	0.001	Ascomycota	Cyanobacteria	Flavopunctelia flaventior	Unassigned	lichenized	unknown	positive	Physcia adscendens
Fung-21748	Bac-2831758	0.9328	0.001	Ascomycota	Cyanobacteria	Flavopunctelia flaventior	Unassigned	lichenized	unknown	positive	Physcia adscendens
Fung-17304	Fung-17303	0.9346	0.001	Ascomycota	Ascomycota	Knufia tsunedae	Knufia sp	soil saprotroph	soil saprotroph	positive	Physcia adscendens
Bac-2831758	Bac-118562	0.9376	0.001	Cyanobacteria	Cyanobacteria	Unassigned	Arthrospira platensis	unknown	oxygenic photoautotroph_unknown	positive	Physcia adscendens
Bac-2219509	Bac-118562	0.9395	0.001	Cyanobacteria	Cyanobacteria	Haloleptolyngbya elongata	Arthrospira platensis	oxygenic photoautotroph_unknown	oxygenic photoautotroph_unknown	positive	Physcia adscendens
Fung-20430	Bac-1117	0.941	0.001	Ascomycota	Cyanobacteria	Physcia dubia	Unassigned	lichenized	unknown	positive	Physcia adscendens
Fung-20430	Bac-1117	0.941	0.001	Ascomycota	Cyanobacteria	Physcia dubia	Unassigned	lichenized	unknown	positive	Physcia adscendens
Bac-1117	Bac-118562	0.9423	0.001	Cyanobacteria	Cyanobacteria	Unassigned	Arthrospira platensis	unknown	oxygenic photoautotroph_unknown	positive	Physcia adscendens
Fung-20430	Bac-2219509	0.9444	0.001	Ascomycota	Cyanobacteria	Physcia dubia	Haloleptolyngbya elongata	lichenized	oxygenic photoautotroph_unknown	positive	Physcia adscendens
Fung-20430	Bac-2219509	0.9444	0.001	Ascomycota	Cyanobacteria	Physcia dubia	Haloleptolyngbya elongata	lichenized	oxygenic photoautotroph_unknown	positive	Physcia adscendens
Bac-693994	Bac-118562	0.9449	0.001	Cyanobacteria	Cyanobacteria	Pseudoscillatoria coralii	Arthrospira platensis	oxygenic photoautotroph_nitrogrn fixation	oxygenic photoautotroph_unknown	positive	Physcia adscendens

Fung-20430	Bac-693994	0.9453	0.001	Ascomycota	Cyanobacteria	Physcia dubia	Pseudoscillatoria coralii	lichenized	oxygenic photoautotroph_nitrogen fixation	positive	Physcia adscendens
Fung-20430	Bac-693994	0.9453	0.001	Ascomycota	Cyanobacteria	Physcia dubia	Pseudoscillatoria coralii	lichenized	oxygenic photoautotroph_nitrogen fixation	positive	Physcia adscendens
Fung-3676	Fung-20420	0.9566	0.001	Ascomycota	Ascomycota	Physcia sp	Physcia aipolia	lichenized	lichenized	positive	Physcia adscendens
Bac-433	Bac-940141	0.9583	0.001	Proteobacteria	Proteobacteria	Unassigned	Roseomonas riguiloci	unknown	animal parasites or symbionts_ureolysis	positive	Physcia adscendens
Fung-21748	Bac-1117	0.9592	0.001	Ascomycota	Cyanobacteria	Flavopunctelia flaventior	Unassigned	lichenized	unknown	positive	Physcia adscendens
Fung-21748	Bac-1117	0.9592	0.001	Ascomycota	Cyanobacteria	Flavopunctelia flaventior	Unassigned	lichenized	unknown	positive	Physcia adscendens
Bac-2219509	Bac-2831758	0.9622	0.001	Cyanobacteria	Cyanobacteria	Haloleptolyngbya elongata	Unassigned	oxygenic photoautotroph_unknown	unknown	positive	Physcia adscendens
Fung-21748	Bac-693994	0.9683	0.001	Ascomycota	Cyanobacteria	Flavopunctelia flaventior	Pseudoscillatoria coralii	lichenized	oxygenic photoautotroph_nitrogen fixation	positive	Physcia adscendens
Fung-21748	Bac-693994	0.9683	0.001	Ascomycota	Cyanobacteria	Flavopunctelia flaventior	Pseudoscillatoria coralii	lichenized	oxygenic photoautotroph_nitrogen fixation	positive	Physcia adscendens
Bac-1117	Bac-2831758	0.9689	0.001	Cyanobacteria	Cyanobacteria	Unassigned	Unassigned	unknown	unknown	positive	Physcia adscendens
Bac-693994	Bac-2831758	0.9695	0.001	Cyanobacteria	Cyanobacteria	Pseudoscillatoria coralii	Unassigned	oxygenic photoautotroph_nitrogrn fixation	unknown	positive	Physcia adscendens
Fung-21748	Bac-2219509	0.97	0.001	Ascomycota	Cyanobacteria	Flavopunctelia flaventior	Haloleptolyngbya elongata	lichenized	oxygenic photoautotroph_unknown	positive	Physcia adscendens
Fung-21748	Bac-2219509	0.97	0.001	Ascomycota	Cyanobacteria	Flavopunctelia flaventior	Haloleptolyngbya elongata	lichenized	oxygenic photoautotroph_unknown	positive	Physcia adscendens
Bac-2219509	Bac-1117	0.9884	0.001	Cyanobacteria	Cyanobacteria	Haloleptolyngbya elongata	Unassigned	oxygenic photoautotroph_unknown	unknown	positive	Physcia adscendens

Bac-1117	Bac-693994	0.9941	0.001	Cyanobacteria	Cyanobacteria	Unassigned	Pseudoscillatoria coralii	unknown	oxygenic photoautotroph_nitrogen fixation	positive	Physcia adscendens
Bac-2219509	Bac-693994	0.998	0.001	Cyanobacteria	Cyanobacteria	Haloleptolyngbya elongata	Pseudoscillatoria coralii	oxygenic photoautotroph_unknown	oxygenic photoautotroph_nitrogen fixation	positive	Physcia adscendens

Supplementary Table 6a

**Sample-id**	**external_sample_code**	**sample_type**	**temperature_zone**	**latitude**	**longitude**	**uhi_range**	**uhi_level**	**Light_availability**	**degreeofconcealment**	**DBH**	**Trafic**	**perceived_temperature**	**replicate**	**strip**	**analyses_name**	**target_microbes**	**urbanization_level**
T0130_1123	L_dB_loc3_2	soil	temperaturezone1	52.10751	4.40812	0.0C - 0.8C	0.199413	20	20	44	38	31	D	_	temperaturezone1_Tilia sp._D	Soil Bacteria	low_urbanized
T0130_1124	L_dB_loc3_1	soil	temperaturezone1	52.10758	4.40805	0.0C - 0.8C	0.208107	20	25	44	38	31	F	_	temperaturezone1_Tilia sp._F	Soil Bacteria	low_urbanized
T0130_1125	L_dB_loc3_3	soil	temperaturezone1	52.1077	4.40822	0.0C - 0.8C	0.201052	10	15	50	38	30	E	_	temperaturezone1_Tilia sp._E	Soil Bacteria	low_urbanized
T0130_1126	L_dB_loc2_3	soil	temperaturezone1	52.11555	4.41444	0.0C - 0.8C	0.043562	0	10	42	38	31	A	_	temperaturezone1_Tilia sp._A	Soil Bacteria	low_urbanized
T0130_1127	L_dB_loc2_2	soil	temperaturezone1	52.11555	4.41444	0.0C - 0.8C	0.043562	15	20	40	38	31	B	_	temperaturezone1_Tilia sp._B	Soil Bacteria	low_urbanized
T0130_1128	L_dB_loc2_1	soil	temperaturezone1	52.11557	4.41441	0.0C - 0.8C	0.043562	15	20	40	38	31	C	_	temperaturezone1_Tilia sp._C	Soil Bacteria	low_urbanized
T0130_1129	L_dB_loc4_3	soil	temperaturezone1	52.12288	4.46104	0.0C - 0.8C	0.404132	40	30	30	53	31	J	_	temperaturezone1_Tilia sp._J	Soil Bacteria	low_urbanized
T0130_1130	L_dB_loc4_2	soil	temperaturezone1	52.1229	4.46125	0.0C - 0.8C	0.37507	60	30	34	53	30	G	_	temperaturezone1_Tilia sp._G	Soil Bacteria	low_urbanized
T0130_1131	L_dB_loc4_1	soil	temperaturezone1	52.12296	4.46139	0.0C - 0.8C	0.377827	40	30	42	53	33	H	_	temperaturezone1_Tilia sp._H	Soil Bacteria	low_urbanized
T0130_1132	L_dB_loc1_1	soil	temperaturezone1	52.13606	4.4141	0.0C - 0.8C	0.403898	90	65	35	69	38	I	_	temperaturezone1_Tilia sp._I	Soil Bacteria	low_urbanized
T0130_1133	L_dB_loc1_2	soil	temperaturezone1	52.13614	4.41407	0.0C - 0.8C	0.423088	90	60	46	68	30	K	_	temperaturezone1_Tilia sp._K	Soil Bacteria	low_urbanized
T0130_1134	L_dB_loc1_3	soil	temperaturezone1	52.13625	4.41386	0.0C - 0.8C	0.432276	90	60	46	64	30	L	_	temperaturezone1_Tilia sp._L	Soil Bacteria	low_urbanized
T0130_1135	L_Y_3986_3	soil	temperaturezone2	52.14127	4.48038	0.8C - 1.6C	1.009377	50	60	41	54	31	F	_	temperaturezone2_Tilia europaea 'Euchlora'_F	Soil Bacteria	medium_urbanized
T0130_1136	L_Y_3986_1	soil	temperaturezone2	52.14134	4.4805	0.8C - 1.6C	1.01624	30	50	43	53	31	G	_	temperaturezone2_Tilia europaea 'Euchlora'_G	Soil Bacteria	medium_urbanized

T0130_1137	L_Y_3986_2	soil	temperaturezone2	52.1414	4.48047	0.8C - 1.6C	1.02406	30	50	50	51	31	H	_	temperaturezone2_Tilia europaea 'Euchlora'_H	Soil Bacteria	medium_urbanized
T0130_1138	L_B_4915_1	soil	temperaturezone1	52.14188	4.48902	0.0C - 0.8C	0.790967	90	25	52	61	31	AD	_	temperaturezone1_Tilia europaea_AD	Soil Bacteria	low_urbanized
T0130_1139	L_B_4915_2	soil	temperaturezone1	52.14194	4.48922	0.0C - 0.8C	0.721112	90	65	30	63	30	Y	_	temperaturezone1_Tilia europaea_Y	Soil Bacteria	low_urbanized
T0130_1140	L_B_4915_3	soil	temperaturezone1	52.14198	4.48935	0.0C - 0.8C	0.695394	90	65	55	62	34	U	_	temperaturezone1_Tilia europaea_U	Soil Bacteria	low_urbanized
T0130_1141	L_B_0818_3	soil	temperaturezone1	52.14455	4.44994	0.0C - 0.8C	0.773804	85	50	45	46	31	AB	_	temperaturezone1_Tilia cordata 'Erecta'_AB	Soil Bacteria	low_urbanized
T0130_1142	L_B_0818_2	soil	temperaturezone1	52.14461	4.44978	0.0C - 0.8C	0.780658	75	50	41	44	31	AC	_	temperaturezone1_Tilia cordata 'Erecta'_AC	Soil Bacteria	low_urbanized
T0130_1143	L_B_0818_1	soil	temperaturezone1	52.14467	4.44963	0.0C - 0.8C	0.736097	90	50	41	44	35	Z	_	temperaturezone1_Tilia cordata 'Erecta'_Z	Soil Bacteria	low_urbanized
T0130_1144	L_Y_2863_1	soil	temperaturezone2	52.14517	4.46885	0.8C - 1.6C	1.414347	50	95	31	42	31	U	_	temperaturezone2_Tilia europaea 'Euchlora'_U	Soil Bacteria	medium_urbanized
T0130_1145	L_Y_2863_2	soil	temperaturezone2	52.14518	4.46895	0.8C - 1.6C	1.429211	20	98	34	42	36	W	_	temperaturezone2_Tilia europaea 'Euchlora'_W	Soil Bacteria	medium_urbanized
T0130_1146	L_Y_2863_3	soil	temperaturezone2	52.14521	4.46911	0.8C - 1.6C	1.426994	20	98	45	41	31	V	_	temperaturezone2_Tilia europaea 'Euchlora'_V	Soil Bacteria	medium_urbanized
T0130_1147	L_Y_4630_1	soil	temperaturezone2	52.1453	4.4867	0.8C - 1.6C	1.453825	55	90	45	57	31	Z	_	temperaturezone2_Tilia europaea_Z	Soil Bacteria	medium_urbanized
T0130_1148	L_Y_4630_2	soil	temperaturezone2	52.14534	4.48722	0.8C - 1.6C	1.443159	50	80	38	59	31	Y	_	temperaturezone2_Tilia europaea_Y	Soil Bacteria	medium_urbanized
T0130_1149	L_Y_4630_3	soil	temperaturezone2	52.14536	4.48743	0.8C - 1.6C	1.439263	55	85	50	56	31	X	_	temperaturezone2_Tilia europaea_X	Soil Bacteria	medium_urbanized
T0130_1150	L_Y_6111_2	soil	temperaturezone2	52.14862	4.50064	0.8C - 1.6C	1.336682	90	80	40	52	31	O	_	temperaturezone2_Tilia cordata_O	Soil Bacteria	medium_urbanized
T0130_1151	L_Y_6111_1	soil	temperaturezone2	52.14865	4.50082	0.8C - 1.6C	1.369984	70	75	51	55	31	P	_	temperaturezone2_Tilia europaea_P	Soil Bacteria	medium_urbanized
T0130_1152	L_Y_6111_3	soil	temperaturezone2	52.14875	4.50099	0.8C - 1.6C	1.396426	70	75	53	54	31	Q	_	temperaturezone2_Tilia europaea_Q	Soil Bacteria	medium_urbanized
T0130_1153	L_Y_7131_1	soil	temperaturezone2	52.15029	4.51088	0.8C - 1.6C	1.106571	30	20	48	64	31	K	_	temperaturezone2_Tilia europaea 'Pallida'_K	Soil Bacteria	medium_urbanized
T0130_1154	L_Y_7131_2	soil	temperaturezone2	52.15037	4.51095	0.8C - 1.6C	1.082246	30	20	42	61	31	I	_	temperaturezone2_Tilia europaea 'Pallida'_I	Soil Bacteria	medium_urbanized
T0130_1155	L_Y_7131_3	soil	temperaturezone2	52.15045	4.51104	0.8C - 1.6C	1.082246	30	20	40	62	31	J	_	temperaturezone2_Tilia europaea 'Pallida'_J	Soil Bacteria	medium_urbanized
T0130_1156	L_O_5085_1	soil	temperaturezone3	52.15052	4.49125	1.6C - 2.5C	1.776493	70	90	39	48	38	K	_	temperaturezone3_Tilia cordata_K	Soil Bacteria	highly_urbanized

T0130_1157	L_O_5085_2	soil	temperaturezone3	52.15055	4.49064	1.6C - 2.5C	1.742526	70	98	40	48	31	H	_	temperaturezone3_Tilia cordata_H	Soil Bacteria	highly_urbanized
T0130_1158	L_O_5085_3	soil	temperaturezone3	52.15057	4.49044	1.6C - 2.5C	1.741737	80	98	49	49	31	G	_	temperaturezone3_Tilia cordata_G	Soil Bacteria	highly_urbanized
T0130_1159	L_O_4709_3	soil	temperaturezone2	52.15251	4.48683	0.8C - 1.6C	1.591154	75	45	40	53	32	AE	_	temperaturezone2_Tilia europaea_AE	Soil Bacteria	medium_urbanized
T0130_1160	L_O_4709_2	soil	temperaturezone3	52.15259	4.48701	1.6C - 2.5C	1.696241	70	45	50	54	32	E	_	temperaturezone3_Tilia europaea_E	Soil Bacteria	highly_urbanized
T0130_1161	L_O_5360_1	soil	temperaturezone3	52.15262	4.49383	1.6C - 2.5C	1.827937	70	85	46	67	31	N	_	temperaturezone3_Tilia europaea_N	Soil Bacteria	highly_urbanized
T0130_1162	L_O_4709_1	soil	temperaturezone3	52.15267	4.48721	1.6C - 2.5C	1.694039	70	45	41	55	32	D	_	temperaturezone3_Tilia europaea_D	Soil Bacteria	highly_urbanized
T0130_1163	L_O_5360_2	soil	temperaturezone3	52.15287	4.49375	1.6C - 2.5C	1.828056	60	80	45	68	31	O	_	temperaturezone3_Tilia europaea_O	Soil Bacteria	highly_urbanized
T0130_1164	L_O_5360_3	soil	temperaturezone3	52.15295	4.49372	1.6C - 2.5C	1.823829	60	80	47	68	31	M	_	temperaturezone3_Tilia europaea_M	Soil Bacteria	highly_urbanized
T0130_1165	L_O_5263_1	soil	temperaturezone3	52.15474	4.4925	1.6C - 2.5C	1.865428	20	98	43	58	32	P	_	temperaturezone3_Tilia europaea_P	Soil Bacteria	highly_urbanized
T0130_1166	L_dR_5635_3	soil	temperaturezone3	52.15495	4.49575	1.6C - 2.5C	2.000443	60	95	39	67	32	S	_	temperaturezone3_Tilia europaea_S	Soil Bacteria	highly_urbanized
T0130_1167	L_dR_5635_2	soil	temperaturezone3	52.15497	4.49596	1.6C - 2.5C	2.008161	60	95	50	67	32	V	_	temperaturezone3_Tilia europaea_V	Soil Bacteria	highly_urbanized
T0130_1168	L_dR_5635_1	soil	temperaturezone3	52.15498	4.49635	1.6C - 2.5C	1.998301	70	90	41	67	32	R	_	temperaturezone3_Tilia europaea_R	Soil Bacteria	highly_urbanized
T0130_1169	L_O_5263_2	soil	temperaturezone3	52.15546	4.4923	1.6C - 2.5C	1.805437	40	30	36	58	32	L	_	temperaturezone3_Tilia europaea_L	Soil Bacteria	highly_urbanized
T0130_1170	L_O_5263_3	soil	temperaturezone3	52.15556	4.49228	1.6C - 2.5C	1.762976	50	20	45	59	32	J	_	temperaturezone3_Tilia europaea_J	Soil Bacteria	highly_urbanized
T0130_1171	L_O_4514_3	soil	temperaturezone2	52.15736	4.48554	0.8C - 1.6C	1.577684	45	60	31	59	32	AC	_	temperaturezone2_Tilia europaea_AC	Soil Bacteria	medium_urbanized
T0130_1172	L_dR_5816_1	soil	temperaturezone3	52.1575	4.49804	1.6C - 2.5C	2.003956	20	98	44	49	32	T	_	temperaturezone3_Tilia europaea_T	Soil Bacteria	highly_urbanized
T0130_1173	L_dR_5444_1	soil	temperaturezone3	52.15754	4.49436	1.6C - 2.5C	2.009837	40	95	41	57	44	W	_	temperaturezone3_Tilia platyphyllos_W	Soil Bacteria	highly_urbanized
T0130_1174	L_dR_5444_2	soil	temperaturezone3	52.15758	4.4944	1.6C - 2.5C	2.009837	45	95	39	54	44	X	_	temperaturezone3_Tilia platyphyllos_X	Soil Bacteria	highly_urbanized
T0130_1175	L_dR_5444_3	soil	temperaturezone3	52.15761	4.49443	1.6C - 2.5C	2.023556	40	95	32	54	45	Z	_	temperaturezone3_Tilia platyphyllos_Z	Soil Bacteria	highly_urbanized
T0130_1176	L_dR_5816_2	soil	temperaturezone3	52.15765	4.4981	1.6C - 2.5C	2.02841	40	95	48	47	32	AB	_	temperaturezone3_Tilia europaea_AB	Soil Bacteria	highly_urbanized

T0130_1177	L_dR_5816_3	soil	temperaturezone3	52.15767	4.49824	1.6C - 2.5C	2.042112	40	90	40	51	32	AC	_	temperaturezone3_Tilia europaea_AC	Soil Bacteria	highly_urbanized
T0130_1178	L_O_4514_2	soil	temperaturezone3	52.15769	4.48538	1.6C - 2.5C	1.652769	45	60	31	60	32	A	_	temperaturezone3_Tilia europaea_A	Soil Bacteria	highly_urbanized
T0130_1179	L_O_4514_1	soil	temperaturezone3	52.15778	4.48535	1.6C - 2.5C	1.6843	50	60	34	60	32	C	_	temperaturezone3_Tilia europaea_C	Soil Bacteria	highly_urbanized
T0130_1180	L_dR_5907_1	soil	temperaturezone3	52.15847	4.49836	1.6C - 2.5C	1.991522	30	92	34	49	32	Q	_	temperaturezone3_Tilia cordata_Q	Soil Bacteria	highly_urbanized
T0130_1181	L_dR_5907_3	soil	temperaturezone3	52.15868	4.49859	1.6C - 2.5C	2.049154	60	95	40	52	32	AD	_	temperaturezone3_Tilia cordata_AD	Soil Bacteria	highly_urbanized
T0130_1182	L_dR_5907_2	soil	temperaturezone3	52.15883	4.49864	1.6C - 2.5C	2.062318	40	98	42	53	32	AE	_	temperaturezone3_Tilia europaea_AE	Soil Bacteria	highly_urbanized
T0130_1183	L_O_7208_3	soil	temperaturezone3	52.15919	4.51134	1.6C - 2.5C	1.666876	50	20	38	52	31	B	_	temperaturezone3_Tilia europaea_B	Soil Bacteria	highly_urbanized
T0130_1184	L_dR_5905_1	soil	temperaturezone3	52.15935	4.49887	1.6C - 2.5C	2.006179	45	90	43	60	32	U	_	temperaturezone3_Tilia europaea_U	Soil Bacteria	highly_urbanized
T0130_1185	L_dR_5905_2	soil	temperaturezone3	52.15936	4.49876	1.6C - 2.5C	2.012073	55	95	45	61	32	Y	_	temperaturezone3_Tilia europaea_Y	Soil Bacteria	highly_urbanized
T0130_1186	L_O_7208_2	soil	temperaturezone3	52.15944	4.51143	1.6C - 2.5C	1.718802	55	25	46	54	31	F	_	temperaturezone3_Tilia europaea_F	Soil Bacteria	highly_urbanized
T0130_1187	L_O_7208_1	soil	temperaturezone3	52.15948	4.51149	1.6C - 2.5C	1.750586	35	25	50	55	31	I	_	temperaturezone3_Tilia europaea_I	Soil Bacteria	highly_urbanized
T0130_1188	L_dR_5905_3	soil	temperaturezone3	52.15979	4.49903	1.6C - 2.5C	2.024337	55	95	45	50	32	AA	_	temperaturezone3_Tilia europaea_AA	Soil Bacteria	highly_urbanized
T0130_1189	L_B_0880_3	soil	temperaturezone1	52.16127	4.45025	0.0C - 0.8C	0.625568	15	20	42	58	31	P	_	temperaturezone1_Tilia europaea 'Koningslinde'_P	Soil Bacteria	low_urbanized
T0130_1190	L_B_0880_2	soil	temperaturezone1	52.16141	4.4501	0.0C - 0.8C	0.605831	25	20	40	59	31	M	_	temperaturezone1_Tilia europaea 'Koningslinde'_M	Soil Bacteria	low_urbanized
T0130_1191	L_B_0880_1	soil	temperaturezone1	52.16147	4.45002	0.0C - 0.8C	0.605831	15	20	37	59	31	N	_	temperaturezone1_Tilia europaea 'Koningslinde'_N	Soil Bacteria	low_urbanized
T0130_1192	L_O_4683_1	soil	temperaturezone2	52.16668	4.48685	0.8C - 1.6C	1.585263	65	95	62	53	31	AD	_	temperaturezone2_Tilia platyphyllos_AD	Soil Bacteria	medium_urbanized
T0130_1193	L_O_4683_2	soil	temperaturezone2	52.16695	4.48689	0.8C - 1.6C	1.539386	30	95	68	48	31	AA	_	temperaturezone2_Tilia platyphyllos_AA	Soil Bacteria	medium_urbanized
T0130_1194	L_O_4683_3	soil	temperaturezone2	52.16707	4.48692	0.8C - 1.6C	1.540817	30	95	69	47	31	AB	_	temperaturezone2_Tilia platyphyllos_AB	Soil Bacteria	medium_urbanized
T0130_1195	L_Y_7473_2	soil	temperaturezone2	52.16729	4.51435	0.8C - 1.6C	1.413844	50	50	44	52	30	S	_	temperaturezone2_Tilia europaea 'Koningslinde'_S	Soil Bacteria	medium_urbanized
T0130_1196	L_Y_7473_3	soil	temperaturezone2	52.16729	4.51451	0.8C - 1.6C	1.408069	70	50	43	51	30	R	_	temperaturezone2_Tilia europaea 'Koningslinde'_R	Soil Bacteria	medium_urbanized

T0130_1197	L_Y_7473_1	soil	temperaturezone2	52.1673	4.51417	0.8C - 1.6C	1.413844	30	50	42	52	30	T	_	temperaturezone2_Tilia europaea 'Koningslinde'_T	Soil Bacteria	medium_urbanized
T0130_1198	L_Y_3657_1	soil	temperaturezone2	52.16861	4.47743	0.8C - 1.6C	1.239571	75	50	47	69	31	N	_	temperaturezone2_Tilia cordata_N	Soil Bacteria	medium_urbanized
T0130_1199	L_Y_3657_2	soil	temperaturezone2	52.16875	4.47748	0.8C - 1.6C	1.17184	65	45	39	70	31	L	_	temperaturezone2_Tilia cordata_L	Soil Bacteria	medium_urbanized
T0130_1200	L_Y_3657_3	soil	temperaturezone2	52.16884	4.4775	0.8C - 1.6C	1.17184	65	45	40	70	31	M	_	temperaturezone2_Tilia cordata_M	Soil Bacteria	medium_urbanized
T0130_1201	L_B_4021_1	soil	temperaturezone1	52.17322	4.48064	0.0C - 0.8C	0.799211	20	30	47	53	31	AE	_	temperaturezone1_Tilia europaea_AE	Soil Bacteria	low_urbanized
T0130_1202	L_B_4021_2	soil	temperaturezone2	52.17328	4.48067	0.8C - 1.6C	0.860474	20	30	47	55	31	A	_	temperaturezone2_Tilia europaea_A	Soil Bacteria	medium_urbanized
T0130_1203	L_B_4021_3	soil	temperaturezone2	52.17335	4.48071	0.8C - 1.6C	0.861035	20	35	40	52	31	B	_	temperaturezone2_Tilia europaea_B	Soil Bacteria	medium_urbanized
T0130_1204	L_dB_loc5_1	soil	temperaturezone1	52.1735	4.53269	0.0C - 0.8C	0.661393	50	40	30	61	40	R	_	temperaturezone1_Tilia cordata_R	Soil Bacteria	low_urbanized
T0130_1205	L_dB_loc5_2	soil	temperaturezone1	52.17358	4.53328	0.0C - 0.8C	0.646731	70	40	30	57	40	Q	_	temperaturezone1_Tilia cordata_Q	Soil Bacteria	low_urbanized
T0130_1206	L_dB_loc5_3	soil	temperaturezone1	52.17368	4.53346	0.0C - 0.8C	0.611255	50	40	30	58	40	O	_	temperaturezone1_Tilia cordata_O	Soil Bacteria	low_urbanized
T0130_1207	L_B_3551_3	soil	temperaturezone1	52.17576	4.47629	0.0C - 0.8C	0.697936	20	30	65	47	31	V	_	temperaturezone1_Tilia europaea 'Koningslinde'_V	Soil Bacteria	low_urbanized
T0130_1208	L_B_3272_1	soil	temperaturezone2	52.17578	4.47347	0.8C - 1.6C	0.956248	50	70	49	66	31	C	_	temperaturezone2_Tilia europaea 'Zwarte linde'_C	Soil Bacteria	medium_urbanized
T0130_1209	L_B_3551_2	soil	temperaturezone1	52.17581	4.47617	0.0C - 0.8C	0.665489	20	20	62	47	31	S	_	temperaturezone1_Tilia europaea 'Koningslinde'_S	Soil Bacteria	low_urbanized
T0130_1210	L_B_3551_1	soil	temperaturezone1	52.17585	4.47605	0.0C - 0.8C	0.691763	10	20	70	47	31	T	_	temperaturezone1_Tilia europaea 'Koningslinde'_T	Soil Bacteria	low_urbanized
T0130_1211	L_B_3272_2	soil	temperaturezone2	52.17601	4.47377	0.8C - 1.6C	0.960576	40	75	49	66	31	D	_	temperaturezone2_Tilia europaea 'Zwarte linde'_D	Soil Bacteria	medium_urbanized
T0130_1212	L_B_3272_3	soil	temperaturezone2	52.17607	4.47386	0.8C - 1.6C	0.960576	40	75	52	65	31	E	_	temperaturezone2_Tilia europaea 'Zwarte linde'_E	Soil Bacteria	medium_urbanized
T0130_1213	L_B_6979_3	soil	temperaturezone1	52.18283	4.50991	0.0C - 0.8C	0.74012	50	85	39	41	30	AA	_	temperaturezone1_Tilia europaea_AA	Soil Bacteria	low_urbanized
T0130_1214	L_B_6979_2	soil	temperaturezone1	52.1829	4.50961	0.0C - 0.8C	0.6989	60	90	39	43	30	W	_	temperaturezone1_Tilia europaea_W	Soil Bacteria	low_urbanized
T0130_1215	L_B_6979_1	soil	temperaturezone1	52.18291	4.5098	0.0C - 0.8C	0.706852	20	80	37	42	30	X	_	temperaturezone1_Tilia europaea_X	Soil Bacteria	low_urbanized
T0130_1123	L_dB_loc3_2	soil	temperaturezone1	52.10751	4.40812	0.0C - 0.8C	0.199413	20	20	44	38	31	D	_	temperaturezone1_Tilia sp._D	Soil Fungi	low_urbanized

T0130_1124	L_dB_loc3_1	soil	temperaturezone1	52.10758	4.40805	0.0C - 0.8C	0.208107	20	25	44	38	31	F	_	temperaturezone1_Tilia sp._F	Soil Fungi	low_urbanized
T0130_1125	L_dB_loc3_3	soil	temperaturezone1	52.1077	4.40822	0.0C - 0.8C	0.201052	10	15	50	38	30	E	_	temperaturezone1_Tilia sp._E	Soil Fungi	low_urbanized
T0130_1126	L_dB_loc2_3	soil	temperaturezone1	52.11555	4.41444	0.0C - 0.8C	0.043562	0	10	42	38	31	A	_	temperaturezone1_Tilia sp._A	Soil Fungi	low_urbanized
T0130_1127	L_dB_loc2_2	soil	temperaturezone1	52.11555	4.41444	0.0C - 0.8C	0.043562	15	20	40	38	31	B	_	temperaturezone1_Tilia sp._B	Soil Fungi	low_urbanized
T0130_1128	L_dB_loc2_1	soil	temperaturezone1	52.11557	4.41441	0.0C - 0.8C	0.043562	15	20	40	38	31	C	_	temperaturezone1_Tilia sp._C	Soil Fungi	low_urbanized
T0130_1129	L_dB_loc4_3	soil	temperaturezone1	52.12288	4.46104	0.0C - 0.8C	0.404132	40	30	30	53	31	J	_	temperaturezone1_Tilia sp._J	Soil Fungi	low_urbanized
T0130_1130	L_dB_loc4_2	soil	temperaturezone1	52.1229	4.46125	0.0C - 0.8C	0.37507	60	30	34	53	30	G	_	temperaturezone1_Tilia sp._G	Soil Fungi	low_urbanized
T0130_1131	L_dB_loc4_1	soil	temperaturezone1	52.12296	4.46139	0.0C - 0.8C	0.377827	40	30	42	53	33	H	_	temperaturezone1_Tilia sp._H	Soil Fungi	low_urbanized
T0130_1132	L_dB_loc1_1	soil	temperaturezone1	52.13606	4.4141	0.0C - 0.8C	0.403898	90	65	35	69	38	I	_	temperaturezone1_Tilia sp._I	Soil Fungi	low_urbanized
T0130_1133	L_dB_loc1_2	soil	temperaturezone1	52.13614	4.41407	0.0C - 0.8C	0.423088	90	60	46	68	30	K	_	temperaturezone1_Tilia sp._K	Soil Fungi	low_urbanized
T0130_1134	L_dB_loc1_3	soil	temperaturezone1	52.13625	4.41386	0.0C - 0.8C	0.432276	90	60	46	64	30	L	_	temperaturezone1_Tilia sp._L	Soil Fungi	low_urbanized
T0130_1135	L_Y_3986_3	soil	temperaturezone2	52.14127	4.48038	0.8C - 1.6C	1.009377	50	60	41	54	31	F	_	temperaturezone2_Tilia europaea 'Euchlora'_F	Soil Fungi	medium_urbanized
T0130_1136	L_Y_3986_1	soil	temperaturezone2	52.14134	4.4805	0.8C - 1.6C	1.01624	30	50	43	53	31	G	_	temperaturezone2_Tilia europaea 'Euchlora'_G	Soil Fungi	medium_urbanized
T0130_1137	L_Y_3986_2	soil	temperaturezone2	52.1414	4.48047	0.8C - 1.6C	1.02406	30	50	50	51	31	H	_	temperaturezone2_Tilia europaea 'Euchlora'_H	Soil Fungi	medium_urbanized
T0130_1138	L_B_4915_1	soil	temperaturezone1	52.14188	4.48902	0.0C - 0.8C	0.790967	90	25	52	61	31	AD	_	temperaturezone1_Tilia europaea_AD	Soil Fungi	low_urbanized
T0130_1139	L_B_4915_2	soil	temperaturezone1	52.14194	4.48922	0.0C - 0.8C	0.721112	90	65	30	63	30	Y	_	temperaturezone1_Tilia europaea_Y	Soil Fungi	low_urbanized
T0130_1140	L_B_4915_3	soil	temperaturezone1	52.14198	4.48935	0.0C - 0.8C	0.695394	90	65	55	62	34	U	_	temperaturezone1_Tilia europaea_U	Soil Fungi	low_urbanized
T0130_1141	L_B_0818_3	soil	temperaturezone1	52.14455	4.44994	0.0C - 0.8C	0.773804	85	50	45	46	31	AB	_	temperaturezone1_Tilia cordata 'Erecta'_AB	Soil Fungi	low_urbanized
T0130_1142	L_B_0818_2	soil	temperaturezone1	52.14461	4.44978	0.0C - 0.8C	0.780658	75	50	41	44	31	AC	_	temperaturezone1_Tilia cordata 'Erecta'_AC	Soil Fungi	low_urbanized
T0130_1143	L_B_0818_1	soil	temperaturezone1	52.14467	4.44963	0.0C - 0.8C	0.736097	90	50	41	44	35	Z	_	temperaturezone1_Tilia cordata 'Erecta'_Z	Soil Fungi	low_urbanized

T0130_1144	L_Y_2863_1	soil	temperaturezone2	52.14517	4.46885	0.8C - 1.6C	1.414347	50	95	31	42	31	U	_	temperaturezone2_Tilia europaea 'Euchlora'_U	Soil Fungi	medium_urbanized
T0130_1145	L_Y_2863_2	soil	temperaturezone2	52.14518	4.46895	0.8C - 1.6C	1.429211	20	98	34	42	36	W	_	temperaturezone2_Tilia europaea 'Euchlora'_W	Soil Fungi	medium_urbanized
T0130_1146	L_Y_2863_3	soil	temperaturezone2	52.14521	4.46911	0.8C - 1.6C	1.426994	20	98	45	41	31	V	_	temperaturezone2_Tilia europaea 'Euchlora'_V	Soil Fungi	medium_urbanized
T0130_1147	L_Y_4630_1	soil	temperaturezone2	52.1453	4.4867	0.8C - 1.6C	1.453825	55	90	45	57	31	Z	_	temperaturezone2_Tilia europaea_Z	Soil Fungi	medium_urbanized
T0130_1148	L_Y_4630_2	soil	temperaturezone2	52.14534	4.48722	0.8C - 1.6C	1.443159	50	80	38	59	31	Y	_	temperaturezone2_Tilia europaea_Y	Soil Fungi	medium_urbanized
T0130_1149	L_Y_4630_3	soil	temperaturezone2	52.14536	4.48743	0.8C - 1.6C	1.439263	55	85	50	56	31	X	_	temperaturezone2_Tilia europaea_X	Soil Fungi	medium_urbanized
T0130_1150	L_Y_6111_2	soil	temperaturezone2	52.14862	4.50064	0.8C - 1.6C	1.336682	90	80	40	52	31	O	_	temperaturezone2_Tilia cordata_O	Soil Fungi	medium_urbanized
T0130_1151	L_Y_6111_1	soil	temperaturezone2	52.14865	4.50082	0.8C - 1.6C	1.369984	70	75	51	55	31	P	_	temperaturezone2_Tilia europaea_P	Soil Fungi	medium_urbanized
T0130_1152	L_Y_6111_3	soil	temperaturezone2	52.14875	4.50099	0.8C - 1.6C	1.396426	70	75	53	54	31	Q	_	temperaturezone2_Tilia europaea_Q	Soil Fungi	medium_urbanized
T0130_1153	L_Y_7131_1	soil	temperaturezone2	52.15029	4.51088	0.8C - 1.6C	1.106571	30	20	48	64	31	K	_	temperaturezone2_Tilia europaea 'Pallida'_K	Soil Fungi	medium_urbanized
T0130_1154	L_Y_7131_2	soil	temperaturezone2	52.15037	4.51095	0.8C - 1.6C	1.082246	30	20	42	61	31	I	_	temperaturezone2_Tilia europaea 'Pallida'_I	Soil Fungi	medium_urbanized
T0130_1155	L_Y_7131_3	soil	temperaturezone2	52.15045	4.51104	0.8C - 1.6C	1.082246	30	20	40	62	31	J	_	temperaturezone2_Tilia europaea 'Pallida'_J	Soil Fungi	medium_urbanized
T0130_1156	L_O_5085_1	soil	temperaturezone3	52.15052	4.49125	1.6C - 2.5C	1.776493	70	90	39	48	38	K	_	temperaturezone3_Tilia cordata_K	Soil Fungi	highly_urbanized
T0130_1157	L_O_5085_2	soil	temperaturezone3	52.15055	4.49064	1.6C - 2.5C	1.742526	70	98	40	48	31	H	_	temperaturezone3_Tilia cordata_H	Soil Fungi	highly_urbanized
T0130_1158	L_O_5085_3	soil	temperaturezone3	52.15057	4.49044	1.6C - 2.5C	1.741737	80	98	49	49	31	G	_	temperaturezone3_Tilia cordata_G	Soil Fungi	highly_urbanized
T0130_1159	L_O_4709_3	soil	temperaturezone2	52.15251	4.48683	0.8C - 1.6C	1.591154	75	45	40	53	32	AE	_	temperaturezone2_Tilia europaea_AE	Soil Fungi	medium_urbanized
T0130_1160	L_O_4709_2	soil	temperaturezone3	52.15259	4.48701	1.6C - 2.5C	1.696241	70	45	50	54	32	E	_	temperaturezone3_Tilia europaea_E	Soil Fungi	highly_urbanized
T0130_1161	L_O_5360_1	soil	temperaturezone3	52.15262	4.49383	1.6C - 2.5C	1.827937	70	85	46	67	31	N	_	temperaturezone3_Tilia europaea_N	Soil Fungi	highly_urbanized
T0130_1162	L_O_4709_1	soil	temperaturezone3	52.15267	4.48721	1.6C - 2.5C	1.694039	70	45	41	55	32	D	_	temperaturezone3_Tilia europaea_D	Soil Fungi	highly_urbanized
T0130_1163	L_O_5360_2	soil	temperaturezone3	52.15287	4.49375	1.6C - 2.5C	1.828056	60	80	45	68	31	O	_	temperaturezone3_Tilia europaea_O	Soil Fungi	highly_urbanized

T0130_1164	L_O_5360_3	soil	temperaturezone3	52.15295	4.49372	1.6C - 2.5C	1.823829	60	80	47	68	31	M	_	temperaturezone3_Tilia europaea_M	Soil Fungi	highly_urbanized
T0130_1165	L_O_5263_1	soil	temperaturezone3	52.15474	4.4925	1.6C - 2.5C	1.865428	20	98	43	58	32	P	_	temperaturezone3_Tilia europaea_P	Soil Fungi	highly_urbanized
T0130_1166	L_dR_5635_3	soil	temperaturezone3	52.15495	4.49575	1.6C - 2.5C	2.000443	60	95	39	67	32	S	_	temperaturezone3_Tilia europaea_S	Soil Fungi	highly_urbanized
T0130_1167	L_dR_5635_2	soil	temperaturezone3	52.15497	4.49596	1.6C - 2.5C	2.008161	60	95	50	67	32	V	_	temperaturezone3_Tilia europaea_V	Soil Fungi	highly_urbanized
T0130_1168	L_dR_5635_1	soil	temperaturezone3	52.15498	4.49635	1.6C - 2.5C	1.998301	70	90	41	67	32	R	_	temperaturezone3_Tilia europaea_R	Soil Fungi	highly_urbanized
T0130_1169	L_O_5263_2	soil	temperaturezone3	52.15546	4.4923	1.6C - 2.5C	1.805437	40	30	36	58	32	L	_	temperaturezone3_Tilia europaea_L	Soil Fungi	highly_urbanized
T0130_1170	L_O_5263_3	soil	temperaturezone3	52.15556	4.49228	1.6C - 2.5C	1.762976	50	20	45	59	32	J	_	temperaturezone3_Tilia europaea_J	Soil Fungi	highly_urbanized
T0130_1171	L_O_4514_3	soil	temperaturezone2	52.15736	4.48554	0.8C - 1.6C	1.577684	45	60	31	59	32	AC	_	temperaturezone2_Tilia europaea_AC	Soil Fungi	medium_urbanized
T0130_1172	L_dR_5816_1	soil	temperaturezone3	52.1575	4.49804	1.6C - 2.5C	2.003956	20	98	44	49	32	T	_	temperaturezone3_Tilia europaea_T	Soil Fungi	highly_urbanized
T0130_1173	L_dR_5444_1	soil	temperaturezone3	52.15754	4.49436	1.6C - 2.5C	2.009837	40	95	41	57	44	W	_	temperaturezone3_Tilia platyphyllos_W	Soil Fungi	highly_urbanized
T0130_1174	L_dR_5444_2	soil	temperaturezone3	52.15758	4.4944	1.6C - 2.5C	2.009837	45	95	39	54	44	X	_	temperaturezone3_Tilia platyphyllos_X	Soil Fungi	highly_urbanized
T0130_1175	L_dR_5444_3	soil	temperaturezone3	52.15761	4.49443	1.6C - 2.5C	2.023556	40	95	32	54	45	Z	_	temperaturezone3_Tilia platyphyllos_Z	Soil Fungi	highly_urbanized
T0130_1176	L_dR_5816_2	soil	temperaturezone3	52.15765	4.4981	1.6C - 2.5C	2.02841	40	95	48	47	32	AB	_	temperaturezone3_Tilia europaea_AB	Soil Fungi	highly_urbanized
T0130_1177	L_dR_5816_3	soil	temperaturezone3	52.15767	4.49824	1.6C - 2.5C	2.042112	40	90	40	51	32	AC	_	temperaturezone3_Tilia europaea_AC	Soil Fungi	highly_urbanized
T0130_1178	L_O_4514_2	soil	temperaturezone3	52.15769	4.48538	1.6C - 2.5C	1.652769	45	60	31	60	32	A	_	temperaturezone3_Tilia europaea_A	Soil Fungi	highly_urbanized
T0130_1179	L_O_4514_1	soil	temperaturezone3	52.15778	4.48535	1.6C - 2.5C	1.6843	50	60	34	60	32	C	_	temperaturezone3_Tilia europaea_C	Soil Fungi	highly_urbanized
T0130_1180	L_dR_5907_1	soil	temperaturezone3	52.15847	4.49836	1.6C - 2.5C	1.991522	30	92	34	49	32	Q	_	temperaturezone3_Tilia cordata_Q	Soil Fungi	highly_urbanized
T0130_1181	L_dR_5907_3	soil	temperaturezone3	52.15868	4.49859	1.6C - 2.5C	2.049154	60	95	40	52	32	AD	_	temperaturezone3_Tilia cordata_AD	Soil Fungi	highly_urbanized
T0130_1182	L_dR_5907_2	soil	temperaturezone3	52.15883	4.49864	1.6C - 2.5C	2.062318	40	98	42	53	32	AE	_	temperaturezone3_Tilia europaea_AE	Soil Fungi	highly_urbanized
T0130_1183	L_O_7208_3	soil	temperaturezone3	52.15919	4.51134	1.6C - 2.5C	1.666876	50	20	38	52	31	B	_	temperaturezone3_Tilia europaea_B	Soil Fungi	highly_urbanized

T0130_1184	L_dR_5905_1	soil	temperaturezone3	52.15935	4.49887	1.6C - 2.5C	2.006179	45	90	43	60	32	U	_	temperaturezone3_Tilia europaea_U	Soil Fungi	highly_urbanized
T0130_1185	L_dR_5905_2	soil	temperaturezone3	52.15936	4.49876	1.6C - 2.5C	2.012073	55	95	45	61	32	Y	_	temperaturezone3_Tilia europaea_Y	Soil Fungi	highly_urbanized
T0130_1186	L_O_7208_2	soil	temperaturezone3	52.15944	4.51143	1.6C - 2.5C	1.718802	55	25	46	54	31	F	_	temperaturezone3_Tilia europaea_F	Soil Fungi	highly_urbanized
T0130_1187	L_O_7208_1	soil	temperaturezone3	52.15948	4.51149	1.6C - 2.5C	1.750586	35	25	50	55	31	I	_	temperaturezone3_Tilia europaea_I	Soil Fungi	highly_urbanized
T0130_1188	L_dR_5905_3	soil	temperaturezone3	52.15979	4.49903	1.6C - 2.5C	2.024337	55	95	45	50	32	AA	_	temperaturezone3_Tilia europaea_AA	Soil Fungi	highly_urbanized
T0130_1189	L_B_0880_3	soil	temperaturezone1	52.16127	4.45025	0.0C - 0.8C	0.625568	15	20	42	58	31	P	_	temperaturezone1_Tilia europaea 'Koningslinde'_P	Soil Fungi	low_urbanized
T0130_1190	L_B_0880_2	soil	temperaturezone1	52.16141	4.4501	0.0C - 0.8C	0.605831	25	20	40	59	31	M	_	temperaturezone1_Tilia europaea 'Koningslinde'_M	Soil Fungi	low_urbanized
T0130_1191	L_B_0880_1	soil	temperaturezone1	52.16147	4.45002	0.0C - 0.8C	0.605831	15	20	37	59	31	N	_	temperaturezone1_Tilia europaea 'Koningslinde'_N	Soil Fungi	low_urbanized
T0130_1192	L_O_4683_1	soil	temperaturezone2	52.16668	4.48685	0.8C - 1.6C	1.585263	65	95	62	53	31	AD	_	temperaturezone2_Tilia platyphyllos_AD	Soil Fungi	medium_urbanized
T0130_1193	L_O_4683_2	soil	temperaturezone2	52.16695	4.48689	0.8C - 1.6C	1.539386	30	95	68	48	31	AA	_	temperaturezone2_Tilia platyphyllos_AA	Soil Fungi	medium_urbanized
T0130_1194	L_O_4683_3	soil	temperaturezone2	52.16707	4.48692	0.8C - 1.6C	1.540817	30	95	69	47	31	AB	_	temperaturezone2_Tilia platyphyllos_AB	Soil Fungi	medium_urbanized
T0130_1195	L_Y_7473_2	soil	temperaturezone2	52.16729	4.51435	0.8C - 1.6C	1.413844	50	50	44	52	30	S	_	temperaturezone2_Tilia europaea 'Koningslinde'_S	Soil Fungi	medium_urbanized
T0130_1196	L_Y_7473_3	soil	temperaturezone2	52.16729	4.51451	0.8C - 1.6C	1.408069	70	50	43	51	30	R	_	temperaturezone2_Tilia europaea 'Koningslinde'_R	Soil Fungi	medium_urbanized
T0130_1197	L_Y_7473_1	soil	temperaturezone2	52.1673	4.51417	0.8C - 1.6C	1.413844	30	50	42	52	30	T	_	temperaturezone2_Tilia europaea 'Koningslinde'_T	Soil Fungi	medium_urbanized
T0130_1198	L_Y_3657_1	soil	temperaturezone2	52.16861	4.47743	0.8C - 1.6C	1.239571	75	50	47	69	31	N	_	temperaturezone2_Tilia cordata_N	Soil Fungi	medium_urbanized
T0130_1199	L_Y_3657_2	soil	temperaturezone2	52.16875	4.47748	0.8C - 1.6C	1.17184	65	45	39	70	31	L	_	temperaturezone2_Tilia cordata_L	Soil Fungi	medium_urbanized
T0130_1200	L_Y_3657_3	soil	temperaturezone2	52.16884	4.4775	0.8C - 1.6C	1.17184	65	45	40	70	31	M	_	temperaturezone2_Tilia cordata_M	Soil Fungi	medium_urbanized
T0130_1201	L_B_4021_1	soil	temperaturezone1	52.17322	4.48064	0.0C - 0.8C	0.799211	20	30	47	53	31	AE	_	temperaturezone1_Tilia europaea_AE	Soil Fungi	low_urbanized
T0130_1202	L_B_4021_2	soil	temperaturezone2	52.17328	4.48067	0.8C - 1.6C	0.860474	20	30	47	55	31	A	_	temperaturezone2_Tilia europaea_A	Soil Fungi	medium_urbanized
T0130_1203	L_B_4021_3	soil	temperaturezone2	52.17335	4.48071	0.8C - 1.6C	0.861035	20	35	40	52	31	B	_	temperaturezone2_Tilia europaea_B	Soil Fungi	medium_urbanized

T0130_1204	L_dB_loc5_1	soil	temperaturezone1	52.1735	4.53269	0.0C - 0.8C	0.661393	50	40	30	61	40	R	_	temperaturezone1_Tilia cordata_R	Soil Fungi	low_urbanized
T0130_1205	L_dB_loc5_2	soil	temperaturezone1	52.17358	4.53328	0.0C - 0.8C	0.646731	70	40	30	57	40	Q	_	temperaturezone1_Tilia cordata_Q	Soil Fungi	low_urbanized
T0130_1206	L_dB_loc5_3	soil	temperaturezone1	52.17368	4.53346	0.0C - 0.8C	0.611255	50	40	30	58	40	O	_	temperaturezone1_Tilia cordata_O	Soil Fungi	low_urbanized
T0130_1207	L_B_3551_3	soil	temperaturezone1	52.17576	4.47629	0.0C - 0.8C	0.697936	20	30	65	47	31	V	_	temperaturezone1_Tilia europaea 'Koningslinde'_V	Soil Fungi	low_urbanized
T0130_1208	L_B_3272_1	soil	temperaturezone2	52.17578	4.47347	0.8C - 1.6C	0.956248	50	70	49	66	31	C	_	temperaturezone2_Tilia europaea 'Zwarte linde'_C	Soil Fungi	medium_urbanized
T0130_1209	L_B_3551_2	soil	temperaturezone1	52.17581	4.47617	0.0C - 0.8C	0.665489	20	20	62	47	31	S	_	temperaturezone1_Tilia europaea 'Koningslinde'_S	Soil Fungi	low_urbanized
T0130_1210	L_B_3551_1	soil	temperaturezone1	52.17585	4.47605	0.0C - 0.8C	0.691763	10	20	70	47	31	T	_	temperaturezone1_Tilia europaea 'Koningslinde'_T	Soil Fungi	low_urbanized
T0130_1211	L_B_3272_2	soil	temperaturezone2	52.17601	4.47377	0.8C - 1.6C	0.960576	40	75	49	66	31	D	_	temperaturezone2_Tilia europaea 'Zwarte linde'_D	Soil Fungi	medium_urbanized
T0130_1212	L_B_3272_3	soil	temperaturezone2	52.17607	4.47386	0.8C - 1.6C	0.960576	40	75	52	65	31	E	_	temperaturezone2_Tilia europaea 'Zwarte linde'_E	Soil Fungi	medium_urbanized
T0130_1213	L_B_6979_3	soil	temperaturezone1	52.18283	4.50991	0.0C - 0.8C	0.74012	50	85	39	41	30	AA	_	temperaturezone1_Tilia europaea_AA	Soil Fungi	low_urbanized
T0130_1214	L_B_6979_2	soil	temperaturezone1	52.1829	4.50961	0.0C - 0.8C	0.6989	60	90	39	43	30	W	_	temperaturezone1_Tilia europaea_W	Soil Fungi	low_urbanized
T0130_1215	L_B_6979_1	soil	temperaturezone1	52.18291	4.5098	0.0C - 0.8C	0.706852	20	80	37	42	30	X	_	temperaturezone1_Tilia europaea_X	Soil Fungi	low_urbanized

Supplementary Table 6b

Sample-id	external_sample_code	sample_type	temperature_zone	latitude	longitude	uhi_range	uhi_level	LiAV	DOC	DBH	Trafic	perceived_temperature	lichen_species	analysis_name	Strip	Replicate	urbanization_level	target_microbes
T0130_1237	L_dB_loc3_2	lichen	temperaturezone1	5210751	440812	0.0C - 0.8C	0.199413	20	20	44	38	31	Xanthoria parietina	temperaturezone1_Xanthoria parietina_E	_	E	low_urbanized	Soil Bacteria
T0130_1238	L_dB_loc3_1	lichen	temperaturezone1	5210758	440805	0.0C - 0.8C	0.208107	20	25	44	38	31	Xanthoria parietina	temperaturezone1_Xanthoria parietina_G	_	G	low_urbanized	Soil Bacteria
T0130_1239	L_dB_loc3_3	lichen	temperaturezone1	521077	440822	0.0C - 0.8C	0.201052	10	15	50	38	30	Xanthoria parietina	temperaturezone1_Xanthoria parietina_F	_	F	low_urbanized	Soil Bacteria

T0130_1240	L_B_4915_1	lichen	temperaturezone2	5214188	448902	0.8C - 1.6C	0.810967	90	25	52	61	31	Xanthoria parietina	temperaturezone2_Xanthoria parietina_A	_	A	medium_urbanized	Soil Bacteria
T0130_1241	L_B_4915_2	lichen	temperaturezone1	5214194	448922	0.0C - 0.8C	0.721112	90	65	30	63	30	Xanthoria parietina	temperaturezone1_Xanthoria parietina_L	_	L	low_urbanized	Soil Bacteria
T0130_1242	L_B_4915_3	lichen	temperaturezone1	5214198	448935	0.0C - 0.8C	0.695394	90	65	55	62	34	Xanthoria parietina	temperaturezone1_Xanthoria parietina_J	_	J	low_urbanized	Soil Bacteria
T0130_1243	L_Y_3657_1	lichen	temperaturezone2	5216861	447743	0.8C - 1.6C	1.239571	75	50	47	69	31	Xanthoria parietina	temperaturezone2_Xanthoria parietina_H	_	H	medium_urbanized	Soil Bacteria
T0130_1244	L_Y_3657_2	lichen	temperaturezone2	5216875	447748	0.8C - 1.6C	1.17184	65	45	39	70	31	Xanthoria parietina	temperaturezone2_Xanthoria parietina_F	_	F	medium_urbanized	Soil Bacteria
T0130_1245	L_Y_3657_3	lichen	temperaturezone2	5216884	44775	0.8C - 1.6C	1.17184	65	45	40	70	31	Xanthoria parietina	temperaturezone2_Xanthoria parietina_G	_	G	medium_urbanized	Soil Bacteria
T0130_1246	L_O_4709_3	lichen	temperaturezone2	5215251	448683	0.8C - 1.6C	1.631154	75	45	40	53	32	Xanthoria parietina	temperaturezone2_Xanthoria parietina_M	_	M	medium_urbanized	Soil Bacteria
T0130_1247	L_O_4709_2	lichen	temperaturezone3	5215259	448701	1.6C - 2.4C	1.696241	70	45	50	54	32	Xanthoria parietina	temperaturezone3_Xanthoria parietina_B	_	B	highly_urbanized	Soil Bacteria
T0130_1248	L_O_4709_1	lichen	temperaturezone3	5215267	448721	1.6C - 2.4C	1.694039	70	45	41	55	32	Xanthoria parietina	temperaturezone3_Xanthoria parietina_A	_	A	highly_urbanized	Soil Bacteria
T0130_1249	L_dR_5635_1	lichen	temperaturezone3	5215498	449635	1.6C - 2.4C	1.998301	70	90	41	67	32	Xanthoria parietina	temperaturezone3_Xanthoria parietina_G	_	G	highly_urbanized	Soil Bacteria
T0130_1250	L_dR_5905_1	lichen	temperaturezone3	5215935	449887	1.6C - 2.4C	2.006179	45	90	43	60	32	Xanthoria parietina	temperaturezone3_Xanthoria parietina_L	_	L	highly_urbanized	Soil Bacteria
T0130_1251	L_dR_5905_2	lichen	temperaturezone3	5215936	449876	1.6C - 2.4C	2.012073	55	95	45	61	32	Xanthoria parietina	temperaturezone3_Xanthoria parietina_N	_	N	highly_urbanized	Soil Bacteria
T0130_1088	L_dB_loc4_1	lichen	temperaturezone1	5212296	446139	0.0C - 0.8C	0.377827	40	30	42	53	33	Physcia adscendens	temperaturezone1_Physcia adscendens_H	_	H	low_urbanized	Soil Bacteria

T0130_1089	L_Y_3986_3	lichen	temperaturezone2	5214127	448038	0.8C - 1.6C	1.009377	50	60	41	54	31	Physcia adscendens	temperaturezone2_Physcia adscendens_D	_	D	medium_urbanized	Soil Bacteria
T0130_1090	L_Y_3986_1	lichen	temperaturezone2	5214134	44805	0.8C - 1.6C	1.01624	30	50	43	53	31	Physcia adscendens	temperaturezone2_Physcia adscendens_E	_	E	medium_urbanized	Soil Bacteria
T0130_1091	L_B_4915_1	lichen	temperaturezone2	5214188	448902	0.8C - 1.6C	0.810967	90	25	52	61	31	Physcia adscendens	temperaturezone2_Physcia adscendens_B	_	B	medium_urbanized	Soil Bacteria
T0130_1092	L_B_0818_2	lichen	temperaturezone1	5214461	444978	0.0C - 0.8C	0.780658	75	50	41	44	31	Physcia adscendens	temperaturezone1_Physcia adscendens_O	_	O	low_urbanized	Soil Bacteria
T0130_1093	L_O_5085_2	lichen	temperaturezone3	5215055	449064	1.6C - 2.4C	1.742526	70	98	40	48	31	Physcia adscendens	temperaturezone3_Physcia adscendens_D	_	D	highly_urbanized	Soil Bacteria
T0130_1094	L_dR_5635_3	lichen	temperaturezone3	5215495	449575	1.6C - 2.4C	2.000443	60	95	39	67	32	Physcia adscendens	temperaturezone3_Physcia adscendens_J	_	J	highly_urbanized	Soil Bacteria
T0130_1095	L_dR_5635_1	lichen	temperaturezone3	5215498	449635	1.6C - 2.4C	1.998301	70	90	41	67	32	Physcia adscendens	temperaturezone3_Physcia adscendens_H	_	H	highly_urbanized	Soil Bacteria
T0130_1096	L_O_7208_3	lichen	temperaturezone2	5215919	451134	0.8C - 1.6C	1.666876	50	20	38	52	31	Physcia adscendens	temperaturezone2_Physcia adscendens_N	_	N	medium_urbanized	Soil Bacteria
T0130_1097	L_dR_5905_1	lichen	temperaturezone3	5215935	449887	1.6C - 2.4C	2.006179	45	90	43	60	32	Physcia adscendens	temperaturezone3_Physcia adscendens_M	_	M	highly_urbanized	Soil Bacteria
T0130_1098	L_O_7208_1	lichen	temperaturezone3	5215948	451149	1.6C - 2.4C	1.750586	35	25	50	55	31	Physcia adscendens	temperaturezone3_Physcia adscendens_E	_	E	highly_urbanized	Soil Bacteria
T0130_1099	L_Y_3657_1	lichen	temperaturezone2	5216861	447743	0.8C - 1.6C	1.239571	75	50	47	69	31	Physcia adscendens	temperaturezone2_Physcia adscendens_I	_	I	medium_urbanized	Soil Bacteria
T0130_1100	L_dB_loc5_1	lichen	temperaturezone1	521735	453269	0.0C - 0.8C	0.066139	50	40	30	61	40	Physcia adscendens	temperaturezone1_Physcia adscendens_D	_	D	low_urbanized	Soil Bacteria
T0130_1101	L_dB_loc5_2	lichen	temperaturezone1	5217358	453328	0.0C - 0.8C	0.064673	70	40	30	57	40	Physcia adscendens	temperaturezone1_Physcia adscendens_B	_	B	low_urbanized	Soil Bacteria

T0130_1102	L_B_6979_2	lichen	temperaturezone1	521829	450961	0.0C - 0.8C	0.6989	60	90	39	43	30	Physcia adscendens	temperaturezone1_Physcia adscendens_K	_	K	low_urbanized	Soil Bacteria
T0130_1103	L_dB_loc4_3	lichen	temperaturezone1	5212288	446104	0.0C - 0.8C	0.440413	40	30	30	53	31	Candelaria concolor	temperaturezone1_Candelaria concolor_I	_	I	low_urbanized	Soil Bacteria
T0130_1104	L_B_4915_2	lichen	temperaturezone1	5214194	448922	0.0C - 0.8C	0.721112	90	65	30	63	30	Candelaria concolor	temperaturezone1_Candelaria concolor_M	_	M	low_urbanized	Soil Bacteria
T0130_1105	L_B_0818_3	lichen	temperaturezone1	5214455	444994	0.0C - 0.8C	0.773804	85	50	45	46	31	Candelaria concolor	temperaturezone1_Candelaria concolor_N	_	N	low_urbanized	Soil Bacteria
T0130_1106	L_Y_4630_3	lichen	temperaturezone2	5214536	448743	0.8C - 1.6C	1.439263	55	85	50	56	31	Candelaria concolor	temperaturezone2_Candelaria concolor_L	_	L	medium_urbanized	Soil Bacteria
T0130_1107	L_dR_5635_3	lichen	temperaturezone3	5215495	449575	1.6C - 2.4C	2.000443	60	95	39	67	32	Candelaria concolor	temperaturezone3_Candelaria concolor_K	_	K	highly_urbanized	Soil Bacteria
T0130_1108	L_dR_5635_1	lichen	temperaturezone3	5215498	449635	1.6C - 2.4C	1.998301	70	90	41	67	32	Candelaria concolor	temperaturezone3_Candelaria concolor_I	_	I	highly_urbanized	Soil Bacteria
T0130_1109	L_dR_5816_2	lichen	temperaturezone3	5215765	44981	1.6C - 2.4C	2.02841	40	95	48	47	32	Candelaria concolor	temperaturezone3_Candelaria concolor_O	_	O	highly_urbanized	Soil Bacteria
T0130_1110	L_O_7208_3	lichen	temperaturezone2	5215919	451134	1.6C - 2.4C	1.666876	50	20	38	52	31	Candelaria concolor	temperaturezone2_Candelaria concolor_O	_	O	medium_urbanized	Soil Bacteria
T0130_1111	L_O_7208_2	lichen	temperaturezone3	5215944	451143	1.6C - 2.4C	1.718802	55	25	46	54	31	Candelaria concolor	temperaturezone3_Candelaria concolor_C	_	C	highly_urbanized	Soil Bacteria
T0130_1112	L_O_7208_1	lichen	temperaturezone3	5215948	451149	1.6C - 2.4C	1.750586	35	25	50	55	31	Candelaria concolor	temperaturezone3_Candelaria concolor_F	_	F	highly_urbanized	Soil Bacteria
T0130_1113	L_Y_7473_3	lichen	temperaturezone2	5216729	451451	0.8C - 1.6C	1.408069	70	50	43	51	30	Candelaria concolor	temperaturezone2_Candelaria concolor_J	_	J	medium_urbanized	Soil Bacteria
T0130_1114	L_Y_7473_1	lichen	temperaturezone2	521673	451417	0.8C - 1.6C	1.413844	30	50	42	52	30	Candelaria concolor	temperaturezone2_Candelaria concolor_K	_	K	medium_urbanized	Soil Bacteria
T0130_1115	L_B_4021_1	lichen	temperaturezone2	5217322	448064	0.8C - 1.6C	0.849211	20	30	47	53	31	Candelaria concolor	temperaturezone2_Candelaria concolor_C	_	C	medium_urbanized	Soil Bacteria

T0130_1116	L_dB_loc5_2	lichen	temperaturezone1	5217358	453328	0.0C - 0.8C	0.064673	70	40	30	57	40	Candelaria concolor	temperaturezone1_Candelaria concolor_C	_	C	low_urbanized	Soil Bacteria
T0130_1117	L_dB_loc5_3	lichen	temperaturezone1	5217368	453346	0.0C - 0.8C	0.061126	50	40	30	58	40	Candelaria concolor	temperaturezone1_Candelaria concolor_A	_	A	low_urbanized	Soil Bacteria
T0130_1237	L_dB_loc3_2	lichen	temperaturezone1	5210751	440812	0.0C - 0.8C	0.199413	20	20	44	38	31	Xanthoria parietina	temperaturezone1_Xanthoria parietina_E	_	E	low_urbanized	Soil Fungi
T0130_1238	L_dB_loc3_1	lichen	temperaturezone1	5210758	440805	0.0C - 0.8C	0.208107	20	25	44	38	31	Xanthoria parietina	temperaturezone1_Xanthoria parietina_G	_	G	low_urbanized	Soil Fungi
T0130_1239	L_dB_loc3_3	lichen	temperaturezone1	521077	440822	0.0C - 0.8C	0.201052	10	15	50	38	30	Xanthoria parietina	temperaturezone1_Xanthoria parietina_F	_	F	low_urbanized	Soil Fungi
T0130_1240	L_B_4915_1	lichen	temperaturezone2	5214188	448902	0.8C - 1.6C	0.810967	90	25	52	61	31	Xanthoria parietina	temperaturezone2_Xanthoria parietina_A	_	A	medium_urbanized	Soil Fungi
T0130_1241	L_B_4915_2	lichen	temperaturezone1	5214194	448922	0.0C - 0.8C	0.721112	90	65	30	63	30	Xanthoria parietina	temperaturezone1_Xanthoria parietina_L	_	L	low_urbanized	Soil Fungi
T0130_1242	L_B_4915_3	lichen	temperaturezone1	5214198	448935	0.0C - 0.8C	0.695394	90	65	55	62	34	Xanthoria parietina	temperaturezone1_Xanthoria parietina_J	_	J	low_urbanized	Soil Fungi
T0130_1243	L_Y_3657_1	lichen	temperaturezone2	5216861	447743	0.8C - 1.6C	1.239571	75	50	47	69	31	Xanthoria parietina	temperaturezone2_Xanthoria parietina_H	_	H	medium_urbanized	Soil Fungi
T0130_1244	L_Y_3657_2	lichen	temperaturezone2	5216875	447748	0.8C - 1.6C	1.17184	65	45	39	70	31	Xanthoria parietina	temperaturezone2_Xanthoria parietina_F	_	F	medium_urbanized	Soil Fungi
T0130_1245	L_Y_3657_3	lichen	temperaturezone2	5216884	44775	0.8C - 1.6C	1.17184	65	45	40	70	31	Xanthoria parietina	temperaturezone2_Xanthoria parietina_G	_	G	medium_urbanized	Soil Fungi
T0130_1246	L_O_4709_3	lichen	temperaturezone2	5215251	448683	0.8C - 1.6C	1.631154	75	45	40	53	32	Xanthoria parietina	temperaturezone2_Xanthoria parietina_M	_	M	medium_urbanized	Soil Fungi
T0130_1247	L_O_4709_2	lichen	temperaturezone3	5215259	448701	1.6C - 2.4C	1.696241	70	45	50	54	32	Xanthoria parietina	temperaturezone3_Xanthoria parietina_B	_	B	highly_urbanized	Soil Fungi
T0130_1248	L_O_4709_1	lichen	temperaturezone3	5215267	448721	1.6C - 2.4C	1.694039	70	45	41	55	32	Xanthoria parietina	temperaturezone3_Xanthoria parietina_A	_	A	highly_urbanized	Soil Fungi

T0130_1249	L_dR_5635_1	lichen	temperaturezone3	5215498	449635	1.6C - 2.4C	1.998301	70	90	41	67	32	Xanthoria parietina	temperaturezone3_Xanthoria parietina_G	_	G	highly_urbanized	Soil Fungi
T0130_1250	L_dR_5905_1	lichen	temperaturezone3	5215935	449887	1.6C - 2.4C	2.006179	45	90	43	60	32	Xanthoria parietina	temperaturezone3_Xanthoria parietina_L	_	L	highly_urbanized	Soil Fungi
T0130_1251	L_dR_5905_2	lichen	temperaturezone3	5215936	449876	1.6C - 2.4C	2.012073	55	95	45	61	32	Xanthoria parietina	temperaturezone3_Xanthoria parietina_N	_	N	highly_urbanized	Soil Fungi
T0130_1088	L_dB_loc4_1	lichen	temperaturezone1	5212296	446139	0.0C - 0.8C	0.377827	40	30	42	53	33	Physcia adscendens	temperaturezone1_Physcia adscendens_H	_	H	low_urbanized	Soil Fungi
T0130_1089	L_Y_3986_3	lichen	temperaturezone2	5214127	448038	0.8C - 1.6C	1.009377	50	60	41	54	31	Physcia adscendens	temperaturezone2_Physcia adscendens_D	_	D	medium_urbanized	Soil Fungi
T0130_1090	L_Y_3986_1	lichen	temperaturezone2	5214134	44805	0.8C - 1.6C	1.01624	30	50	43	53	31	Physcia adscendens	temperaturezone2_Physcia adscendens_E	_	E	medium_urbanized	Soil Fungi
T0130_1091	L_B_4915_1	lichen	temperaturezone2	5214188	448902	0.8C - 1.6C	0.810967	90	25	52	61	31	Physcia adscendens	temperaturezone2_Physcia adscendens_B	_	B	medium_urbanized	Soil Fungi
T0130_1092	L_B_0818_2	lichen	temperaturezone1	5214461	444978	0.0C - 0.8C	0.780658	75	50	41	44	31	Physcia adscendens	temperaturezone1_Physcia adscendens_O	_	O	low_urbanized	Soil Fungi
T0130_1093	L_O_5085_2	lichen	temperaturezone3	5215055	449064	1.6C - 2.4C	1.742526	70	98	40	48	31	Physcia adscendens	temperaturezone3_Physcia adscendens_D	_	D	highly_urbanized	Soil Fungi
T0130_1094	L_dR_5635_3	lichen	temperaturezone3	5215495	449575	1.6C - 2.4C	2.000443	60	95	39	67	32	Physcia adscendens	temperaturezone3_Physcia adscendens_J	_	J	highly_urbanized	Soil Fungi
T0130_1095	L_dR_5635_1	lichen	temperaturezone3	5215498	449635	1.6C - 2.4C	1.998301	70	90	41	67	32	Physcia adscendens	temperaturezone3_Physcia adscendens_H	_	H	highly_urbanized	Soil Fungi
T0130_1096	L_O_7208_3	lichen	temperaturezone2	5215919	451134	0.8C - 1.6C	1.666876	50	20	38	52	31	Physcia adscendens	temperaturezone2_Physcia adscendens_N	_	N	medium_urbanized	Soil Fungi
T0130_1097	L_dR_5905_1	lichen	temperaturezone3	5215935	449887	1.6C - 2.4C	2.006179	45	90	43	60	32	Physcia adscendens	temperaturezone3_Physcia adscendens_M	_	M	highly_urbanized	Soil Fungi

T0130_1098	L_O_7208_1	lichen	temperaturezone3	5215948	451149	1.6C - 2.4C	1.750586	35	25	50	55	31	Physcia adscendens	temperaturezone3_Physcia adscendens_E	_	E	highly_urbanized	Soil Fungi
T0130_1099	L_Y_3657_1	lichen	temperaturezone2	5216861	447743	0.8C - 1.6C	1.239571	75	50	47	69	31	Physcia adscendens	temperaturezone2_Physcia adscendens_I	_	I	medium_urbanized	Soil Fungi
T0130_1100	L_dB_loc5_1	lichen	temperaturezone1	521735	453269	0.0C - 0.8C	0.066139	50	40	30	61	40	Physcia adscendens	temperaturezone1_Physcia adscendens_D	_	D	low_urbanized	Soil Fungi
T0130_1101	L_dB_loc5_2	lichen	temperaturezone1	5217358	453328	0.0C - 0.8C	0.064673	70	40	30	57	40	Physcia adscendens	temperaturezone1_Physcia adscendens_B	_	B	low_urbanized	Soil Fungi
T0130_1102	L_B_6979_2	lichen	temperaturezone1	521829	450961	0.0C - 0.8C	0.6989	60	90	39	43	30	Physcia adscendens	temperaturezone1_Physcia adscendens_K	_	K	low_urbanized	Soil Fungi
T0130_1103	L_dB_loc4_3	lichen	temperaturezone1	5212288	446104	0.0C - 0.8C	0.440413	40	30	30	53	31	Candelaria concolor	temperaturezone1_Candelaria concolor_I	_	I	low_urbanized	Soil Fungi
T0130_1104	L_B_4915_2	lichen	temperaturezone1	5214194	448922	0.0C - 0.8C	0.721112	90	65	30	63	30	Candelaria concolor	temperaturezone1_Candelaria concolor_M	_	M	low_urbanized	Soil Fungi
T0130_1105	L_B_0818_3	lichen	temperaturezone1	5214455	444994	0.0C - 0.8C	0.773804	85	50	45	46	31	Candelaria concolor	temperaturezone1_Candelaria concolor_N	_	N	low_urbanized	Soil Fungi
T0130_1106	L_Y_4630_3	lichen	temperaturezone2	5214536	448743	0.8C - 1.6C	1.439263	55	85	50	56	31	Candelaria concolor	temperaturezone2_Candelaria concolor_L	_	L	medium_urbanized	Soil Fungi
T0130_1107	L_dR_5635_3	lichen	temperaturezone3	5215495	449575	1.6C - 2.4C	2.000443	60	95	39	67	32	Candelaria concolor	temperaturezone3_Candelaria concolor_K	_	K	highly_urbanized	Soil Fungi
T0130_1108	L_dR_5635_1	lichen	temperaturezone3	5215498	449635	1.6C - 2.4C	1.998301	70	90	41	67	32	Candelaria concolor	temperaturezone3_Candelaria concolor_I	_	I	highly_urbanized	Soil Fungi
T0130_1109	L_dR_5816_2	lichen	temperaturezone3	5215765	44981	1.6C - 2.4C	2.02841	40	95	48	47	32	Candelaria concolor	temperaturezone3_Candelaria concolor_O	_	O	highly_urbanized	Soil Fungi
T0130_1110	L_O_7208_3	lichen	temperaturezone2	5215919	451134	1.6C - 2.4C	1.666876	50	20	38	52	31	Candelaria concolor	temperaturezone2_Candelaria concolor_O	_	O	medium_urbanized	Soil Fungi

T0130_1111	L_O_7208_2	lichen	temperaturezone3	5215944	451143	1.6C - 2.4C	1.718802	55	25	46	54	31	Candelaria concolor	temperaturezone3_Candelaria concolor_C	_	C	highly_urbanized	Soil Fungi
T0130_1112	L_O_7208_1	lichen	temperaturezone3	5215948	451149	1.6C - 2.4C	1.750586	35	25	50	55	31	Candelaria concolor	temperaturezone3_Candelaria concolor_F	_	F	highly_urbanized	Soil Fungi
T0130_1113	L_Y_7473_3	lichen	temperaturezone2	5216729	451451	0.8C - 1.6C	1.408069	70	50	43	51	30	Candelaria concolor	temperaturezone2_Candelaria concolor_J	_	J	medium_urbanized	Soil Fungi
T0130_1114	L_Y_7473_1	lichen	temperaturezone2	521673	451417	0.8C - 1.6C	1.413844	30	50	42	52	30	Candelaria concolor	temperaturezone2_Candelaria concolor_K	_	K	medium_urbanized	Soil Fungi
T0130_1115	L_B_4021_1	lichen	temperaturezone2	5217322	448064	0.8C - 1.6C	0.849211	20	30	47	53	31	Candelaria concolor	temperaturezone2_Candelaria concolor_C	_	C	medium_urbanized	Soil Fungi
T0130_1116	L_dB_loc5_2	lichen	temperaturezone1	5217358	453328	0.0C - 0.8C	0.064673	70	40	30	57	40	Candelaria concolor	temperaturezone1_Candelaria concolor_C	_	C	low_urbanized	Soil Fungi
T0130_1117	L_dB_loc5_3	lichen	temperaturezone1	5217368	453346	0.0C - 0.8C	0.061126	50	40	30	58	40	Candelaria concolor	temperaturezone1_Candelaria concolor_A	_	A	low_urbanized	Soil Fungi
